# A Review of *Shamosuchus* and *Paralligator* (Crocodyliformes, Neosuchia) from the Cretaceous of Asia

**DOI:** 10.1371/journal.pone.0118116

**Published:** 2015-02-25

**Authors:** Alan H. Turner

**Affiliations:** Department of Anatomical Sciences, Stony Brook University, Stony Brook, New York, United States of America; University of Pennsylvania, UNITED STATES

## Abstract

The crocodyliform *Shamosuchus* is known from numerous Late Cretaceous localities in southern and eastern Mongolia and fragmentary remains from Uzbekistan. Seven species of *Shamosuchus* have been named from six localities in Mongolia and three in Uzbekistan. Six species originally described as *Paralligator* were later referred to *Shamosuchus*. Only the type species, *Shamosuchus djadochtaensis* has been examined in detail. Many of the named species of *Shamosuchus* show striking similarity in size and cranial morphology but most are based on partial remains suggesting that the true species diversity is overestimated. A review of all species referred to *Shamosuchus* recognizes three valid taxa: *Shamosuchus djadochtaensis*, S. *gradilifrons*, and S. *major*. *Shamosuchus sungaricus*, *S*. *borealis*, and S. *karakalpakensis* are nomena dubia, whereas S. *ancestralis*, *S*. *ulgicus*, *S*. *tersus*, and S. *ulanicus* are junior subjective synonyms of S. *gradilifrons*. Phylogenetic analysis of 318 phenotypic characters recovers a Paralligatoridae clade consisting of *Shamosuchus*, *Rugosuchus*, *Batrachomimus*, Glen Rose Form, and *Wannchampsus*. *Shamosuchus* is non-monophyletic: S. *djadochtaensis* is near the base of Paralligatoridae whereas S. *gradilifrons* + S. *major* are the most deeply nested. The name *Paralligator* is resurrected for this clade. *Rugosuchus* and *Batrachomimus* are sister taxa to *Paralligator*. Paralligatoridae is closely related to *Theriosuchus*, hylaeochampsids and a speciose *Allodaposuchus* clade, which together are the sister group of *Borealosuchus* plus Crocodylia. These results support the presence of a diverse clade in eastern Asia and western North America throughout the Cretaceous with origins in the Late Jurassic.

## Introduction

Over the past decade or more, much of the attention paid to Mesozoic crocodyliforms has focused on the morphologically and taxonomically diverse notosuchian lineage. This lineage, largely endemic to Gondwanan continents, entails a diversity of small and large bodied terrestrial species. Comparatively less attention has been paid to Neosuchia, the crocodyliform lineage leading to, and inclusive of, modern crocodylians. Understanding the evolutionary history and biology of Mesozoic neosuchians is of critical importance because it bears on how we reconstruct the ancestral condition for Crocodylia, how we understand the biogeographic evolution of neosuchians, and whether, or to what extent, climate affected neosuchian diversification. At least some of the neosuchian groups currently known have Laurasian or restricted distributions within Laurasia. One such neosuchian is *Shamosuchus*, which is known from seven named species across numerous Late Cretaceous localities in southern and eastern Mongolia and fragmentary remains from Uzbekistan.

The holotype of *Shamosuchus djadochtaensis*, described by Mook [1], is an incomplete skull found at the Shabarakh Usu locality (Bayn Dzak or The Flaming Cliffs under current usage; Late Cretaceous, Djadokhta Formation [Fm.]) during the Third Asiatic Expedition of the American Museum of Natural History in 1923. Recently a crocodyliform specimen from another Djadokhta Fm. locality Ukhaa Tolgod [2,3,4] was referred to *S*. *djadochtaensis* and provided the basis for a detailed anatomical description and phylogenetic analysis of the species [5].

Konzhukova [6] described two small crocodyliforms from the Late Cretaceous of Mongolia, *Paralligator ancestralis* from the Nemegt Fm. and *Paralligator gradilifrons* from the older Bayanshiree Fm. The holotype specimen of *P*. *ancestralis* is well preserved but disassociated cranial material, whereas the holotype of *P*. *gradilifrons* consists of a nearly complete skull lacking the palate. Efimov [7,8] named three additional *Paralligator* species (*P*. *borealis*, *P*. *major*, *and P*. *ulgicus*) from subsequent specimens recovered from Bayanshiree Fm. localities in Mongolia and equivalent deposits in Uzbekistan. A fifth *Paralligator* species (*P*. *sungaricus*) was described from the Early Cretaceous of China [9].

**Fig 1:**
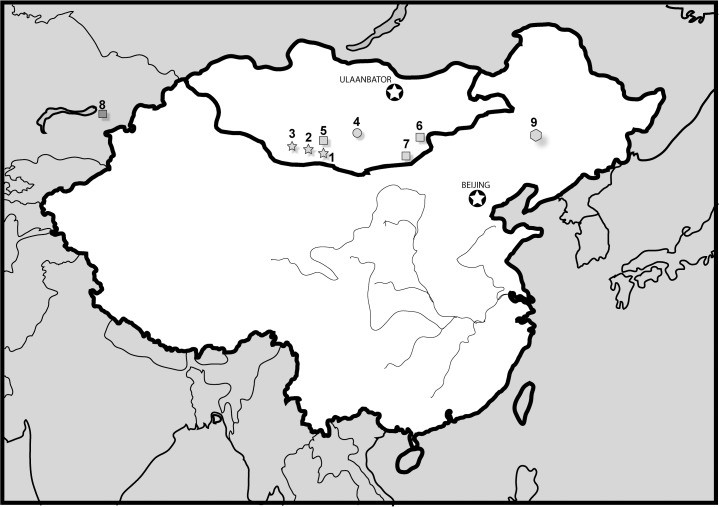
Geographic map showing the distribution of Asian paralligatorid species across China, Mongolia, and Uzbekistan. Stars = Nemegt Formation (Fm.) taxa; circles = Djadokhta Fm. taxa; squares = Bayanshiree Fm. (or equivalent) taxa; polygon = Lower Cretaceous. 1. *Shamosuchus ancestralis*; 2. *S. ulanicus*; 3. *S. tersus*; 4. *S. djadochtaensis*; 5. *S. gradilifrons*; 6. *S. major*; 7. *S. ulgicus*; 8. *S. borealis*; 9. *Rugosuchus nonganensis*.

Efimov [10] referred all *Paralligator* species to *Shamosuchus* and described a new species, *Shamosuchus occidentalis*. Three more *Shamosuchus* species were later described, two from the Nemegt Fm. of Mongolia (*S*. *tersus* and *S*. *ulanicus*) [11] and *Shamosuchus karakalpakensis* [12] from Uzbekistan. Efimov [13] considered *Shamosuchus occidentalis* a junior synonym of *S*. *borealis* leaving a total of ten named species of *Shamosuchus* from Cenomanian to Maastrichtian rocks in Uzbekistan, Mongolia, and China (Fig. 1).

The taxonomy of *Shamosuchus* and *Paralligator* is indeed quite complex (Table 1), and to date only *S*. *djadochtaensis* has been described in detail or included in a phylogenetic analysis. Many of the named species are known from limited material and of those well represented by numerous specimens there are considerable morphological differences in size and snout shape. The number of valid species may in fact be highly overestimated [5,14] and the congeneric status of *Shamosuchus* and *Paralligator* has been questioned [15]. Given the likely transitional placement of *Shamosuchus* near the base of Eusuchia [5], it is important to resolve the taxonomy and relationships of this group due to its impact on the phylogenetic placement of other eusuchian and basal crocodylian taxa.

**Table 1:** Overview of *Shamosuchus* and *Paralligator* taxonomic history.

Original Name	Authorship	Holotype	Other Names	Referring Author	Provenance	Status	Current Name
*Shamosuchus djadochtaensis*	Mook, 1924	AMNH FARB 6412	N/A	N/A	Djadokhta Fm., Mongolia	valid	*S*. *djadochtaensis*
*Paralligator ancestralis*	Konzhukova, 1954	PIN 551-29/1	*Shamosuchus*	Efimov, 1982	Nemegt Fm., Mongolia	subj junior syn	*P*. *gradilifrons*
*Paralligator gradilifrons*	Konzhukova, 1954	PIN 554-1	*Shamosuchus*	Efimov, 1982	Bayanshiree Fm., Mongolia	valid, new name	*P*. *gradilifrons*
*Paralligator sungaricus*	Sun, 1958	IVPP V2302	*Shamosuchus*	Efimov, 1982	Nenjiang Fm., Mongolia	nomen dubium	N/A
*Kansajsuchus borealis*	Efimov, 1975	PIN 372/702	*Shamosuchus*	Efimov, 1988?	Bissekty Fm., Uzbekistan	nomen dubium	N/A
*Paralligator major*	Efimov, 1981	PIN 3726/501	*Shamosuchus*	Efimov, 1982	Bayanshiree Fm., Mongolia	valid	*P*. *major*
*Paralligator ulgicus*	Efimov, 1981	PIN 3458/501	*Shamosuchus*	Efimov, 1982	Bayanshiree Fm., Mongolia	subj junior syn	*P*. *gradilifrons*
*Shamosuchus occidentalis*	Efimov, 1982	PIN 327/721	N/A	N/A	Bissekty Fm., Uzbekistan	nomen dubium	N/A
*Shamosuchus ulanicus*	Efimov, 1983	PIN 3140-502	N/A	N/A	Nemegt Fm., Mongolia	subj junior syn	*P*. *gradilifrons*
*Shamosuchus tersus*	Efimov, 1983	PIN 3141-501	N/A	N/A	Nemegt Fm., Mongolia	subj junior syn	*P*. *gradilifrons*
*Shamosuchus karakalpakensis*	Nesov et al., 1989	TsNIGRI 331/12457	N/A	N/A	Khodzhakul Fm., Uzbekistan	nomen dubium	N/A

Here I provide a detailed reassessment of species taxonomy of *Shamosuchus* and *Paralligator* and address the issue of their taxonomic status using a detailed phylogenetic analysis. I further discuss the phylogenetic relationships of these taxa both among themselves and with other advanced neosuchians near the origin of Eusuchia. I provide a revised taxonomy of *Shamosuchus* and *Paralligator* and discuss how improved taxon sampling among *Shamosuchus* and *Paralligator* species affects our understanding of the phylogenetic history of advanced neosuchians.

### Ethics Statement

No permits were required for the described study, which complied with all relevant regulations. For the comparative materials described here and listed in Appendix I in S1 Document. all required permissions were received. Institutional abbreviations are as follow: AMNH FARB, American Museum of Natural History, Collection of Fossil Reptiles, Amphibians, and Birds, New York; NHMUK, Natural History Museum, London, UK; IGM, Mongolian Institute of Geology, Ulaan Bataar, Mongolia; IGV, Geological Institute, Vertebrate Fossil Collections, Chinese Academy of Geological Sciences, Beijing, China; MCZ, Museum of Comparative Zoology, Harvard University, Cambridge; MTM, Hungarian Natural History Museum, Budapest, Hungary; PIN, Paleontological Institute Moscow, Russia; QM, Queensland Museum, Brisbane, Australia; SMU, Shuler Museum of Paleontology, Southern Methodist University, Dallas; TMM, Texas Memorial Museum, Austin; USNM, United States National Museum, Washington DC.

## Current understanding of *Shamosuchus*

### Definition and Diagnosis

When *Shamosuchus* was first named Mook [1] provided a brief list of generic characters as a diagnosis (p.1); “absence of mandibular foramen, prominent postero-external process of squamosal, exoccipital comprising a considerable portion of the condyle.” Mook ([1]: 1) cited additional specific-level characters, namely: “median ridge on frontal bone, prominent ridges on the lachrymals, medium size of supratemporal fenestrae, and their position close to the median line and far from the posterior external borders of the cranial table.”

Konzhukova [6] provided a genus-level diagnosis of *Paralligator* that reinforced features thought at the time to relate it to alligatorids such as a relatively short and broad skull with festooned margins, a flattened snout and an elevated skull table that is moderate in size, an external naris that is separated by paired anterior processes of the nasals, a frontal that does not reach the supratemporal fossae, and a “flattened” lower part of the mandible. It is not completely clear what Konzhukova meant by this last feature, but it could have been a reference to the crest that is present along the ventral margin of the mandible. Most of these features are broadly present among neosuchians and some, like the frontal failing to extend into the supratemporal fossae, were incorrectly interpreted by Konzhukova.

At least a couple of the features initially identified by Mook are plesiomorphic among neosuchians (e.g., the extent of the exoccipital participation in the occipital condyle and the smaller size of the supratemporal fenestrae). Some characters, such as the absence of a mandibular fenestra, are variable among neosuchians and require a parsimony analysis to determine if they indeed diagnose *Shamosuchus*. Nevertheless, the majority of Mook’s characters continue to diagnosis at least *Shamosuchus djadochtaensis* (see diagnosis in [5]). The main issue with the diagnosis of *Shamosuchus* is that to date none of the other putative *Shamosuchus* species have been included in a phylogenetic analysis. This, combined with the terrible state of *Shamosuchus* (and *Paralligator*) alpha taxonomy, leaves it unclear whether any of the features currently diagnosing *Shamosuchus djadochtaensis* actually diagnosis a more inclusive *Shamosuchus* clade. A cursory examination of the described *Shamosuchus* species suggests that at least a subset of these characters (in particular, features of the squamosal and sculpting of the snout and orbital region) will act as synapomorphies for a larger clade containing *Shamosuchus*.

### Relationships

Mook [1] did not guess at possible relationships of *Shamosuchus* in his initial description. He did, however, suggest that the form and position of the choanae were similar to modern eusuchians and not to “Mesosuchia”. This misinterpretation of the palate construction likely resulted from the poor preservation of the holotype specimen AMNH FARB 6412. Whereas the position of the choanae is posteriorly shifted like in eusuchians, it is not formed solely by the pterygoids as in eusuchians.

For a long time *Shamosuchus djadochtaensis* was considered a member of the Goniopholididae [16,17,18]. Buffetaut [19] placed the Paralligatoridae with Bernissartidae in his evolutionary tree of crocodyliforms, a placement that Efimov [13] seemed to accept. The first cladistic analyses of crocodyliforms affirmed the more derived neosuchian placement of *Shamosuchus* as a close relative to taxa like *Bernissartia fagesii* ([20]; Clark in [21]). Wu et al. [22] pointed out a number of derived similarities between *Shamosuchus* and *Rugosuchus nonganensis* from the Early Cretaceous of China.

Pol et al. [5] incorporated information from the holotype and the newly referred IGM 100/1195 specimen in a comprehensive phylogenetic analysis to test the relationship of *Shamosuchus djadochtaensis* to advanced neosuchian crocodyliforms. They confirmed the derived neosuchian status of *Shamosuchus djadochtaensis*. *Shamosuchus* and *Rugosuchus* were recovered as sister taxa and occupied a phylogenetic position closer to Eusuchia than either goniopholidids or *Bernissartia*. However, none of the other putative *Shamosuchus* species were considered in the analysis of Pol et al. [5]. Therefore, the monophyly and membership of *Shamosuchus* remains untested.

## Review of “*Shamosuchus*” and its putative relatives

### *Shamosuchus djadochtaensis* Mook, 1924 [1]

**Fig 2:**
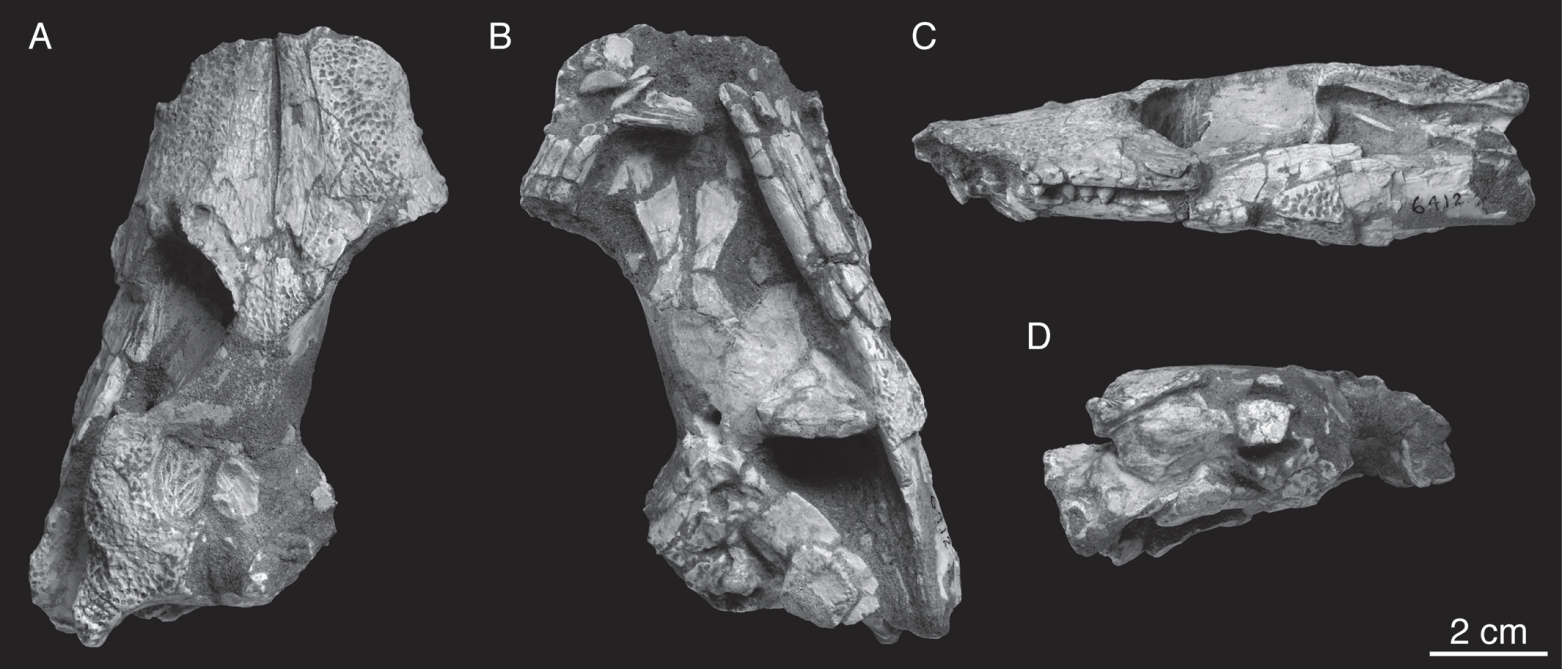
AMNH FARB 6412 (holotype), *Shamosuchus djadochtaensis*, Djadokhta Fm., Campanian, Mongolia. Photographs in **A**, dorsal, **B**, ventral, **C**, left lateral, **D**, occipital views.

**Fig 3:**
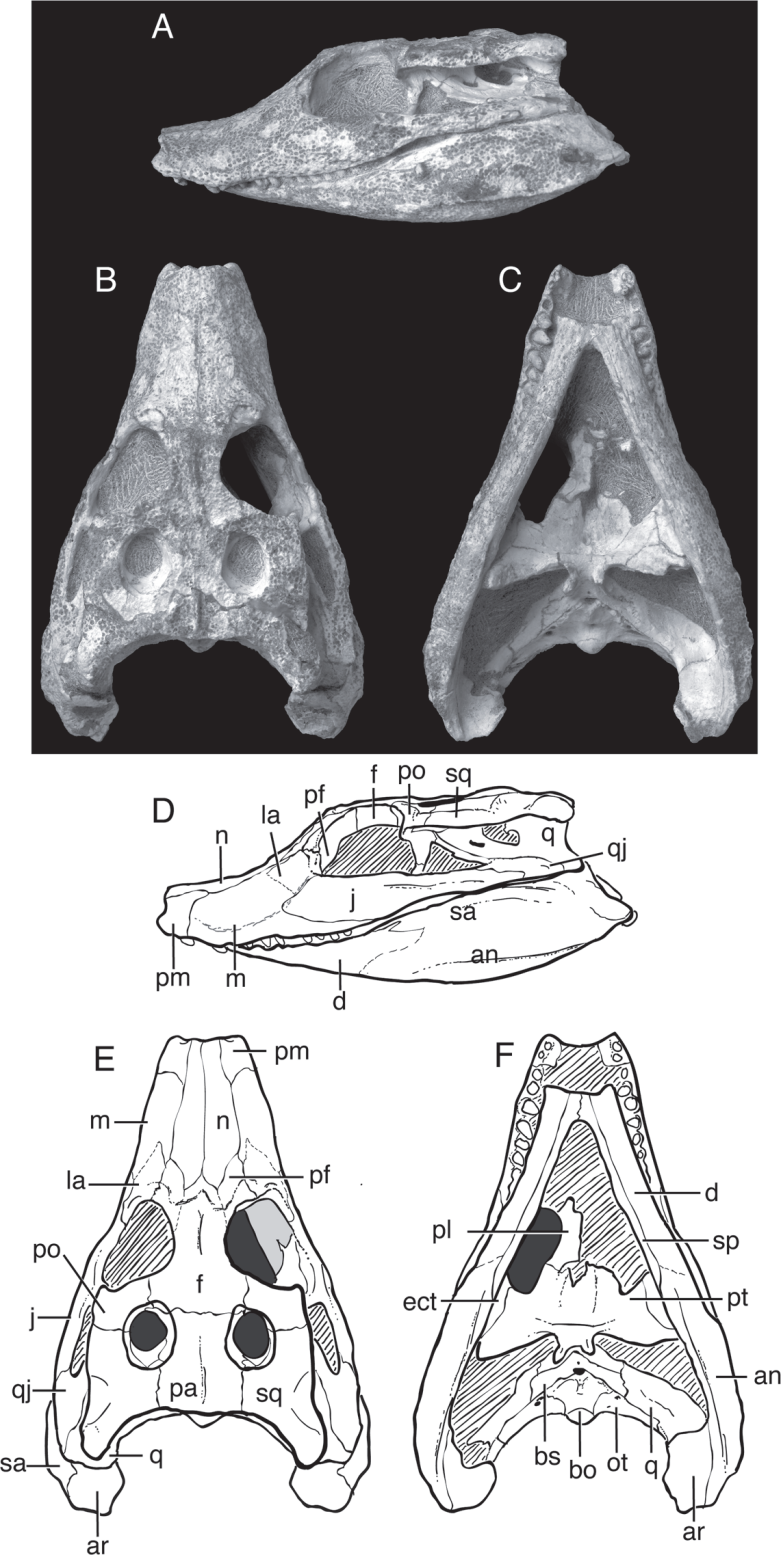
IGM 100/1195, *Shamosuchus djadochtaensis*, Djadokhta Fm., Campanian, Mongolia. Photographs in **A**, lateral, **B**, dorsal, **C**, ventral views. Line drawings in **D**, lateral, **E**, dorsal, **F**, ventral views. Abbreviations: an, angular; ar, articular; bo, basioccipital; bs, basisphenoid; d, dentary; ect, ectopteryoid; f, frontal; j, jugal; la, lacrimal; m, maxilla; n, nasal; ot, otoccipital; pa, parietal; pf, prefrontal; pl, palatine; pm, premaxilla; po, postorbital; pt, pterygoid; q, quadrate; qj, quadratojugal; sa, surangular; sp, splenial; sq, squamosal.

Mook [1] described *Shamosuchus djadochtaensis* on the basis of an incomplete and not entirely well preserved skull (AMNH FARB 6412, Fig. 2) from the Flaming Cliffs locality (Djadokhta Fm., Mongolia). Mook’s description was quite short and lacked much in the way of anatomical details. A second, much more complete specimen (IGM 100/1195) found at Ukhaa Tolgod is a nearly complete skull (Fig. 3) and associated postcranial skeleton, including representative osteoderms from across the entire dermal shield (i.e., dorsal, ventral, and appendicular). This specimen provided the basis of a much more detailed description of this species, accompanied by extensive figuring of the holotype and referred specimen [5]. Thus, there is little need to amend this description.

**Fig 4:**
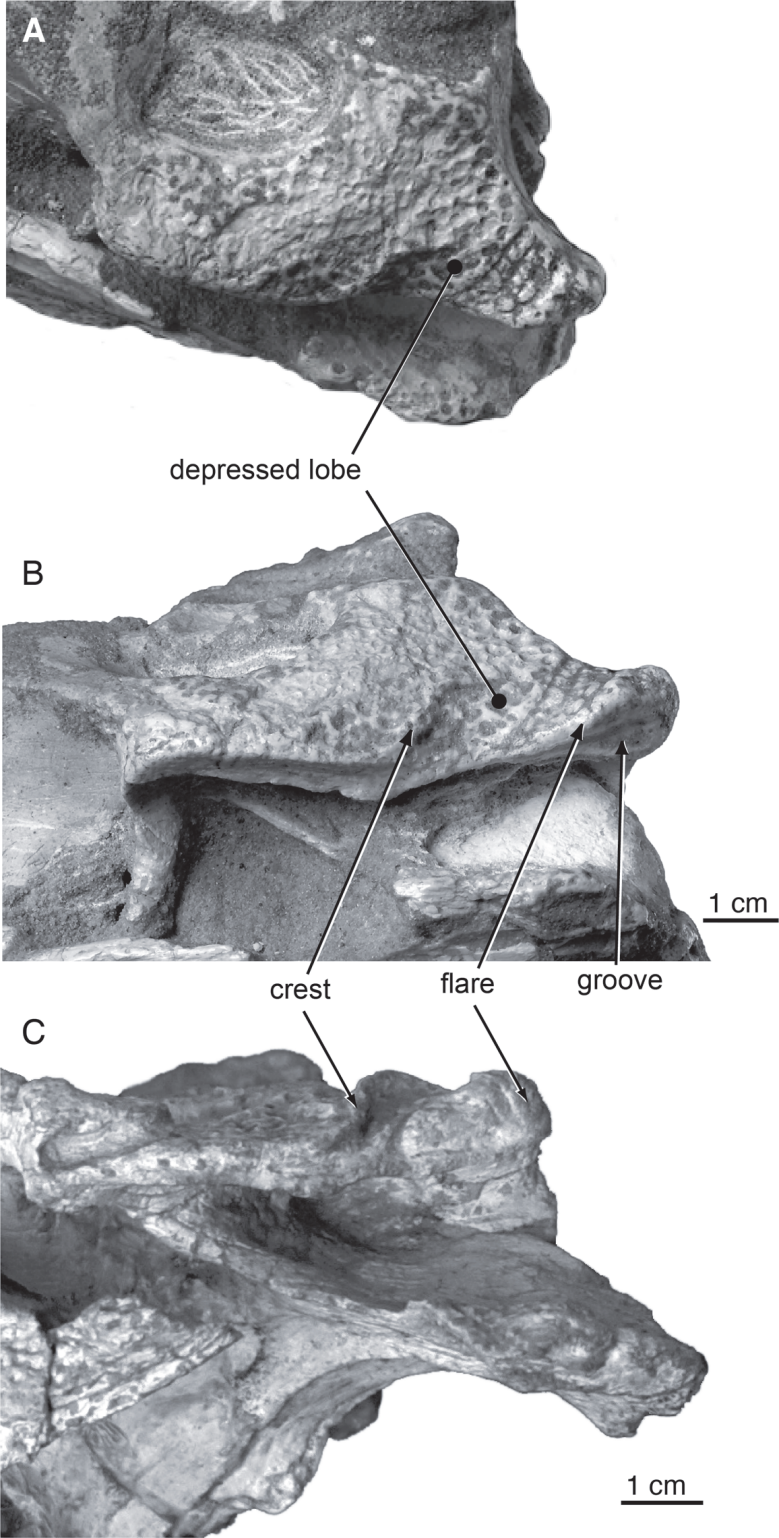
Skull morphology of Paralligatoridae. **A**, *Shamosuchus djadochtaensis*, AMNH FARB 6412, dorsal view; **B**, *S*. *djadochtaensis*, AMNH FARB 6412, left lateral view; **C**, *S*. *ulanicus* (= *Paralligator gradilifrons*), PIN 3140-502, left lateral view.

*Shamosuchus djadochtaensis* is known exclusively from the Campanian Djadokhta Fm. Pol et al. [5] provide a detailed diagnosis based on a combination of derived and autapomorphic characters. Autapomorphies for *S*. *djadochtaensis* include a dorsal surface of lacrimal and prefrontal that bears a smooth, rounded depression bounded by elevated ridges and a frontal with elevated orbital margins. The frontal participates in supratemporal fossa, as is common in many basal neosuchians. An additional, quite distinctive autapomorphy is the shallow and broad squamosal groove that tapers posteriorly at the level of the posterior edge of the otic aperture which then reappears along the lateral edge of the posterolateral process of the squamosal (Fig. 4B). This morphology produces a “flared” appearance to the lateral profile of the squamosal. *S*. *djadochtaensis* also is characterized by a narrow ascending process of the quadratojugal bearing a slightly developed ridge located close to its anterior margin, as well as cervical osteoderms with extremely large lateral keels located along the posterior margin of the osteoderm, possession of a set of osteoderms that are longer than they are wide, and dorsal osteoderms with keels restricted to the posterior margin.

*Shamosuchus djadochtaensis* is unquestionably a valid species and remains the only putative *Shamosuchus* described from the Djadokhta Fm. Because so little has been published on the morphology of the other *Shamosuchus* species, I consider it likely that some of the features listed above and currently understood to be autapomorphic for *S*. *djadochtaensis* may prove to be more widespread. Some of these features, therefore, may serve as synapomorphies uniting some or all of the *Shamosuchus* species considered here.

Even if many of the autapomorphies identified by Pol et al. [5] are more widely shared among *Shamosuchus* species, *S*. *djadochtaensis* remains distinguishable from all other putative *Shamosuchus* on the basis on the proportionally shorter and narrower snout. This shorter snout is not due to ontogeny. *Shamosuchus ulanicus* (PIN 3140/502-1) has the same skull table size (roughly 5 cm long by 6 cm width) but a snout nearly twice the length of that in *S*. *djadochtaensis* (∼11 cm in *ulanicus* versus ∼5.5 cm in *djadochtaensis*). The preorbital crest and orbitonasal sulcus that runs along the snout from the lacrimal to the naris are both weakly expressed in *S*. *djadochtaensis* (Fig. 3) relative to other putative *Shamosuchus* species. The splenial symphysis is long, extending to the level of the fourth maxillary tooth when in occlusion. Additionally, the splenials are V-shaped at the anterior terminus. These features in *S*. *djadochtaensis* differ from other putative *Shamosuchus*. Another diagnostic trait of *S*. *djadochtaensis* is that the fourth maxillary tooth is the largest in the tooth row. This condition is shared with the neosuchian *Bernissartia fagesii* but not other putative *Shamosuchus*, in which the fifth maxillary tooth is the largest. In other basal neosuchians (e.g., *Allodaposuchus*, Glen Rose Form, *Theriosuchus pusillus*), the third maxillary tooth is the largest.

### *Shamosuchus gradilifrons* (Konzhukova, 1954) [6]

Konzhukova [6] described this species from a nearly complete skull and partial postcranial material from the Upper Cretaceous Bayanshiree Fm. locality of Shireegin Gashoon, Mongolia. This material served as the type species of *Paralligator* before Efimov [10] synonymized *Paralligator* with *Shamosuchus*. Konzhukova [6] provides a brief but comprehensive description of *S*. *gradilifrons* including photographs of the original material and illustrations of the fully reconstructed skull.

**Fig 5:**
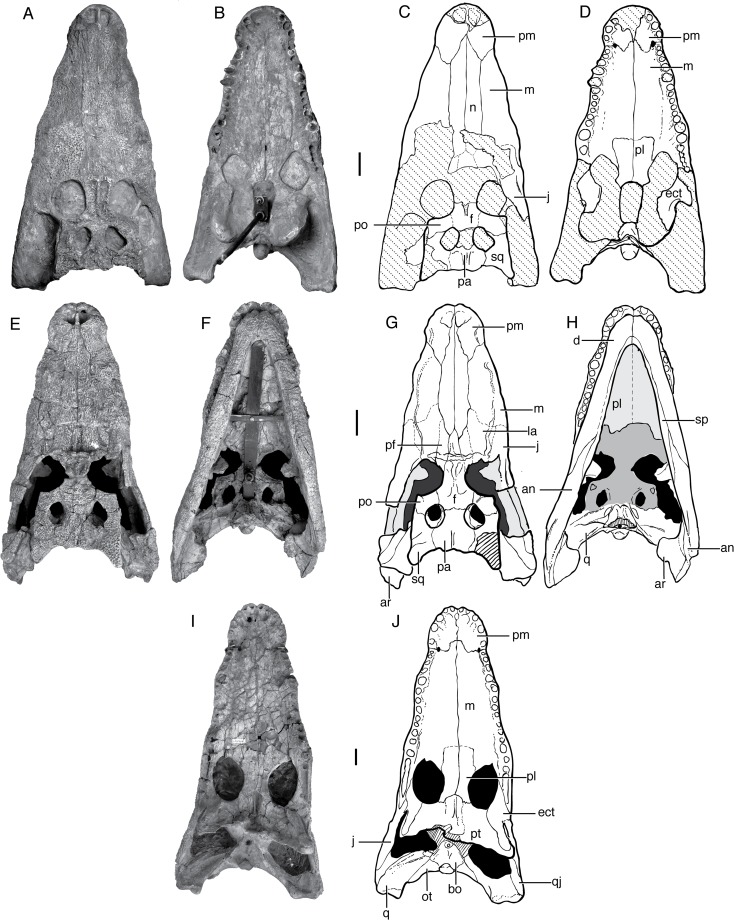
Paralligatorids from Bayanshiree Fm, Cenomanian to Santonian. **A**, *Shamosuchus* (= *Paralligator*) *major* (PIN 3726/501, holotype), dorsal view; **B**, PIN 3726/501, ventral view; **C**, line drawing of PIN 3726/501, dorsal view; **D**, line drawing of PIN 3726/501, ventral view; **E**, *Shamosuchus* (= *Paralligator*) *gradilifrons* (PIN 554-1, holotype), dorsal view; **F**, PIN 554-1, ventral view; **G**, line drawing of PIN 554-1, dorsal view; **H**, line drawing of PIN 554-1, ventral view; **I**, *Shamosuchus ulgicus* (= *Paralligator gradilifrons*) (PIN 3458/501), ventral view; **J**, line drawing of PIN 3458/501, ventral view. Abbreviations: an, angular; ar, articular; bo, basioccipital; d, dentary; ect, ectopteryoid; f, frontal; j, jugal; la, lacrimal; m, maxilla; n, nasal; ot, otoccipital; pa, parietal; pf, prefrontal; pl, palatine; pm, premaxilla; po, postorbital; pt, pterygoid; q, quadrate; qj, quadratojugal; sa, surangular; sp, splenial; sq, squamosal.

**Fig 6:**
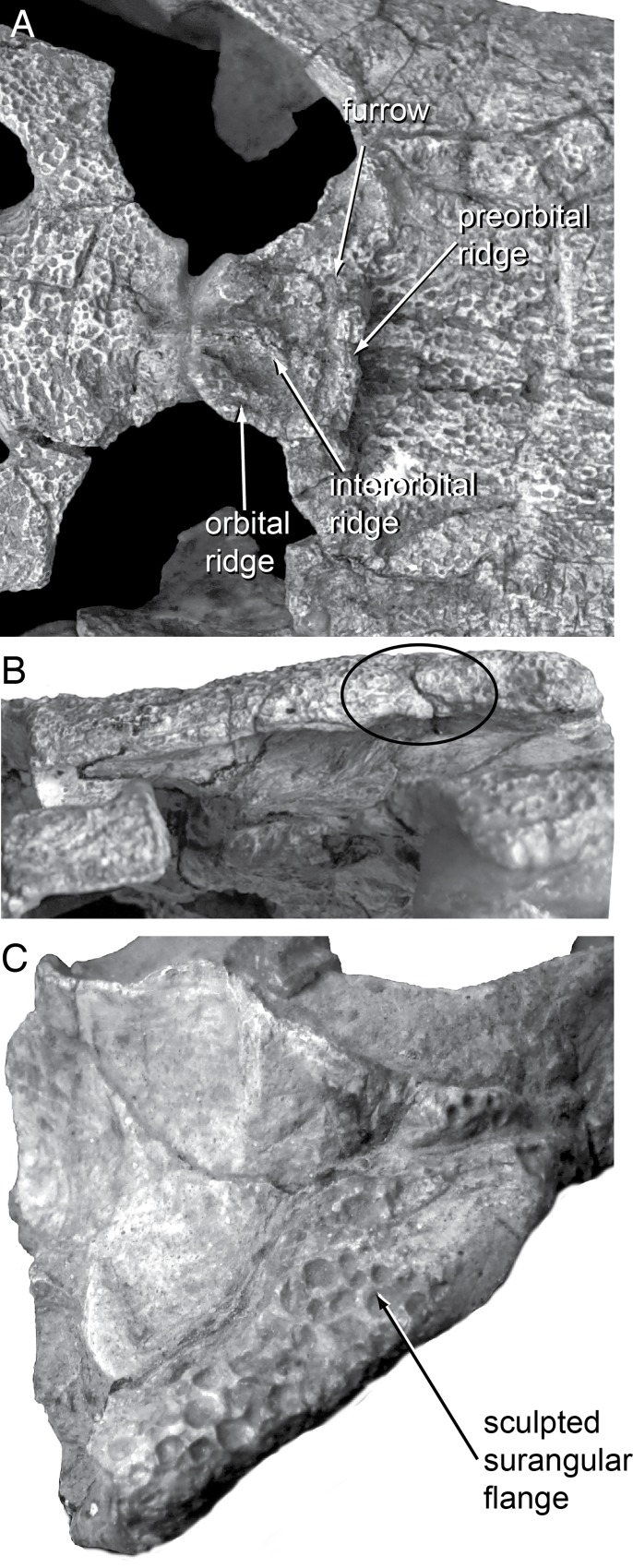
Skull morphology in the holotype of *Shamosuchus* (= *Paralligator*) *gradilifrons*. **A**, PIN 554-1, dorsal view of orbital region; **B**, PIN 554-1, left lateral view of squamosal; **C**, PIN 554-1, posterolateral view of right surangular.

The holotype skull (PIN 554-1; Figs. 5E-H and 6) is nearly complete but fails to preserve a few of important areas of the skull (most of the secondary palate including palatines and secondary choanae as well as the entirety of the pterygoids and ectopterygoids; note shaded regions in Fig. 5H). The absence of the choanae and pterygoids perhaps helped lead Konzhukova to the believe *Paralligator* was closely related to the *Alligator* radiation, given the otherwise superficial resemblance to *Alligator*. Presumably a more distant relationship to *Alligator* would have been espoused if the “mesosuchian” grade construction of the palate in *Paralligator* (like that known at the time in *Shamosuchus djadochtaensis*) had been known.

In addition to the damaged secondary palate and pterygoid wings, both postorbital bars are lacking and the right jugal is missing from roughly midorbit to its contact with the quadratojugal (note shaded regions in Fig. 5G). The frontal appears to have been broken mid-element and repaired leaving notches in the orbital margins. The left jugal was complete at the time of Konzhukova’s description but now is missing a small portion of the lower temporal bar. Most of the right squamosal is damaged (see Plate 1 in [6]) and is currently reconstructed with bone-colored plaster. The mandible is nearly complete except for the area surrounding the dentary-angular-surangular contact, which is now reconstructed with bone-colored plaster.

The diagnosis for *Paralligator* and for *S*. *gradilifrons* provided by Konzhukova [6] consists mostly of symplesiomorphies. She noted that *Paralligator* is characterized by a frontal that does not enter into the supratemporal fossae. However, in PIN 554-1 the frontals do enter the supratemporal fossae, but do not prevent contact of the parietal and the laterosphenoid within the fossa (Fig. 5E,G). As is common in many basal neosuchians (e.g., goniopholidids, *S*. *djadochtaensis*), the portion of the frontal within the supratemporal fossae bears a shallow depression. Furthermore, the presence of the frontal within the supratemporal fossa is widespread among other *Shamosuchus* species (*S*. *djadochtaensis*, *S*. *ancestralis*, *S*. *ulanicus*, and *S*. *ulgicus*).

Konzhukova [6] distinguished *S*. *gradilifrons* from other crocodyliforms on the basis of the dermal crests on the frontal and pre-orbital portion of the snout. She characterized the morphology as a frontal with a midline crest (interorbital crest) and two lateral crests (along the orbital margins) that connect to a transverse crest located just anterior to the orbital margin (pre-orbital crest) (Fig. 6A). Near the orbital margin, the prefrontals are raised in a wide flattened crest that continues inferiorly to the posterior margin of the lacrimal before turning and running anteriorly at the lacrimal-jugal contact. Konzhukova noted that all of these crests are flattened.

**Fig 7:**
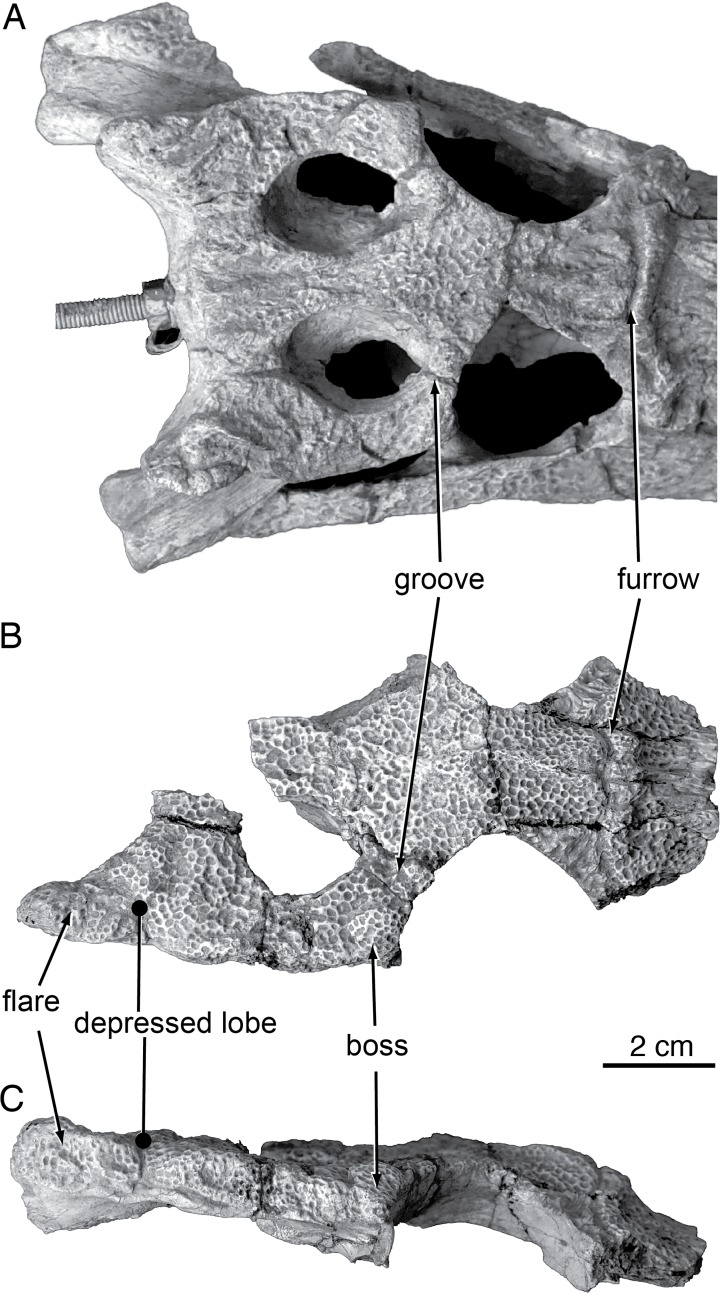
Skull morphology in Paralligatoridae. **A**, *Shamosuchus ulanicus* (= *Paralligator gradilifrons*) (PIN 3140-502), dorsal view; **B**, *S*. *ancestralis*, (= *Paralligator gradilifrons*) (PIN 551-29/1), dorsal view; **C**, *S*. *ancestralis*, (= *Paralligator gradilifrons*) (PIN 551-29/1), left lateral view.

While at the time this suite of morphologies distinguished *S*. *gradilifrons* from *S*. *djadochtaensis*, it did little to distinguish it from the much less complete *S*. *ancestralis*, which Konzhukova described in the same paper. In fact, a well-developed preorbital ridge (Figs. 6A and 7) appears widespread among *Shamosuchus* and closely related neosuchians (e.g., *Rugosuchus*, Glen Rose Form, *Wannchampsus*, *Theriosuchus*) as well as some goniopholidids. Indeed, pre-orbital ridges are also common among alligatoroids and a few crocodyloids. The “spectacle” of the spectacled caiman is the pre-orbital ridge. Likewise a midline frontal ridge appears more widespread among neosuchians.

**Fig 8:**
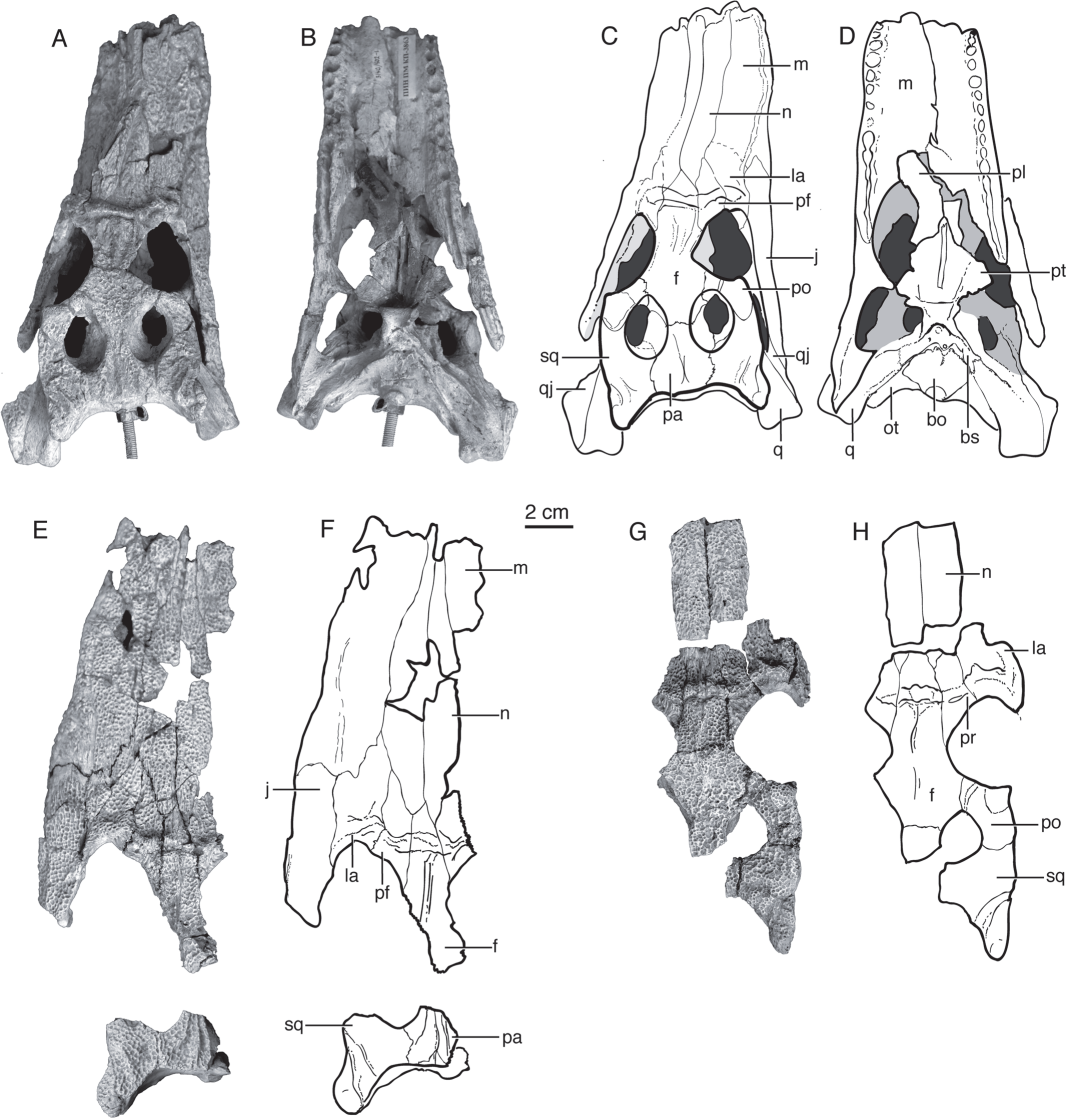
Paralligatorids from Nemegt Fm, Maastrichtian. **A**, *Shamosuchus ulanicus* (= *Paralligator gradilifrons*) (PIN 3140-502), dorsal view; **B**, PIN 3140-502, ventral view; **C**, line drawing of PIN 3140-502, dorsal view; **D**, line drawing of PIN 3140-502, ventral view; **E**, *S*. *tersus* (= *Paralligator gradilifrons*) (PIN 3141-501), dorsal view; **F**, line drawing of PIN 554-1, dorsal view; **G**, *S*. *ancestralis* (= *Paralligator gradilifrons*) (PIN 551-29/1), dorsal view; **H**, line drawing of PIN 551-29/1, dorsal view. Abbreviations: bo, basioccipital; bs, basisphenoid; d, dentary; f, frontal; j, jugal; la, lacrimal; m, maxilla; n, nasal; ot, otoccipital; pa, parietal; pf, prefrontal; pl, palatine; pm, premaxilla; po, postorbital; pt, pterygoid; q, quadrate; qj, quadratojugal; sq, squamosal.

Compared to all putative *Shamosuchus* now known, *S*. *gradilifrons* is characterized by a well-developed and heavily ornamented surangular flange on the retroarticular process (Fig. 6C). There appears to be a suite of features present in all or the majority of *Shamosuchus* taxa described from the Nemegt and Bayanshiree Fms. (Figs. 5 and 8). These include the fifth maxillary tooth being the largest (present in all *Shamosuchus* species except *S*. *djadochtaensis*), a groove that runs through the postorbital near the frontal-postorbital contact (present in all Nemegt and Bayanshiree taxa), a groove that runs through the preorbital crest (present in all Nemegt and Bayanshiree taxa), and a short mandibular symphysis relative to *S*. *djadochtaensis* (Figs. 3 and 5F, G). The majority of Nemegt and Bayanshiree *Shamosuchus* species have robust orbital crests on the frontal that parallel the midline interorbital crest (absent in *S*. *ancestralis* and *S*. *tersus*) (Figs. 5E, G and 6–8). The holotype of *S*. *gradilifrons* has a weakly expressed squamosal groove and posterior flaring of the squamosal (Figs. 6B and 7) combined with well-developed orbital ridges boarding a pronounced interorbital crest (Fig. 6A). This feature appears to be variably developed among the described *Shamosuchus* species.

### *Shamosuchus ancestralis* (Konzhukova, 1954) [6]

In the same publication that she described *Shamosuchus gradilifrons*, Konzhukova named a second species of “*Paralligator*”. The holotype of *Shamosuchus ancestralis* was collected from the younger Nemegt Fm. (Late Campanian/Early Maastrichtian) in the southern Gobi locality of Nemegt [6]. The holotype specimen (PIN 551-29/1) consists of scattered skull bones and a portion of a lower jaw (Figs. 7B, C and 8G, H). The holotype skull material consists of the proximal half of the nasals, the left and right prefrontal, most of the right lacrimal, the frontal, a partial parietal, the right postorbital and squamosal, and the right jugal. Konzhukova noted that the skull elements and dentary might have belonged to two individuals. Included in the paratype of *S*. *ancestralis* was a fragment of a left pterygoid (PIN 551-29/3), a right jugal (PIN 551-29/7), a mid-series dorsal vertebra (PIN 551-29/20), and a caudal vertebra (PIN 551-29/27).

**Fig 9:**
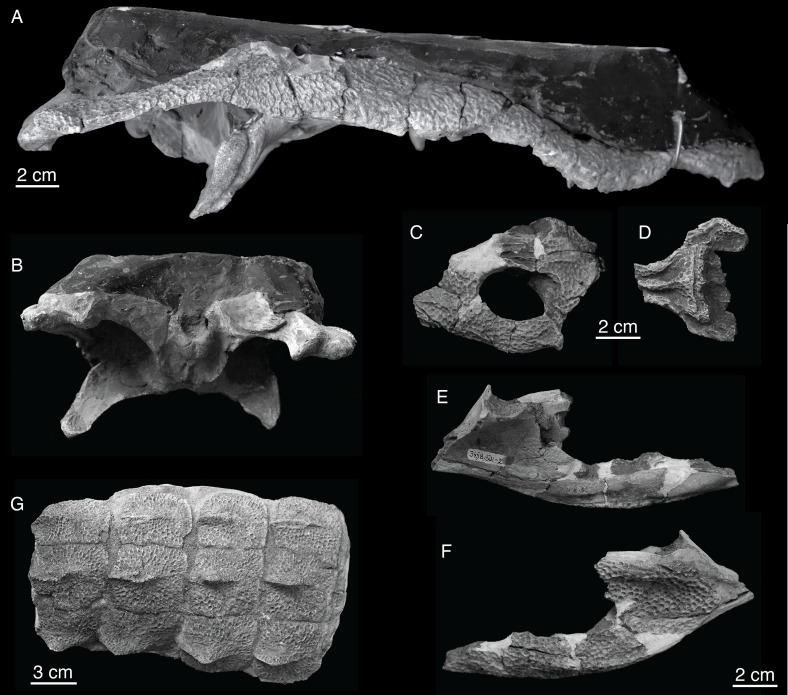
PIN 3458/501, *Shamosuchus ulgicus* (= *Paralligator gradilifrons*). **A**, skull in right lateral view; **B**, skull in posterior view; **C**, partial skull table dorsal view; **D**, frontal with partial prefrontals and lacrimals in dorsal view; **E**, posterior portion of mandible in medial view; **F**, posterior portion of mandible in lateral view; **G**, dorsal osteoderms in dorsal view.

As with *S*. *gradilifrons*, Konzhukova [6] distinguished *S*. *ancestralis* from other crocodyliforms mostly on the grounds of skull ornamentation. The interorbital crest on the frontal was described as short and gradually widening anteriorly. This seems little different from most other *Shamosuchus* species. Unlike *S*. *gradilifrons*, there are no lateral (orbital margin) crests in *S*. *ancestralis* (Fig. 8G, H). The preorbital crest possesses a deep furrow (Fig. 7B), but this too seems more widespread among *Shamosuchus* species (Figs. 7 and 8 and 9D). Konzhukova also noted that the postero-internal edge of the nasals is concave medially and that the back teeth on the lower jaw are blunted and clove shaped.

**Fig 10:**
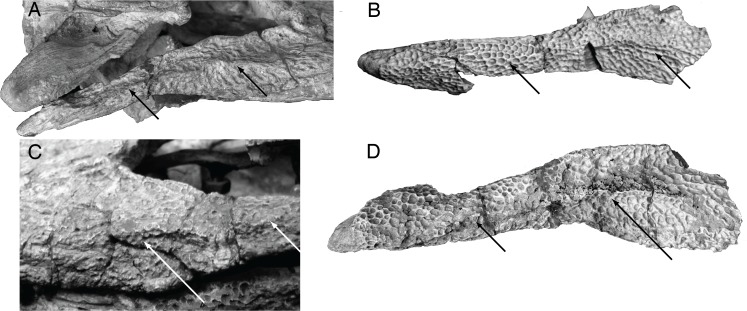
Jugal morphology in Paralligatoridae. **A**, *Shamosuchus ulanicus* (= *Paralligator gradilifrons*) (PIN 3140-502), right side; **B**, *S*. *tersus* (= *Paralligator gradilifrons*) (PIN 3141-501), right side; **C**, *S*. (= *Paralligator*) *gradilifrons* (PIN 554-1, holotype), left side; **D**, *S*. *ancestralis* (= *Paralligator gradilifrons*) (PIN 551-29/7), right side.

Although based on a relatively incomplete skull, PIN 551-29/1 preserves a number of features shared among *Shamosuchus* species. It possesses the preorbital crest (as noted by Konzhukova), a lateral ridge on the jugal (Fig. 10), and has the lobate squamosal with a depressed anterior margin and a flared posterior process seen in other *Shamosuchus* (Fig. 7B). There is a boss on the anterolateral corner of the postorbital and a narrow groove runs longitudinally across the dorsal surface of the postorbital near the postorbital-frontal contact (Fig. 7B). The groove through the postorbital is common to all *Shamosuchus* species except *S*. *djadochtaensis*. The boss is common to all *Shamosuchus* from the Nemegt Fm. (i.e., *S*. *ulanicus*, *S*. *tersus*), but it is weakly expressed in *S*. *tersus* and is variably present in the specimens assigned to *S*. *ulanicus*. Given the distribution of these features I see little basis to distinguish *S*. *ancestralis* from *S*. *gradilifrons* and thus consider *S*. *ancestralis* a subjective junior synonym of *S*. *gradilifrons*.

### *Shamosuchus sungaricus* (Sun, 1958) [9]

Sun [9] based this taxon on very incomplete remains from what is now identified as the Nenjiang Fm. of De-Hui (Tê-Hui) County, Jilin (Kirin) Province, on the Song-Liao (Sungarian) Plain of northeastern China (Li in [24]). The holotype and only known specimen consists of a few presacral vertebrae, dorsal osteoderms, a partial left femur, and the proximal part of a left tibia and fibula. These fossils are in poor condition and no characters present in the femur, tibia, fibula, or vertebrae are sufficient to refer to *Paralligator* (or *Shamosuchus*). Only the osteoderms show a potentially derived similarity with *Shamosuchus ancestralis* and *S*. *djadochtaensis* in being deeply pitted with a prominent posteriorly shifted sagittal keel.

This last feature at best serves to refer the De-Hui material to a larger *Shamosuchus*-containing clade. No features are present to diagnosis the species, and I view *Shamosuchus sungaricus* as a nomen dubium. One possibility is that the material belongs to the taxon *Rugosuchus nonganensis* which was described by Wu et al. [22] from much more complete remains discovered in correlative rocks only 44 kilometers southwest from the type locality of “*Paralligator sungaricus*”. However, as noted by Wu et al. [22], the paucity of “*P*. *sungaricus*” material makes referral to *Rugosuchus* problematic.

### *Shamosuchus borealis* (Efimov, 1975) [7]

Efimov [7] based a new taxon, *Kansajsuchus borealis*, on isolated incomplete remains from the Upper Cretaceous Bissekty Fm. of Dzharakhuduk, Uzbekistan. This Fm. is considered to be Turonian in age [25] and therefore is a partial temporal equivalent of the Bayanshiree Fm. of Mongolia. This was the second named species of *Kansajsuchus* with the type species *K*. *extensus* known from the Upper Cretaceous of Kansay, Tadzhikistan. At the time of their description both species of *Kansajsuchus* were thought to belong to the Goniopholididae. Recent work continues to place *K*. *extensus* in Goniopholididae based on phylogenetic analysis [26].

No formal diagnosis of the species was given; instead Efimov [7] provided a short description based on the holotype and additional referred material from the Dzharakhuduk and Shakh-Shakh sites. The holotype material (PIN 372/702, an isolated frontal and prefrontal) is insufficient to provide a diagnosis for the species. Although a median ridge on the frontal and a ridged prefrontal is similar in morphology to *Shamosuchus* spp., these features are present in other neosuchians (e.g., *Theriosuchus*). At best they bring one to the level of a larger *Shamosuchus* clade, but knowing what exact level requires an understanding of character distributions based on a phylogenetic analysis. I consider *Shamosuchus borealis* to be a nomen dubium with the holotype and referred material possibly referable to a more inclusive *Shamosuchus* containing clade.

### *Shamosuchus major* (Efimov, 1981) [8]

**Fig 11:**
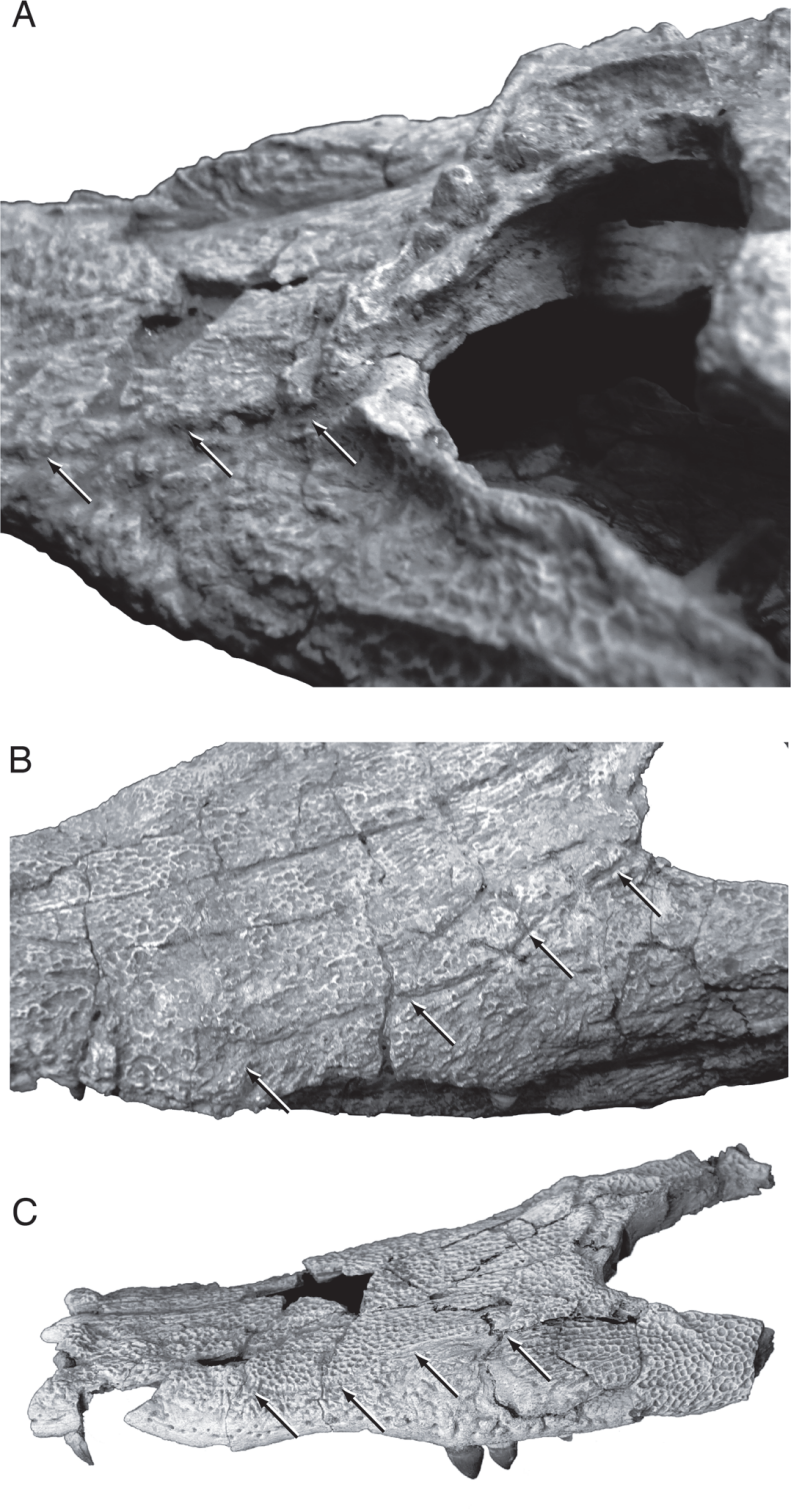
Orbitonasal sulcus variation in Paralligatoridae. **A**, *Shamosuchus ulanicus* (= *Paralligator gradilifrons*) (PIN 3140-502), left side; **B**, *S*. (= *Paralligator*) *gradilifrons* (PIN 554-1, holotype), left side; **C**, *S*. *tersus* (= *Paralligator gradilifrons*) (PIN 3141-501), left side.

*Shamosuchus major* was described on the basis of a large, incompletely preserved skull and isolated skull remains from two other individuals. PIN 3726/501 (the holotype) and PIN 3726/502 were collected in 1974 at Khongil Tsav from the Bayanshiree Fm. (Fig. 5A–D). Efimov [8] provided a brief description of *S*. *major* citing the poor preservation and incompleteness of the holotype. He noted that it differed from all other species of *Paralligator* (as it was named at the time) by its large size and the medial contact of the premaxillae posterior to the external naris. Efimov provided no explicit justification for inclusion of these fossils in *Paralligator*, although the introduction to the paper provides some insight. Here Efimov mentions a set of retained primitive and derived features present in *Paralligator*. Included in this list is contact between the lacrimal and nasal, frontal participation in the supratemporal fenestra, a greatly elongated secondary choana, and presence of “lateral line” troughs on the skull (what I describe here as an orbitonasal sulcus) (Fig. 11). Efimov specifically mentions these traits to cast doubt on the presumed close affinity between *Paralligator* and *Alligator*, but they could easily have been informing his inclusion of other material into *Paralligator*.

*Shamosuchus major* is a large animal (60 cm skull length), which is even more striking given the size of most other species of *Shamosuchus* are considerably smaller (e.g., PIN 3141/501: ∼24 cm; PIN 3140/502-1: ∼20.5 cm; IGM 100/1195: ∼11.5 cm). It is unclear on what basis the referred specimen (PIN 3726/502) was included in the taxon. PIN 3726/502 is a partial palatal ramus of a right maxilla and a fragment of a parietal that do not seem to be from the same sized animal. I have chosen to only consider the holotype material in assessing *S*. *major*.

PIN 3726/501 is a heavily reconstructed skull (Fig. 5A–D). In dorsal view I interpret both the left and right quadrates and quadratojugals to be completely reconstructed. On the left side, the jugal, most or all of the lacrimal and prefrontal, including part of the posterior ramus of the maxilla, and the posterolateral corner of the squamosal are reconstructed. On the right side, the posterior process and ascending process of the jugal are reconstructed, as are parts of the lacrimal and prefrontal. The orbits, the right infratemporal fenestra, and most of the right infratemporal fenestra are reconstructed. Along the midline nearly all of the frontal has been reconstructed. Anteriorly, the snout has been reconstructed such that none of the actual naris remains.

A similar situation is present on the ventral surface of the skull. Both quadrates and infratemporal regions have been reconstructed. Most of the posterior aspect of the braincase appears to be genuine. The pterygoid flanges and choanae are reconstructed, as are the suborbital fenestrae. Both ectopterygoids appear to be real. The tip of the snout is less reconstructed in ventral aspect than in dorsal aspect (Fig. 5C, D). Most of the reconstruction is restricted to the right premaxilla. The left premaxilla is only partially reconstructed around the level of the first alveolus. On the right side, the reconstruction stretches to the fourth alveolus and no sign of an incisive foramen is present. Many of the large maxillary teeth are reconstructed as well.

Other than its massive size little distinguishes *Shamosuchus major* from the other *Shamosuchus* species, especially the contemporaneous forms from the Bayanshiree Fm. (i.e., *S*. *ulgicus* and *S*. *gradilifrons*). The lobate squamosal with a depressed anterior margin is only weakly expressed in *S*. *major*, but this does not differ greatly from the weak expression of the same trait in the *S*. *gradilifrons* holotype. Likewise, all Bayanshiree *Shamosuchus* species (in fact all species currently referred to *Shamosuchus*) have a lateral ridge on the anterior process of the jugal (Fig. 10). The reduced or weak expression of these features in *Shamosuchus major* may be the result of ontogenetic differences given the large size of *S*. *major*. A single autapomorphy exists in *S*. *major* and was highlighted by Efimov [8], which is the contact of the premaxillae posterior to the nares. This is a derived trait among crocodyliforms, but the palate is unquestionably that of a more basal neosuchian. Although incompletely preserved, the secondary choanae was not pterygoid-bound as evinced by the smooth posterior borders of the palatines.

### *Shamosuchus ulgicus* (Efimov, 1981) [8]

Efimov provided a short description of this species based on the holotype and only known specimen consisting of an incomplete skull and partially articulated set of osteoderms (PIN 3458/501; Fig. 9). The species name “*ulgicus*” is derived from the name of the ancient monastery near Ulgii Amtgai where the specimen was found. Like the geographically proximate *Shamosuchus major*, *S*. *ulgicus* is from the Upper Cretaceous Bayanshiree Fm.

The palate is very well preserved in PIN 3458/501; however the dorsal surface of the skull is encased in a block of plaster painted black (Fig. 5I, J). It is unclear if the skull had been prepared as such at the time of Efimov’s description. Fragments of the skull table and the interorbital region remain free of the plaster (Fig. 9). The ventrolateral surface of the rostrum is exposed, as is the posterior portion of the skull up to the dorsoventral height of the jugals. The occiput is exposed up to a level just dorsal to the foramen magnum.

As Efimov noted, the interorbital region of the frontal is narrow. The orbital margins have well-developed ridges separated from the large midline crest by deep sulci (Fig. 9D). This interorbital crest is continuous with robust preorbital ridge common to *Shamosuchus* species. As in all *Shamosuchus* species except *S*. *djadochtaensis*, the preorbital ridge possesses a deep midline groove that divides the ridge into an anterior and posterior half (Fig. 9D). As in *S*. *ancestralis* and *S*. *gradilifrons* the preorbital groove ends at the orbital margins.

Efimov [8] noted the presence of lateral line-like grooves (like those he identified in *S*. *major*) on the dorsal surface of the snout in *S*. *ulgicus*. He described them as deep canals that trace from the outer nostril to the orbit and suggests that the left and right “lateral lines” anastomose by way of the preorbital groove. The extent of these orbitonasal grooves can no longer be confirmed because of the plaster encasing the dorsal surface of the skull but a portion of the orbitonasal groove is evident on the exposed maxilla and jugal on the right side of the holotype (Fig. 9).

The interorbital crest is not expressed at the level of the postorbital process of the frontal. The frontal contacts the parietal in a relatively straight interdigitating suture, roughly one-third the way posteriorly through the interfenestral bar on the skull table. The contact between the frontal, parietal, and postorbital is visible within the supratemporal fossa. The dorsal surface of the postorbital is heavily sculpted, as is the rest of the skull table. Lateral to the suture with the frontal, the postorbital possesses a shallow but distinct anteroposteriorly-running groove transecting the bone (Fig. 9C). A similar groove is evident in *S*. *ancestralis* and is weakly expressed in the *S*. *gradilifrons* holotype (Fig. 7). The right squamosal displays the shallow and broad groove described above for *S*. *djadochtaensis*. In *S*. *ulgicus* the groove is much shallower, such that the “flared” appearance to the lateral profile of the squamosal is more subdued in *S*. *ulgicus* compared to *S*. *djadochtaensis* or *S*. *ulanicus*.

The palate of *S*. *ulgicus* is highly fractured but preserved in place making it one of the more complete palates known for *Shamosuchus* (Fig. 5I, J). Efimov [8] noted a few features visible in palatal view. The premaxilla bears five alveoli and the maxilla has 19, with the 14th through 19th merged into a single dental channel. The incisive foramen is situated just posterior to the first premaxillary alveoli with the anteriormost extent of the foramen just entering the gap between the alveoli. The secondary choanae are divided by a septum and shifted far posteriorly as in most derived neosuchians, but they are not completely bound by the pterygoids. The choanal groove is very narrow relative to the width of the pterygoid plate and the interfenestral bar. The palatines form the anterior margin to the choanae, but the posteriormost portions of the palatines do not reach the posterior margin of the suborbital fenestrae.

**Fig 12:**
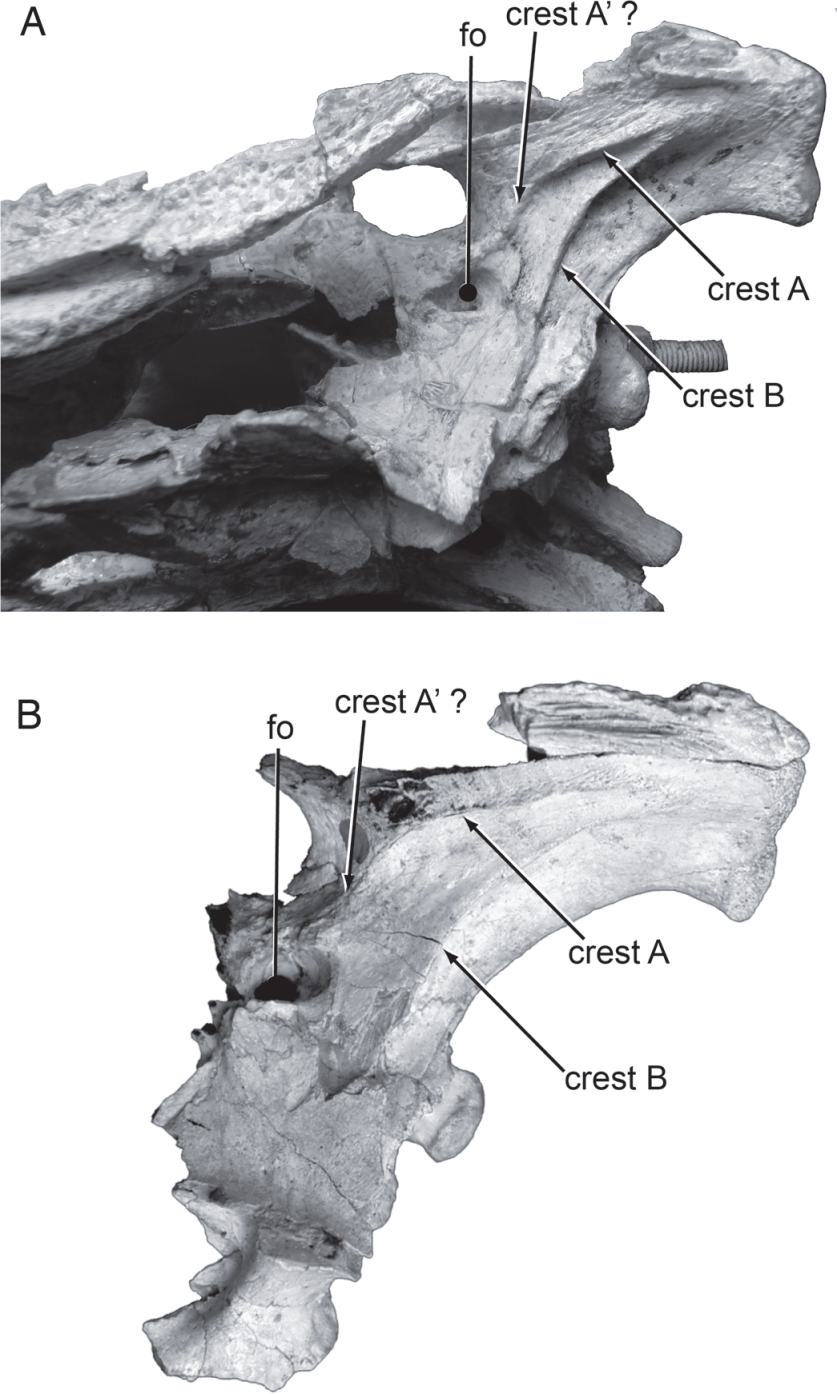
Lateral braincase morphology in Paralligatoridae. **A**, *Shamosuchus ulanicus* (= *Paralligator gradilifrons*) (PIN 3140-502), left side; **B**, *S*. *tersus* (= *Paralligator gradilifrons*) (PIN 3141-501), left side. Abbreviations: fo, foramen ovale.

On the ventral surface of the quadrate, Efimov [8] noted “a peculiar system of the sharp ridges (A’) for the rear jaw adductors” (Fig. 12). *S*. *ulgicus* does have a well-developed crest A and B (*sensu* [23]), but the presence or absence of a crest A’ can’t be confirmed because of the plaster infilling the adductor chamber.

A small segment of the dorsal dermal shield is present in PIN 3458/501 (Fig. 9G). The most complete portion consists of 12 osteoderms preserved in articulation. There is no indication of imbrication between the osteoderms and the anterior margins lack smooth articular surfaces. The three preserved rows appear sutured together mediolaterally. Examination of the free margins of the outer rows of osteoderms suggest that what is preserved in PIN 3458/501 are the two paramedian rows of osteoderms and the left lateral row of osteoderms. All the preserved osteoderms bear large midline keels. As in *S*. *djadochtaensis* [5], the keels are asymmetrically developed and displaced to the posterior margin of each osteoderm. In PIN 3458/501 the keels on the paramedian osteoderms are larger than those in the lateral row.

The alveolar ramus of the maxilla in *S*. *ulgicus* is more sinusoidal in lateral view (i.e., vertically festooned) than most other putative *Shamosuchus* species, but variation is only minor (Fig. 9A). The fracturing of the holotype may have exaggerated the lateral contour of the snout. Contrary to Efimov [8], the pre- and interorbital crests of *S*. *ulgicus* are quite similar to *S*. *gradilifrons* and *S*. *ancestralis*. It is true, however, that the snout is proportionally narrower in *S*. *ulgicus* than in *S*. *gradilifrons*. Comparisons in snout proportions to *S*. *major* are more difficult to make because the larger absolute size difference (and thus possible ontogeny scaling) between the two species. Very little distinguishes *S*. *ulgicus* from the other Bayanshiree Fm. *Shamosuchus* species (*S*. *gradilifrons* and *S*. *major*) or from the Nemegt Fm. species. I find that the wealth of character data discussed above indicates that PIN 3458/501 belongs to *Shamosuchus* and that it should be considered a junior synonym of *S*. *gradilifrons*.

### *Shamosuchus occidentalis* Efimov, 1982 [10]

*Shamosuchus occidentalis* was named by Efimov [10] from a fragment of a skull (PIN 327/721) from the Upper Cretaceous Bissekty Fm. of Dzharakhuduk, Uzbekistan. In the same publication Efimov synonymized *Paralligator* with *Shamosuchus*. Both Efimov [13] and Nesov [27] considered *Shamosuchus occidentalis* [10] to be a junior synonym of *Shamosuchus borealis*. *Shamosuchus occidentalis* is known from the same locality as the referred material of *S*. *borealis*. The 5th maxillary tooth is the largest in *S*. *borealis* but the 6th is the largest in *S*. *occidentalis*, so it is unclear what the basis of this conclusion was other than the specimens being from the same locality in Uzbekistan. The skull of *S*. *occidentalis* consists of a partial snout including most of the left and right maxillae and the nasals. The snout lacks the premaxillary portion and is broken well before the orbits. The surface of the snout is heavily eroded so little can be said of the bone texture. The apparent absence of an antorbital fenestra, double wave of maxillary tooth enlargement, and the relatively dorsoventrally compressed snout suggests neosuchian affinities. The sixth maxillary alveolus is the largest, unlike all other *Shamosuchus* species wherein either the fourth (*S*. *djadochtaensis*) or the fifth (*S*. *gradilifrons*) is the largest. No other trait is present in PIN 327/721 to warrant a referral to *Shamosuchus*. The overall lack of diagnostic characters beyond Neosuchia leads me to consider this taxon a nomen dubium.

### *Shamosuchus ulanicus* Efimov, 1983 [11]

*Shamosuchus ulanicus* was described from the Nemegt Fm. locality of Ulaan Bulag by Efimov [11] on the basis of a nearly complete skull (PIN 3140-502; Fig. 8A–D). Efimov stated that this skull differed from other *Shamosuchus* in the sharpness and fullness of the crests on the skull roof, longitudinally elongated supratemporal fenestra, and the 11th maxillary tooth being larger than the 12th maxillary tooth.

I find no basis to distinguish the preorbital and interorbital crests in PIN 3140-502 from those of other Nemegt Fm. *Shamosuchus* material and the supratemporal fenestra is not appreciably more elongate in PIN 3140-502 than in *S*. *ancestralis* or PIN 3141-501. On the ventral surface of the quadrate of *S*. *ulanicus*, crest A [23] is well developed and extends above the opening for the trigeminal nerve (Fig. 10B). This elongate crest A may in fact be a fusion of crest A and A’ given that when A’ is present in extant taxa it is located near the trigeminal foramen. This feature is also present in *Shamosuchus ulgicus* from the Bayanshiree Fm. The 11th maxillary tooth is indeed the largest posterior maxillary tooth (based on alveolus size) unlike PIN 3141-501 (*S*. *tersus*), *S*. *gradilifrons*, PIN 3458/501 (*S*. *ulgicus*), and *S*. *major* where the 12th maxillary tooth is the largest. I find this to be an insufficient basis to erect a new taxon and therefore consider *Shamosuchus ulanicus* as a junior synonym of *S*. *gradilifrons*.

### *Shamosuchus tersus* Efimov, 1983 [11]

**Fig 13:**
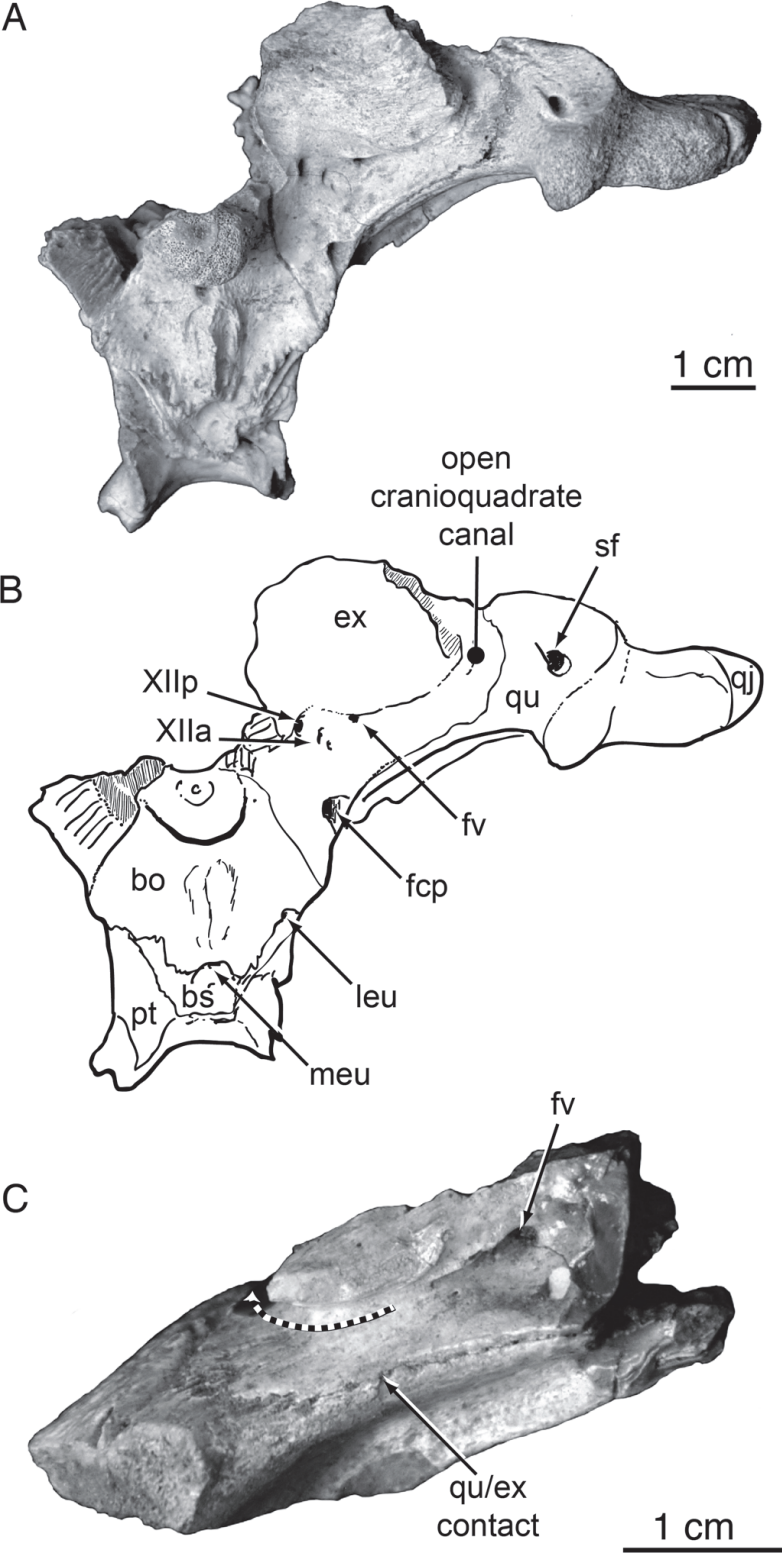
Braincase and suspensorium morphology in Paralligatoridae. **A**, *Shamosuchus tersus* (= *Paralligator gradilifrons*) (PIN 3141-501), occipital view; **B**, *S*. *tersus* (= *Paralligator gradilifrons*) (PIN 3141-501), line interpretation; **C**, *S*. *tersus* (= *Paralligator gradilifrons*) (PIN 3141-501), left quadrate and exoccipital. In **C**, dashed line marks path of open cranioquadrate canal. Abbreviations: bo, basioccipital; bs, basisphenoid; ex, exoccipital; fcp, posterior carotid foramen; fv, foramen ovale; leu, lateral Eustachian foramen; meu, median Eustachian foramen; pt, pterygoid; qj, quadratojugal; qu, quadrate; sf, siphoneal foramen; XIIa, foramen for anterior branch of hypoglossal nerve; XXIp, foramen for posterior branch of hypoglossal nerve.

Efimov [11] described the third *Shamosuchus* species from the Nemegt Fm. from the Nogoon Tsav locality. *Shamosuchus tersus* is based on a partial skull (PIN 3141-501) that includes most of the snout and a partial but very well preserved braincase (Figs. 8E, F and 12B and 13). Some isolated osteoderms were also referred to this taxon. Efimov provided little morphological diagnosis, citing a more anteriorly extended interorbital crest and the round posterior shape of the choanae. I find no appreciably difference in these features with other putative *Shamosuchus* specimens and therefore consider *Shamosuchus tersus* as a junior synonym of *S*. *gradilifrons*.

### *Shamosuchus karakalpakensis* (Nesov et al., 1989) [12]

Nesov et al. [12] based this species on an isolated frontal collected from the Cenomanian Khodzhakul Fm. of Uzbekistan. The holotype provides no viable diagnostic features for the species and can at best be referred to Neosuchia. I consider *Shamosuchus karakalpakensis* to be a nomen dubium.

### Putative Close Relatives of *Shamosuchus*

#### Rugosuchus nonganensis

Wu et al. [22] described this taxon from Lower Cretaceous rocks of the Nenjiang Fm. of Nong’an county China on the basis of a nearly complete skull (IGV 33). The authors referred two additional specimens to the taxon: IGV 31 consisting of vertebrae, dorsal and ventral osteoderms, an incomplete pelvic girdle, and limb fragments; and IGV 32 consisting of two sacral vertebrae, a caudal vertebra, left ilium with the head of the left femur, and some dorsal osteoderms. Rocks in which this taxon was discovered are only 44 kilometers from, and correlative with, the holotype locality of “*Paralligator sungaricus*”.

Only the skull of has been described and it is at least superficially distinct from species of *Shamosuchus*. The snout of *Rugosuchus* is long, especially when compared to the relatively short-snouted *S*. *djadochtaensis*. *Rugosuchus* appears to have a notch at the premaxilla/maxilla contact, but as noted by Wu et al. [22] this may be exaggerated due to damage. There is a series of depressions along the dorsal surface of the maxilla, aligned between the maxillary alveoli that are absent in species of *Shamosuchus*. These differences notwithstanding, *Rugosuchus* exhibits a suite of derived features that united it with *Shamosuchus djadochtaensis* in the phylogeny of Pol et al. [5]. These include a sagittal ridge on the dorsal surface of the frontal, a unified opening for the exit of cranial nerves IX-XI, the posterior region of the palatine bar between the suborbital fenestra is flared posteriorly, and the lateral surface of the angular bears a distinct longitudinal ridge. Several other features present or potentially present in *Rugosuchus* and common to the *Shamosuchus* species reviewed here may serve to strengthen the relationship between the two taxa.

#### Glen Rose Form

The so-called Glen Rose Form is an undescribed taxon originally illustrated and then mentioned by Langston [28, 29] as a putative close eusuchian relative. The Glen Rose Form has been included in numerous phylogenetic analyses and has consistently been recovered near the base of Eusuchia [5,20,21,30,31,32,33]. Its potential importance as a “transitional” taxon was based on the presence of an intermediate choanal morphology between more plesiomorphic mesoeucrocodylians and true eusuchians, and the procoelous condition of the referred vertebrae.

Which specimens are considered to constitute the Glen Rose Form has had a problematic history. Crocodyliform elements recovered from the Glen Rose Fm. as well as other Trinity Group Fms. have been variously included into this informal taxon [33]. Two skulls have been linked to it (USNM 22039 and MCZ 4453), as has postcranial remains (e.g., TMM 40595, TMM 41306, TMM 41307, TMM 42995-2, TMM 40644-1). Rogers [34] made the first inroads into clarifying the jumble of morphology present in the Glen Rose Form. Based on crocodyliform material recovered from a single Trinity Group locality (SMU 331), Rogers erected a new taxon, *Pachycheilosuchus trinquei*, that given its morphology suggested the isolated vertebrae and associated elements (TMM 40595, TMM 41306, TMM 41307) are referable to *Pachycheilosuchus*, not the Glen Rose Form taxon.

This result prompted Pol et al. [5] to take a conservative approach in the phylogenetic analysis of their study of *Shamosuchus djadochtaensis* and score the Glen Rose Form based only on the skull material (USNM 22039 and MCZ 4453). This resulted in a more basal position among neosuchians than in previous analyses including the Glen Rose Form, likely due to the lack of procoelous vertebral features pulling the taxon crownward. The Glen Rose Form occupied a position more derived than Atoposauridae but was one node down-tree from the *Shamosuchus* + *Rugosuchus* clade. Pol et al. [5] provide a detailed discussion of the character support and character conflict associated with the position of the Glen Rose Form among plesiomorphic neosuchians, therefore I will not revisit that discussion here.

In the present analysis I have taken an even more conservative approach. I score the Glen Rose Form based solely on the USNM 22039 skull, which is from the Trinity Group’s lateral equivalent the Antlers Fm. of north Texas. I have chosen to exclude MCZ 4453 (recovered from the Antlers-equivalent Cloverly Fm. of Wyoming) as I await detailed descriptive work on the Glen Rose Form and its associated material, and assessment whether USNM 22039 and MCZ 4453 are in fact from the same species. This approach mirrors the scoring of the recent papers by Adams [33,35]. Adams recovers the Glen Rose Form as the sister taxon to a new species from the Twin Mountains Fm. near Procter Lake, Texas. These two species are deeply nested within the *Shamosuchus* + *Rugosuchus* clade—a novel position for the Glen Rose Form and one I seek to directly test here with added data on the various *Shamosuchus* species.

#### Batrachomimus pastosbonensis

Montefeltro et al. [36] recently described a neosuchian from the Late Jurassic of northeastern Brazil that, based on the authors’ phylogenetic analysis, was the sister taxon to *Shamosuchus* + *Rugosuchus*. The recovery of an apparent paralligatorid in Late Jurassic deposits expands the temporal range of the clade by 30 million years and provides a critical data point for understanding the biogeographic history of neosuchians. *Batrachomimus* shares apomorphic features with *Shamosuchus djadochtaensis*. These include a shallow hemispherical depression bordered by an elevated rim on the lacrimal and prefrontal, as well as palatines that flare at both their anterior and posterior ends [36]. The jugal possesses a long anteroposterior running ridge, bordered dorsally by a shallow depression under the orbit similar to the one seen in *S*. *djadochtaensis* [5]. It also has an open cranioquadrate canal. *Batrachomimus* differs from *Shamosuchus* species by lacking a midline frontal ridge or preorbital ridges. Moreover, there appears to be no orbitonasal sulcus on the maxilla. The palate of *Batrachomimus* is similar to more basal mesoeucrocodylians in a number of aspects. The choana is anteriorly placed between the suborbital fenestrae, the choanal groove is shallow, and the choanal septum is long, robusts and flatted along its ventral edge. Fully understanding the phylogenetic placement of *Batrachomimus* is hindered by the absence of most of the skull table. As discussed above, numerous *Shamosuchus* and putative paralligatorid synapomorphies pertain to the morphology of the postorbital and squamosal.

#### Wannchampsus kirpachi

Adams [33] described this taxon from the Lower Cretaceous Twin Mountains Fm. of north-central Texas. This Fm. is part of the Trinity Group and is overlain by the Glen Rose Fm. Thus it is a lateral equivalent of the Antlers Fm. from where the Glen Rose Form is known (see above). This taxon is known from two partial skulls, each of which lacks large portions of the rostrum. Phylogenetic analysis recovered *Wannchampsus* as the sister taxon to the Glen Rose Form, which were together nested with *Shamosuchus djadochtaensis* and *Rugosuchus*. Adams [33] suggested that the Glen Rose Form might be referable to *Wannchampsus*.

The supratemporal fossa extends far anteriorly onto the frontal and there is a sulcus that connects the supratemporal fossa to the occipital margin as in *Theriosuchus pusillus*. The medial wall of this sulcus is better developed than the lateral wall. It is formed by the parietal and is continuous with the ridge bounding the supratemporal fenestra. As in *Theriosuchus*, *Shamosuchus*, and *Rugosuchus* the squamosal has an unsculptured or weakly sculptured posterolateral lobe. A sharp ridge delimits the beginning of the lobe. The unsculptured area leads anteriorly into the groove on the lateral edge of the squamosal for the external earflap musculature. The frontal and parietal in *Wannchampsus* bear midline ridges (as in *Theriosuchus*, *Shamosuchus*, and *Rugosuchus*) and the secondary choana is located posteriorly on the secondary palate but is not pterygoid bound.

#### Theriosuchus

Owen [37] described *Theriosuchus pusillus* from two specimens from the Early Cretaceous Purbeck Limestone of England. *Theriosuchus* is a relatively small crocodyliform with a brevirostrine skull that lacks an antorbital fenestra. Since Joffe [39], *Theriosuchus* is typically considered to be an atoposaurid along with *Alligatorellus*, *Alligatorium*, *Atoposaurus*, *Montsecosuchus*, and *Brillanceausuchus* [20,40,41,42].

Phylogenetic analyses of Neosuchia have thus far typically included only *Theriosuchus pusillus* and perhaps *Alligatorium* as representatives of Atoposauridae (e.g., [5,43,44,45,46] but see [47,48]). *Theriosuchus* remains have been reported from throughout Europe [48] and less complete remains from Thailand [49] and Inner Mongolia [50] have been referred to *Theriosuchus*. Much of the *Theriosuchus* record is from Late Jurassic or Early Cretaceous sediment but the recently described *Theriosuchus sympiestodon* [47,51] greatly extends the range of *Theriosuchus* into the Late Cretaceous, indicating that the taxon is very long-lived with high levels of morphological conservation. There are five named species: *T*. *pusillus*, *T*. *ibericus*, *T*. *guimarotae*, *T*. *grandinaris*, and *T*. *sympiestodon*. Of these, only *T*. *pusillus*, *T*. *guimarotae*, and *T*. *sympiestodon* have been included in phylogenetic analyses [47], and validity of *T*. *ibericus* has been called into question [48].

*Theriosuchus* has a number of derived features including a posteriorly placed choanae, extensive contact of the palatines enclosing the floor of the nasopharyngeal passage, no antorbital fenestra (possibly present in *T*. *guimarotae*, *T*. *sympiestodon*, *T*. *ibericus*), and at least some procoelous vertebrae. Thus *Theriosuchus* has long been appreciated as a neosuchian and one of the possible sister groups to Eusuchia [17,20,37,38]. Cladistic analyses have universally supported the neosuchian affinities of *Theriosuchus* although the exact position has varied. As taxonomic sampling of advanced neosuchians has increased (e.g., [5]), the number of neosuchians separating *Theriosuchus* and other atoposaurids from the crown has increased. Most analyses including *Theriosuchus* differ in its placement by one node. Some recover *Theriosuchus* as the second most basal divergence in Neosuchia, being closer to Eusuchia than are thalattosuchians (e.g., [5,22,45,52]). Other analyses recover it at the base of Neosuchia with thalattosuchians closer to Eusuchia than *Theriosuchus* (e.g., [34,35,47]). The analysis of Jouve [46] found Atoposauridae in one of the most derived positions as the sister-taxon to the clade containing *Bernissartia* + *Borealosuchus* + Crocodylia.

Diagnoses for *Theriosuchus* have varied since the initial description of *T*. *pusillus*, with an increasing focus on apomorphies. Clark [20] identified as diagnostic a midline ridge on the dorsal surface of the parietal and frontal, transverse contact between the lacrimal and the nasal, and a frontal that extend posteriorly well beyond the anterior limits of the supratemporal fenestra. Schwarz and Salisbury [48] added a list of plesiomorphic and apomorphic features to the diagnosis. One of the more interesting apomorphic features added by Schwarz and Salisbury [48] was the presence of a shallow sulcus on the dorsal surface of the maxillary rostrum, immediately caudal to the junction between the maxilla, premaxilla and nasal. This feature was similarly noted by Martin et al. [47] and characterized as a transversely directed groove on the anterolateral side of the maxilla. This feature had not been previously noted in *T*. *pusillus* although examination of NHMUK 48330 confirms its presence in that taxon. It is also present in *T*. *ibericus* [54]. Also present in *Theriosuchus pusillus* (and apparently most other *Theriosuchus* species) is a small unsculptured lobe of bone that lies at the posterior end of the lateral edge of the squamosal. Noted by Clark [20] as a synapomorphy of Atoposauridae, this feature is also reported present in *Alligatorium meyeri*.

I am unaware of any prior comparisons between *Theriosuchus* and *Shamosuchus* exploring a possible close relationship. There are, however, a number of features that are similar. The cranioquadrate canal is open in *Shamosuchus* (AMNH FARB 6412, IGM 100/1195, PIN 3141/501) contra the observation of Pol et al. [5] (Fig. 13). Although not preserved in the lectotype of *Theriosuchus pusillus*, it appears to be open in both *T*. *sympiestodon* and *T*. *guimarotae* taking the form of a sulcus that is roofed by the squamosal along its path on the quadrate. The maxillae in *Shamosuchus* and *Theriosuchus* bear a groove that begins near the orbital margin and terminates near the external naris. The frontal and parietal in both taxa have prominent midline ridges. *Theriosuchus* has a distinct, depressed, and smooth posterolateral “lobe” on the squamosal. This is similar to the posterolateral squamosal “lobe” present in *Shamosuchus* [5] and *Rugosuchus* [22]. In the latter taxa there is some presence of sculpturing on the lobe. However, as noted by Clark [20], sculpturing extends somewhat onto the lobe in *Theriosuchus pusillus* (NHMUK 48216). These comparisons require testing within a phylogenetic analysis. Additionally, these features could be shared because the traits are more widespread among basal neosuchians than previously appreciated.

## Phylogenetic systematics of “*Shamosuchus*”

### Taxon Sampling

The complete dataset included 101 crocodylomorph taxa plus the outgroup (*Gracilisuchus stipanicicorum*) used to root the phylogenetic trees. The taxon *Candidodon itapecurense* was excluded from the searches. The holotype of this taxon is extremely fragmentary and can be resolved nearly anywhere on the crocodyliform tree. This uncertainty is produced by the large amount of missing data rather than by character conflict (scorings on the type specimen have 98% data missing). The sampling scheme follows that of [52] and [53] with the inclusion of fourteen additional neosuchian taxa. Details of the character set are in Appendix II in S1 Document. Reference specimens and literature consulted for information on the ingroup taxa are available in Appendix I in S1 Document. and the full dataset is available on MorphoBank [55] at http://www.morphobank.org/permalink/?P1200.

### Tree Search Strategy and Node Support

The phylogenetic dataset was analyzed with equally weighted parsimony using TNT v. 1.0 [56,57]. A heuristic tree search strategy was conducted performing 10,000 replicates of Wagner trees (using random addition sequences, RAS) followed by TBR branch swapping (holding 10 trees per replicate). The best trees obtained at the end of the replicates were subjected to a final round of TBR branch swapping. Zero-length branches were collapsed if they lacked support under any of the most parsimonious reconstructions (i.e., rule 1 of [58]).

The character support of the nodes present in the most parsimonious reconstructions was calculated using two different methods. The first technique is the jackknife applied to character resampling [59]. The second method used is Bremer support [60,61], which evaluates node stability/sensitivity by exploring suboptimal tree solutions in order to determine how many additional steps must be allowed in searching for topologies before the hypothesized clade is no longer recovered. The jackknife support analysis was calculated using TNT [56,57]. The analysis was performed using 1,000 replicates for which the probability of independent character removal was set to 0.20. Each jackknife replicate was analyzed using a tree search strategy consisting of 10 replicates of RAS followed by TBR branch swapping (saving 10 trees per replicate). The topologies obtained during the jackknife replicates are summarized using GC frequencies [62]. Bremer support was calculated using the BREMER.RUN script provided with TNT.

### Sensitivity Analysis

The robustness of the phylogenetic results to competing hypotheses of eusuchian relationships was assessed two ways. First, positive tree constraints were defined and a maximum parsimony analysis was conducted using TNT enforcing the tree constraint during the search. Search parameters followed those of the primary analysis. Second, a Bayesian inference analysis was conducted using MrBayes v3.2 [63]. The standard model was used (Markov k-state variable model [Mkv] with a gamma-distributed rate variation) and the MCMC chain was run for 5 million generations with the first 25% of generations discarded as burn-in. Details of the constrained search and Bayesian analysis are included in Appendix III in S1 Document. and analysis files are available to download at http://www.morphobank.org/permalink/?P1200.

### Results

**Fig 14:**
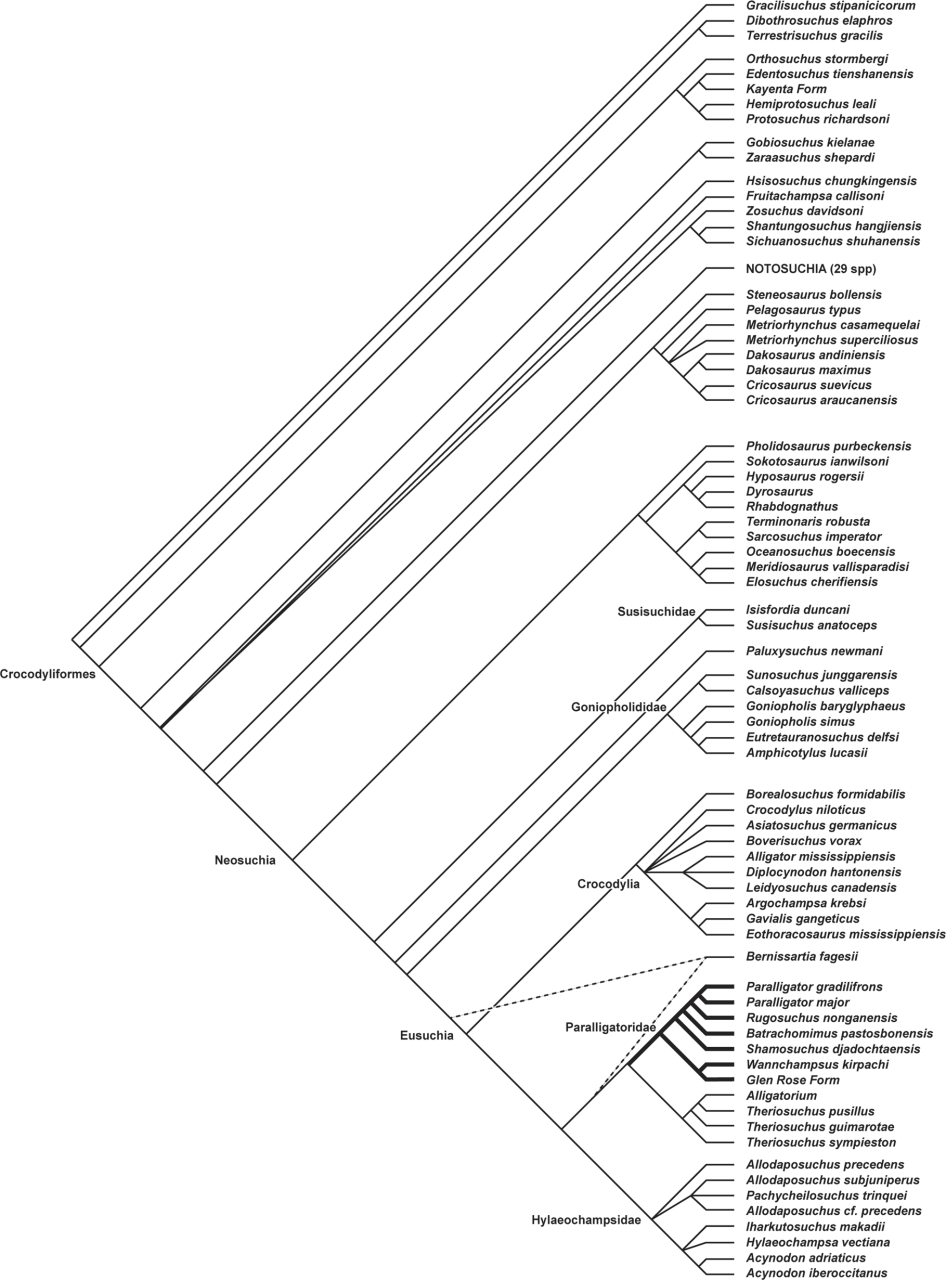
Reduced strict consensus of 108 equally optimal trees recovered from maximum parsimony analysis of 101 ingroup taxa and 318 phenotypic characters. Trees rooted on *Gracilisuchus stipanicicorum*. Two equally optimal positions of *Bernissartia fagesii* shown with dotted line (length = 1662, CI = 0.239, RI = 0.700).

Maximum parsimony analysis recovered 108 optimal trees with a length of 1662 steps (CI = 0.239, RI = 0.700). A reduced strict consensus of the trees (Fig. 14) deviates from the results of previous versions of this dataset [5,35,52] as well as many recent morphological analysis of Neosuchia [36,47,64]. Here paralligatorids and hylaeochampsids are not successive sister taxa to Crocodylia but instead form a speciose clade that is itself the sister to Crocodylia. *Isisfordia* and *Susisuchus* are recovered in a basally diverging position within Neosuchia, outside of the Goniopholididae node, not near the crown-group as in most previous analysis. Conversely *Theriosuchus* and *Alligatorium* are not recovered as basal neosuchians but instead nest within the paralligatorid + hylaeochampsid clade, as the sister group to Paralligatoridae. The monophyly of the three *Shamosuchus* species is not supported. *Shamosuchus major* and *S*. *gradilifrons* are sister taxa and sit in a derived position within Paralligatoridae. *Shamosuchus djadochtaensis* is near the base of Paralligatoridae with *Rugosuchus nonganensis* and *Batrachomimus pastosbonensis* more closely related to *S*. *major* and *S*. *gradilifrons* than to S. *djadochtaensis* (Fig. 14). I propose that the name *Paralligator* be reapplied to *S*. *major* and *S*. *gradilifrons*.

#### Position of Paralligatoridae within Neosuchia

Because of the two alternate positions *Bernissartia* can take in the most parsimonious trees (Fig. 14), the unequivocal character support is small placing paralligatorids within Eusuchia (i.e., the node containing *Hylaeochampsa* and Crocodylia—[65]). The presence of procoelous cervical and trunk vertebrae support the monophyly of Eusuchia and the placement of paralligatorids within it. This is mostly driven from the presence of procoely in *S*. *djadochtaensis*, *Wannchampsus*, and *Theriosuchus pusillus*. The presence or absence of this feature remains unknown for many basal eusuchians.

If *Bernissartia* is located outside the split between Crocodylia and Paralligatoridae then an axis vertebra with a wide neural spine supports eusuchian monophyly. This is not a particularly strong character because its distribution among neosuchians is at present poorly constrained. Wide axial neural spines (present in *S*. *djadochtaensis*, *Borealosuchus formidabilis* and *Boverisuchus vorax*, as well as in alligatorids) drive the current optimization at the base of Eusuchia. The form this character takes in hylaeochampsids is currently unknown.

If *Bernissartia* is located within Eusuchia on the stem of Paralligatoridae + Hylaeochampsidae, then three additional characters support the monophyly of this clade. Ancestrally, the eusuchian clade recovered in this analysis is characterized by derived morphology of a choanal groove undivided by a septum. Nearly all other crocodyliforms for which palate morphology is known have some form of choanal septum. An undivided choana is optimized at this node in the present analysis because *Shamosuchus djadochtaensis* (Fig. 3), the Glen Rose Form, hylaeochampsids and non-alligatorid crocodylians possess the condition. This trait is not without homoplasy, however, suggesting that the choanal septum reappeared multiple times–once within *Theriosuchus* and again within derived paralligatorid (e.g., *Batrachomimus*, *Paralligator major*, and *Paralligator gradilifrons*). The two additional characters supporting monophyly of the clade is the presence of a biconvex first caudal vertebrae and exposure of the supraoccipital on the skull roof. The presence of a biconvex first caudal is unknown in most of the derived neosuchians sampled. It is optimized as an ambiguous eusuchian synapomorphy here because of its presence in the crocodylians, *Bernissartia*, *Shamosuchus djadochtaensis*, and *Pachycheilosuchus trinquei*. Supraoccipital exposure on the skull roof is a common feature in many mesoeucrocodylians. Most notosuchians and advanced neosuchians posses the trait but it is currently optimized as independently derived at the eusuchian and ziphosuchian nodes.

Paralligatorids are more closely related to hylaeochampsids than to the crocodylian lineage based on three unambiguous synapomorphies. The presence of a long coracoid (i.e., 2/3 scapular length) currently diagnoses the Paralligatoridae + Hylaeochampsidae clade. This is an exceptionally weak synapomorphy. Only a few crocodyliforms are known from postcranial remains and those that are typically possess the derived morphology of a shorter coracoid relative to the scapula. The apomorphic reversal to the long coracoid morphology is optimized at the Paralligatoridae + Hylaeochampsidae because of its presence in *Theriosuchus pusillus*, *Alligatorium*, and *Pachycheilosuchus*; it is unknown in all other members of the clade.

In most neosuchians and thalattosuchians, the postzygapophyses of the axis are poorly developed. A reversal to the plesiomorphic condition of having well developed, laterally curving axial postzygapophyses is optimized here as a synapomorphy for Paralligatoridae + Hylaeochampsidae. Like the previous synapomorphy discussed, this one is only known in three of the members of the clade (*Theriosuchus guimarotae*, *S*. *djadochtaensis*, *Acynodon adriaticus*) and as such is quite sensitive to future sampling of this trait within the clade.

**Fig 15:**
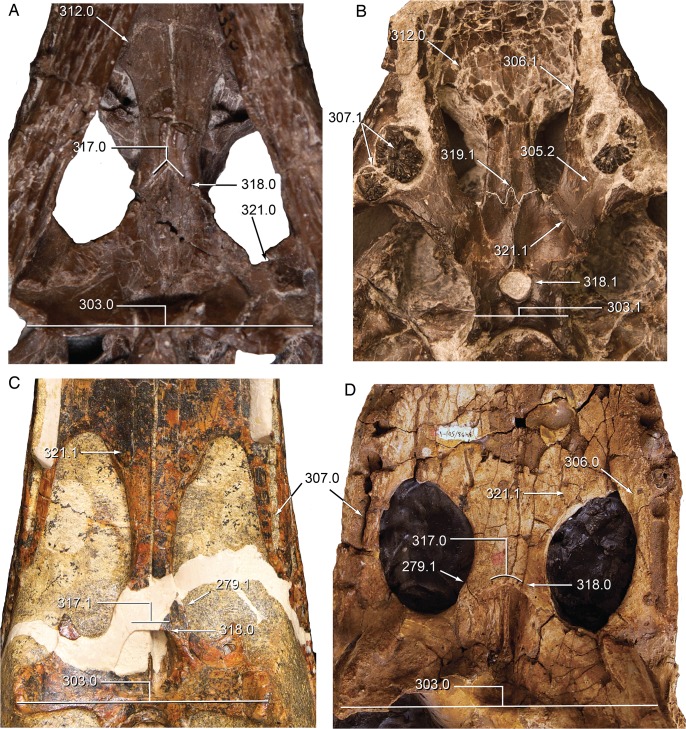
Systematic variation in palate morphology among neosuchians. Numbers indicate character number from phylogenetic dataset using in this study, with state value following the period (i.e., char.charstate). **A**, *Theriosuchus pusillus* (NHMUK 48330); **B**, *Iharkutosuchus makadii* (MTM 2006.53.1); **C**, *Isisfordia duncani* (QM F44320); **D**, *Shamosuchus ulgicus* (= *Paralligator gradilifrons*) (PIN 3458/501).

The third unambiguous synapomorphy at this node is also a reversal from a more widespread neosuchian condition. In most basal neosuchians and in all crocodylians examined, the palatal process of the maxilla at the contact with the palatine possesses a posteromedial process that curves around the anterior margin of the suborbital fenestra and onto the bony pharyngeal passage (Fig. 15). This process is particularly well developed in most crown group taxa (it is absent in *Gavialis*) and variably developed in more basal neosuchian forms. The process is absent in all hylaeochampsids as well as in *Theriosuchus*, *Batrachomimus* and *Rugosuchus* (Fig. 15). As such it is optimized as a synapomorphy uniting the group. Presence of the derived morphology in the Glen Rose Form and the two species of *Paralligator* is interpreted here as secondary derivations of the morphology.

#### Relationships within Paralligatoridae

Support for paralligatorid monophyly in this study is moderate. Four unambiguous synapomorphies unite paralligatorid taxa to the exclusion of *Theriosuchus*. Many neosuchians, as well as a few notosuchians like *Simosuchus* and *Araripesuchus*, possess a small vascular opening on the dorsal surface of the postorbital bar. This trait is lost independently in paralligatorids, *Paluxysuchus* and *Acynodon adriaticus*.

*Wannchampsus* and *Paralligator gradilifrons* have a medial quadrate condyle that expands ventrally, which is separated from the lateral condyle by a distinct intercondylar groove. This is optimized as a synapomorphy of paralligatorids, although the trait distribution is unknown in other members of the clade. *Shamosuchus djadochtaensis* is scored as unknown in the matrix but the IGM 100/1195 specimen strongly suggests this taxon has the morphology as well, and thus provides additional support for this as a paralligatorid synapomorphy. This feature of the quadrate is otherwise only seen in ziphosuchian notosuchians, wherein it is perhaps a bit more developed with a very strongly ventrally expanded medial condyle and a deep intercondylar groove (e.g., *Araripesuchus wegeneri*, *Anatosuchus minor*).

*Wannchampsus* and *Paralligator gradilifrons* share a wide and rounded olecranon process on the ulna. The distribution of this trait is poorly constrained among crocodyliforms generally. Crown group crocodylians similarly possess a wide and rounded olecranon. Similar to the previous character, *Shamosuchus djadochtaensis* is scored as unknown for this trait, but IGM 100/1195 suggests this taxon has a wide and rounded olecranon as well.

Paralligatorids are diagnosed by a midline crest on the basioccipital plate below the occipital condyle (Fig. 13). This trait appears convergently in multiple mesoeucrocodylian lineages, most notably within dyrosaurids, most notosuchians, derived hylaeochampsids, and most crocodylians. It is possible that future work will show this to be characteristic of a much more inclusive neosuchian clade.

Depending on whether character transformations are accelerated or delayed, an anterior half of the interfenestral bar between the suborbital fenestrae that flares anteriorly serves as a fifth synapomorphy for Paralligatoridae (Figs. 3C and, 5I, J). Pol et al. [5] initially reported this trait, but it is now clear that it has a wider distribution among neosuchians. Beyond being in all paralligatorids, an anteriorly flared interfenestral bar is present in *Acynodon*, *Allodaposuchus*, some goniopholidids, *Isisfordia*, and alligatorids. At present, an undivided foramen vagi ambiguously diagnoses this clade, but could be a synapomorphy of the more inclusive Paralligatoridae + *Theriosuchus* clade if the trait turns out to be present in *Theriosuchus*.

The monophyly of *Shamosuchus*, *Batrachomimus*, *Rugosuchus*, and *Paralligator* is supported by six synapomorphies. Of the paralligatorid taxa considered in this study, the phylogenetic analysis of Pol et al. [5] overlapped in the inclusion of *Shamosuchus djadochtaensis* and *Rugosuchus*. Their analysis identified and recovered as a synapomorphy for *Shamosuchus* + *Rugosuchus* a posteriorly flared posterior half of interfenestral bar between suborbital fenestrae (Fig. 3C, F). That trait continues to diagnosis a clade containing those two taxa but now also includes *Batrachomimus* and *Paralligator*. The morphology is quite similar to what is seen in the palate of alligatoroids.

Pol et al. [5] identified three other possible synapomorphies for a *Rugosuchus* + *Shamosuchus* clade. These traits where the absence of a ventrally open notch at the premaxilla/maxilla contact (Fig. 3A, D), a longitudinal ridge on the lateral surface of the jugal extending the entire length of the bone (Fig. 10), and a depression on the anterior area of the jugal below the orbits (Fig. 10A; [5]: Fig. 13]). Our expanded taxonomic sample now resolves these three traits as unambiguous synapomorphies for a taxonomically more inclusive clade. The absence of a notch at the premaxilla/maxilla contact marks a significant modification away from what is typically considered a hallmark neosuchian feature. However, as neosuchian sampling has increased the number of inferred losses of this notch as increased. At present, the modification to a smooth transition between premaxilla and maxilla occurred at least five times; once within paralligatorids, once in hylaeochampsids, once in susisuchids, once in derived gavialoids, and once in alligatoroids (globidontans + *Diplocynodon*). In fact, it is this modification that in many ways gives paralligatorids the “alligator” like appearance to their snout.

In *Shamosuchus*, *Batrachomimus*, and *Rugosuchus* the anterior edge of choanae is situated anteriorly, between suborbital fenestra. Although more posteriorly located in *Paralligator*, the anterior position is optimized as a synapomorphy for this subclade. Likewise, a reduced and sculptured outer lateral surface of the squamosal is optimized as a *Shamosuchus* + *Batrachomimus* + *Rugosuchus* + *Paralligator* synapomorphy, although *P*. *major* does exhibit the more derived condition of have an unsculptured and reduced lateral squamosal surface. Among neosuchians, only this clade of paralligatorids, *Acynodon*, and *Allodaposuchus subjuniperus* show the reduced but sculptured condition.

In *Batrachomimus* and *Paralligator* the choanal groove is completely septate. This is not the case in *Shamosuchus*, the Glen Rose Form or *Wannchampsus*, which have only partial choanal septa. The nature of the choanal septum is uncertain in *Rugosuchus*, but one would infer it to be completely septate based on its phylogenetic placement. Also, in *Batrachomimus*, *Rugosuchus*, and *Paralligator* the anterior extent of the jugal exceeds the anterior margin of the orbit. This is not true in *Shamosuchus*, the Glen Rose Form, or *Theriosuchus*, which have anteriorly shorter jugals.

*Rugosuchus* and *Paralligator* share the presence of a large lacrimal that, in dorsal view, is mediolaterally wider than the prefrontal, and contacts the nasal (Figs. 5G, J and 8E, F). Contact between the lacrimal and nasal is common among neosuchians, but basal members of Paralligatoridae, *Hylaeochampsa*, *Iharkutosuchus*, and *Theriosuchus guimarotae* all have lacrimals that fail to contact the nasal. Due to the distribution of these trait among the basal members of the Hylaeochampsidae + *Theriosuchus* + Paralligatoridae clade, a reversal to contacting lacrimal and nasals is here optimized as a synapomorphy for this subclade of paralligatorids. The presence of a mediolaterally wide prefrontal is convergently shared with a number of other neosuchian clades such as *Allodaposuchus*, *Acynodon*, Susisuchidae, and derived goniopholidids.

*Paralligator* is diagnosed by a choanae with the anterior edge situated near the posterior edge of the suborbital fenestra. The choanae of the other paralligatorids is located near the midpoint of the suborbital fenestra. The squamosal in *P*. *gradilifrons* and *P*. *major* has an anterior process that extends to the orbital margin, overlapping the postorbital bone. This process is not present in *Rugosuchus* and *Batrachomimus*. The only other taxa that share this feature are *Shamosuchus djadochtaensis* and the notosuchians *Malawisuchus* and *Simosuchus*. It is interesting that the three paralligatorids from the Gobi share this feature but are not united in a clade by it. Lastly, the two *Paralligator* species bear a posteromedial process on the palatal process of the maxilla that curves posteriorly onto the palatine-formed nasopharyngeal passage (Fig. 15C). This trait was discussed early because the loss of this process is optimized in the study as a synapomorphy of the Paralligatoridae + Hylaeochampsidae + *Theriosuchus* clade.

## Systematic paleontology

Crocodylomorpha Hay, 1930

Crocodyliformes Hay, 1930 (*sensu* Clark, 1986)

Neosuchia Clark in Benton and Clark, 1988

Eusuchia Huxley, 1875

### Paralligatoridae Konzhukova, 1954 [6]

#### Membership

*Paralligator* spp., *Wannchampsus kirpachi*, Glen Rose Form, *Rugosuchus nonganensis*, *Shamosuchus djadochtaensis*, and *Batrachomimus pastosbonensis*.

#### Diagnosis

Vascular opening in dorsal surface of postorbital bar absent (27.0); medial quadrate condyle expands ventrally, separated from lateral condyle by deep intercondylar groove (170.1); olecranon process of ulna wide and rounded (260.1); anterior half of interfenestral bar between suborbital fenestrae flared anteriorly (278.1); basioccipital midline crest present on basioccipital plate (297.1).

### *Shamosuchus* Mook, 1924 [1]

#### Type Species

*Shamosuchus djadochtaensis* Mook, 1924 [1]

#### Occurrence

Djadokhta Fm., Mongolia.

#### Diagnosis

Member of Paralligatoridae with the following combination of characters (* denotes autapomorphies in the context of other paralligatorids; numbers following trait denote characters from analysis with the convention of “character.character state”): a single wave of maxillary tooth enlargement (79.1)*; frontal with prominent midline crest and elevated orbital margins; preorbital ridge present but weakly developed; palpebral facets form prominent depressions; orbitonasal sulcus shallow on the maxilla*; postorbital-ectopterygoid contact absent (144.1)*; long splenial symphysis*; splenials V-shaped at symphysis; fourth maxillary tooth the largest; narrow ascending process of the quadratojugal bearing a slightly developed ridge located close to its anterior margin*; shallow and broad squamosal groove that tapers posteriorly at the level of the posterior edge of the otic aperture and reappears along the lateral edge of the posterolateral process of the squamosal; small siphoneal foramen located anteroventrally to the otic aperture; well-developed crest B on ventral surface of quadrate; undivided foramen vagi; cervical vertebrae procoelous, first dorsal vertebra procoelous with large neural canal.

#### Note

This diagnosis is abbreviated relative to that provided by Pol et al. [5]. Some of the features noted by those authors now serve as synapomorphies for more inclusive clades or have a more varied distribution than previously appreciated.

### *Shamosuchus djadochtaensis* Mook, 1924 [1]

#### Holotype

AMNH FARB 6412, a partial skull.

#### Diagnosis

As for genus.

### *Paralligator* Konzhukova, 1954 [6]

#### Type Species

*Paralligator gradilifrons* Konzhukova, 1954 [6]

#### Referred Taxa

*Paralligator major* Efimov, 1981 [8].

#### Synonymy

*Shamosuchus* Mook, 1924 [1] (in part).

#### Occurrence

Nemegt and Bayanshiree Fms., Mongolia.

#### Diagnosis

Member of Paralligatoridae with the following combination of characters: anterior edge of choanae situated near the posterior edge of suborbital fenestra (44.1); anterior process of squamosal, in lateral view, with anterior process extending to orbital margin and overlapping postorbital bone (288.1); palatal surface of maxilla bearing posteromedial process curving posteriorly onto palatine-formed nasopharyngeal passage (312.1).

### *Paralligator gradilifrons* Konzhukova, 1954 [6]

#### Holotype

PIN 554-1, a nearly complete skull lacking most of the secondary palate.

#### Synonymy

*Paralligator ancestralis* Konzhukova, 1954 [1]; *Shamosuchus ancestralis* (Konzhukova, 1954) [1]; *Paralligator ulgicus* Efimov, 1981 [8]; *Shamosuchus ulgicus* (Efimov, 1981) [8]; *Shamosuchus ulanicus* Efimov, 1983 [11]; *Shamosuchus tersus* Efimov, 1983 [11].

#### Diagnosis

Member of Paralligatoridae with the following combination of characters: narrow antero-posterior running groove through postorbital near postorbital-frontal contact; large crest A on ventral aspect of quadrate extending anteriorly above trigeminal foramen (may be fusion of crest A with A’; shares with *P*. *major* fifth maxillary tooth as largest; short mandibular symphysis ending near sixth mandibular tooth; choanal septum robust (compared to *Shamosuchus djadochtaensis*); groove runs through preorbital crest; majority of specimens possess small boss on anterolateral corner of postorbital; majority of specimens possess orbital crests on frontal similar in size to median frontal crest.

### *Paralligator major* Efimov, 1981 [8]

#### Holotype

PIN 3726/501, a nearly complete but heavily reconstructed skull.

#### Synonymy

There are currently no known synonyms.

#### Diagnosis

A member of *Paralligator* in which the premaxillae meet posterior to the external naris*.

## Discussion

The purpose of this study was to revise *Shamosuchus* and *Paralligator* taxonomy and provide a more comprehensive look at their phylogenetic history. Perhaps naively, I thought this would be a relatively straightforward task of sorting through the history of what was appreciated for some time as a likely over-split set of species [14]. The tasks, particularly the phylogenetic component, proved to be more complicated. Increased taxonomic and character sampling, as well as new discoveries such as *Paluxysuchus* [35], *Wannchampsus* [33], and *Batrachomimus* [36], and reevaluations of other derived eusuchians such as *Acynodon* [66] or putative eusuchians such as *Isisfordia* have begun to alter our understanding of the geographic and morphological variation within advanced neosuchians.

In assessing the morphological similarities among, and variation between, the numerous specimens of named *Shamosuchus* and *Paralligator* species, it became apparent that a host of morphological features are shared among the Bayanshiree and Nemegt Fms. specimens. While there are degrees of variation present between some specimens (particularly in the skull roof), establishing a nomenclatural framework that relies on apomorphy-based diagnoses [67,68,69] provides the most stable and precise taxonomy for *Shamosuchus* and *Paralligator*. Any attempt to separate a Nemegt taxon from a Bayanshiree taxon would have be driven strictly by a priori priority of temporal data and would necessarily have relied on hedges and poorly quantifiable distinctions, and thus diminished the diagnostic value of the material. Extant crocodylians can exhibit high degrees of variation [70], and one should not fear such variation in the fossil record.

The Paralligatoridae clade recovered here is similar in taxonomic content but not topology to the clade recovered by two other recent analyses [33,57]. Adams recovered the Glen Rose Form and *Wannchampsus* in the most derived position within the clade. Here I find them as the sister taxon to the remainder of paralligatorid diversity. The difference in the rooting of the paralligatorid clade between the two analysis appears to stem from the increase sampling of *Theriosuchus* in my analysis, the reevaluation of the posterior squamosal flange as a trait shared between *Theriosuchus* and other paralligatorids, and the discovery of additional morphological traits linking the group (e.g., the orbitonasal sulcus). Additionally, detailed comparison across neosuchians revealed that traits such as an open cranioquadrate canal, which formerly served to unite neosuchian subclades, is in fact the widespread condition for the group until you reach the crown. Similarly, the orbitonasal sulcus present in paralligatorids and *Theriosuchus* had been noted by Konzhukova [6] and Efimov [8] and recently identified in *Theriosuchus* species [47,51]. But these observations had yet to be translated into phylogenetic character data. Two of the synapomorphies Adams [35] recovered as diagnostic of paralligatorid (a midline ridge along the dorsal surface of the frontal and parietal; and a sharp ridge along the lateral surface of the angular) are optimized here as Paralligatoridae + *Theriosuchus* synapomorphies. Both of these traits had by identified as possible paralligatorid synapomorphies by Pol et al. [5] but their resolution was ambiguous in part because of the distribution of the traits in the Glen Rose Form and *Theriosuchus pusillus*.

A derived position for *Theriosuchus* is strongly supported in my analysis. Seven morphological traits unambiguously support the Paralligatoridae + *Theriosuchus* node. Support metrics are varied; a bootstrap analysis does not recover the node, jackknife support is low (GC = 22), and the Bremer support is 3. The monophyly of the core of Paralligatoridae (the node including *Shamosuchus* and *Paralligator*) is strongly supported with six unambiguous synapomorphies and moderate jackknife (GC = 31) and Bremer values (Bremer = 4).

Perhaps the most surprising result of the analysis was recovering a sister group relationship between Hylaeochampsidae and Paralligatoridae + *Theriosuchus*. As far as I can tell no such relationship has previously been proposed and it is not immediately obvious what would unite paralligatorids and *Theriosuchus* (which are all quite similar morphologically) with a clade so apomorphic and seemingly ecologically-specialized as hylaeochampsids. Indeed, the support for this grouping is very weak. All the diagnostic characters are all reversals from more widespread neosuchian traits. They are optimized as diagnostic for the clade based on observations from only a few taxa that are widely distributed within the clade. No support metric (Bremer, Jackknife, Bootstrap) recovered any support for the group. Constraining hylaeochampsids to be the sister taxon to the crown group to the exclusion of Paralligatoridae and *Theriosuchus* results in optimal trees that are only 2 steps longer than the most parsimonious trees in the unconstrained analysis (Fig. I in S1 Document). Bayesian inference of the morphological dataset supports the sister group relationships between Paralligatoridae and a *Theriosuchus* clade with over 90% posterior probability. It, however, recovers a traditional monophyletic Eusuchia without the odd result of paralligatorids nesting with hylaeochampsids (Fig. II in S1 Document).

If the current topology holds, the taxonomic content of Eusuchia will be greatly altered from our present understanding. In fact, things that do not have a “eusuchian” palate will nonetheless be eusuchians! Based on the present tree structure, the formation of a complete secondary palate is ambiguously optimized. It is equally most parsimonious to infer the derivation of a pterygoid-bound choanae at the Paralligatoridae + Crocodylia + Hylaeochampsidae node with a reversal at Paralligatoridae + *Theriosuchus*, or to infer the independent origin of the trait in Crocodylia and Hylaeochampsidae. I am skeptical of the placement of Paralligatoridae within Eusuchia proper. Resolution of this question ultimately rests with further taxonomic and character sampling. However, the non-existent clade support suggests this strange topology may be an artifact given that the only character transformations supporting it are in the form of reversals shared by two highly apomorphic clades. This notion is further indicated by the fact that the Bayesian inference tree does not recover the unexpected grouping of paralligatorids with hylaeochampsids. Unlike maximum parsimony, the Mkv model is sensitive to reversals occurring along long branches (like those that Paralligatoridae and Hylaeochampsidae sit on) and could be correctly identifying the shared reversals as convergence [71].

Likewise, another question unaddressed in the present study is how the repositioning of *Theriosuchus* and *Alligatorium* to a more derived neosuchian position effects notions of Atoposauridae monophyly. *Pachycheilosuchus* nests with *Allodaposuchus* in my analysis here, but prior versions of the data did recovery it near Atoposauridae [52]. Additionally, taxa such as *Unasuchus reginae* [72] and *Montsecosuchus depereti* [39] need to be reassessed in light of the new data arising from an increased focus on neosuchians.

## Conclusions

What once appeared to be a highly endemic Asian clade, Paralligatoridae now has a nearly global occurrence spanning the Late Jurassic of Brazil [36] through the Early Cretaceous of North America [33,35] into the Early through Late Cretaceous of Asian [1,6,8]. This temporal occurrence mirrors the temporal longevity of the paralligatorid sister taxon *Theriosuchus*, which is known from the Late Jurassic into the Late Cretaceous [47,48,54]. By corroborating the paralligatorid status of *Batrachomimus pastosbonensis*, this study contributes to the growing diversity of advanced Neosuchia that appear in the Southern Hemisphere. Although biogeographic conclusions for the transition to the crown group distribution are premature, there is certainly a new picture arising that is more complicated than a series of geographically restricted clades along the stem of Crocodylia. This ongoing rearrangement and clarification of the sister groups to Crocodylia are important for questions of basal crocodylian relationships including the placement of *Borealosuchus*, Planocraniidae [73], and perhaps even *Gavialis*. As sampling expands outside of Crocodylia, we are beginning to understand the extent to which alligator-like morphology pervades advanced neosuchians [66,74].

## Supporting Information

S1 Document Taxon list, phylogenetic dataset and sensitivity analysis results. (PDF)
