# Supplementary material for: A Review of Shamosuchus and Paralligator (Crocodyliformes, Neosuchia) from the Cretaceous of Asia
Source: PLoS One. 2015 Feb 25;10(2):e0118116. doi: 10.1371/journal.pone.0118116 (PMC4340866; doi:10.1371/journal.pone.0118116)
Supplement: S1 Document — (PDF) [file pone.0118116.s001.pdf]

A review of *Shamosuchus* and *Paralligator* (Crocodyliformes, Neosuchia) from the Cretaceous of Asia

ALAN H. TURNER

Department of Anatomical Sciences, Stony Brook University, Stony Brook, New York 11794-8081, U.S.A., alan.turner@stonybrook.edu

SUPPLEMENTARY DATA

SUPPLEMENTARY APPENDIX 1. Fossil taxa used in phylogenetic analysis. Bibliographic references and institutional accession numbers of the specimens that were scored based on firsthand examination are added after the taxon. To reproduce the taxon sampling scheme from the phylogenetic analysis, the following taxa have to be excluded from the MorphoBank dataset prior to running: *Candidodon itapecuruense*, *Trematochampsa*, *Stolokrosuchus lapparenti*, *Shamosuchus gradilifrons* holotype, *Shamosuchus tersus*, *Shamosuchus ancestralis*, *Shamosuchus ulanicus*, and *Shamosuchus ulgicus*. Alternatively, you can download the TNT file (shamo.matrix.final.txt) from the "Documents" section of the MorphoBank project (www.morphobank.org/permalink/P1200).

Acynodon adriaticus – Delfino et al., 2008; MCSNT 57248
Acynodon iberoccitanus – Buscalioni et al., 1997; Martin, 2007; ACAP-FX1; ACAP-FX2
Adamantinasuchus navae – Nobre and Carvalho, 2006
Alligator mississippiensis – Clark, 1994; Brochu, 1999; FMNH 8201
Alligatorium – Wellnhofer, 1971; Clark, 1986, 1994
Allodaposuchus precedens – Buscalioni et al., 2001; Delfino et al., 2008
Allodaposuchus cf. precedens – Martin, 2010; MDE/CM-616
Allodaposuchus subjuniperus – Puértolas-Pascual et al., 2013
Amphicotylus lucasii– Mook, 1964; AMNH 5782
Anatosuchus minor – Sereno et al., 2003; Sereno and Larsson, 2009; MNN GAD17; MNN GDF603
Araripesuchus buitreraensis – Pol and Apesteguía, 2005; MPCA-PV 235
Araripesuchus gomesii – Price, 1959; AMNH 24450
Araripesuchus patagonicus – Ortega et al., 2000; MUCPv nos. 267, 269, 270, 283
Araripesuchus tsangatsangana – Turner, 2006; FMNH nos. PR 2297–PR 2299; UA 8720
Araripesuchus wegeneri – Buffetaut, 1981; MNHN-GDF 700; MNN GAD19
Argochampsa krebsi – Hua and Jouve, 2004
Armadillosuchus arrudai – Marinho and Carvalho, 2009
"*Asiatosuchus*" *germanicus* – Brochu, 1999, 2003; SMF Me 1801; SMNK uncat.
Batrachomimus pastosbonensis – Montefeltro et al., 2013
Baurusuchus pachecoi – Price, 1945; DGM 299-R
Bernissartia fagesii – Buscalioni and Sanz, 1990; Norell and Clark, 1990; Brochu, 1999
Borealosuchus formidabilis – Erickson, 1976; Brochu, 1997b, 1999
Bretesuchus bonapartei – Gasparini et al., 1993; PVL 4735
Calsoyasuchus valliceps – Tykoski et al., 2002; TMM uncatalogued
Candidodon itapecuruense – Carvalho, 1994
Chimaerasuchus paradoxus – Wu and Sues, 1996; IVPP V8274
Comahuesuchus brachybuccalis – Bonaparte, 1991; MACN-PV N30, MACN-PV N31; MOZ P6131; MUCPv 202
Crocodylus niloticus – Clark, 1994; Brochu, 1999; FMNH 17157, FMNH 217153
Dakosaurus andiniensis – Vignaud and Gasparini, 1996; MHNSR PV 344; MOZ 6146P
Dakosaurus maximus – Fraas, 1902; BMNH R486; BSP AS.VI.1; SMNS 8203
Dibothrosuchus elaphros – Wu and Chatterjee, 1993; IVPP V 7907

Diplocynodon hantoniensis – Brochu, 1999; BMNH nos. 25178, 25199, 25245, 30206, 30210, 30219, 30236, 30250, 30289, 30362, 30368, 30391–30394, 30397, 30402, 30414, R1046, R1050, R5230
Dyrosaurus – Buffetaut, 1978; Clark, 1986, 1994; Jouve, 2005; BSP 1993.IX.400
Edentosuchus tienshanensis – Young, 1973; GMPKU-P 200101; IVPP V 3236
Elosuchus cherifiensis – Adams, 2013
Eothoracosaurus mississippiensis – Brochu, 2004; MSU 3293; PPM p2000.1.60
Eutretauranosuchus delfsi – Mook, 1967; Clark, 1986, 1994; AMNH 570; CMNH 8028
Fruita Form – Clark, 1985, 1994; LACM 120455a
Gavialis gangeticus – Clark, 1994; Brochu, 1999; FMNH 82681, FMNH 98864; MLP s/n
Geosaurus araucanensis – Gasparini and Dellapé, 1976; MACN-PV N 64, MACN-PV N 95; MLP nos. 72-IV-7-1–72-IV-7-4, 86-XI-5-7
Geosaurus suevicus – Fraas, 1902; SMNS [Fraas specimen]
Glen Rose Form – Brochu 1997a, 1999; MCZ 4453; USNM 22039,
Gobiosuchus kielanae – Osmólska, 1972; Osmólska et al., 1997; ZPAL nos. MgR-II/67–MgR-II/71
Goniopholis simus – Mook, 1942; Clark, 1986, 1994; Salisbury et al., 1999; BMNH 41098
Goniopholis baryglyphaeus – Schwarz, 2002
Gracilisuchus stipanicicorum – Romer, 1972; MCZ 4118; PVL 4597, PVL 4612
Hamadasuchus rebouli – Larsson and Sues, 2007; ROM 52620
Hemiprotosuchus leali – Bonaparte, 1971; PVL 3829
Hsisosuchus chungkingensis – Young and Chow, 1953; Li et al., 1994; Wu et al., 1994b; cast of CNM V 1090
Hylaeochampsa vectiana – Clark and Norell, 1992; BMNH R177
Hyposaurus rogersii – Troxell, 1925; Denton et al., 1997; YPM 764, YPM 985
Iberosuchus macrodon – Antunes, 1975; Ortega et al., 2000; UAM
Iharkutosuchus makadii – Osi, 2008; MTM nos. 2006.52.1, 2006.53.1, 2006.54.1,
2006.55.1, 2006.56.1, 2006.57.1, 2006.65.1, 2006.66.1, 2006.58.1
Isisfordia duncani – Salisbury et al., 2006; QM nos. F36211, F44320, F44319, F34642
Kaprosuchus saharicus – Sereno and Larsson, 2009; MNN IGU12
Kayenta Form – Clark, 1986; UCMP nos. 97638, 125359, 125871
Leidyosuchus canadensis – Brochu, 1997b, 1999, 2003; Wu et al., 2001b; AMNH 5352; NMC 2279; TMP 74.10.8
Libycosuchus brevirostris – Stromer, 1914; BSP 1912.VIII.574
Lomasuchus palpebrosus – Gasparini et al., 1991; MOZ 4084 PV
Mahajangasuchus insignis – Buckley and Brochu, 1999; Turner and Buckley, 2008; FMNH nos. PR 2389, PR 2448–PR 2450; UA nos. 8654, 9046, 9047, 9737
Malawisuchus mwakasyungutiensis – Clark et al., 1989; Gomani, 1997; MAL 45, MAL 49
Mariliasuchus amarali – Carvalho and Bertini, 1999; MNRJ 6298-V, MNRJ 6756-V; MZSP-PV 50, MZSP-PV 51
Meridiosaurus vallisparadisi– Adams, 2013
Metriorhynchus casamiquelai – Gasparini and Diaz, 1977; cast of MGHF 1.08573
Metriorhynchus superciliosus – Blainville, 1853; Wenz, 1968; AMNH 997, SMNS 10116

Montealtosuchus arrudacamposi – Carvalho et al., 2007
Notosuchus terrestris – Gasparini, 1971; MACN-PV RN nos. 1037, 1040–1044; MLP nos. 64-IV-16-1, 64-IV-16-5, 64-IV-16-6, 64-IV-16-10–64-IV-16-13, 64-IV-16-23; MPCA-PV 249, MPCA-PV250; MUCPv 287
Oceanosuchus boecensis – Adams, 2013
Orthosuchus stormbergi – Nash, 1975; SAM-K 409
Pachycheilosuchus trinquei – Rogers, 2003
Paluxysuchus newmani – Adams, 2013
Paralligator gradilifrons – Konzhukova, 1954; PIN 554-1; PIN 551-29/1; PIN 551-29/3; PIN 551-29/7; PIN 551-29/20; PIN 551-29/27; PIN 3458/501; PIN 3140-502; PIN 3141-501;
Paralligator major – Efimov, 1981; PIN 3726/501
Peirosaurus torminni – Price, 1955; Gasparini et al., 1991; MOZ 1750 PV
Pelagosaurus typus – Eudes-Deslongchamps, 1864; BMNH R32599; BSP 1925.I.34, BSP 1990.VIII.68; MB 1925.1, MB R.2883; SMNS 8666, SMNS 80066
Pholidosaurus purbeckensis – Owen, 1878; Clark, 1986, 1994; Salisbury, 2002; BMNH R3414
Pristichampsus vorax – Brochu, 1999; FMNH nos. PR 74, PR 399, PR 479; UCMP 154329
Protosuchus richardsoni – Colbert and Mook, 1951; AMNH 3024; MCZ 6727; UCMP 130860, UCMP 131827
Rhabdognathus – Brochu et al., 2002; CNRST-SUNY 190
Rugosuchus nonganensis – Wu et al., 2001a
Sarcosuchus imperator – Broin and Taquet, 1966; Sereno et al., 2001; MNN 603, MNN 604
Shamosuchus djadochtaensis – Mook, 1924; AMNH 6412; IGM 100/1195
Shantungosuchus hangjinensis – Wu et al., 1994a
Sichuanosuchus shuhanensis – Wu et al., 1997; IVPP V 10594
Simosuchus clarki – Buckley et al., 2000; FMNH nos. PR 2596–2599; UA nos. 8679, 9753, 9754, 9762, 9776
Sokotosuchus ianwilsoni – Halstead 1975; Buffetaut, 1979; Clark, 1986, 1994
Sphagesaurus huenei – Price, 1950; Pol, 2003; RCL 100
Sphagesaurus montealtensis – Andrade and Bertini, 2008a
Steneosaurus bollensis – Jaeger, 1828; Westphal, 1962; AMNH 5138; BSP nos. 1890.I.510, 1945.XV.1, 1949.XV.1, 1972.V.11, 1973.VII.592; GPIT Re.1193-6, GPIT Re.1193-12; MB nos. 1878.262, 1921.12, R.1953; SMNS nos. 115, 4554, 9427, 9428, 15951, 16848, 17484, 18878, 20280, 20282, 20283, 53422
Stolokrosuchus lapparenti – Larsson and Gado, 2000; MNN GDF600
Sunosuchus junggarensis – Wu et al., 1996
Susisuchus anatoceps – Salisbury et al., 2003; SMNK 3804 PAL
Terminonaris robusta – Mook, 1934; Wu et al., 2001c; AMNH 5849, AMNH 5850
Terrestrisuchus gracilis – Crush, 1984
Theriosuchus pusillus – Owen, 1879; Clark, 1986, 1994; Ortega et al., 2000; BMNH R48328, BMNH R48330
Theriosuchus guimarotae – Schwartz and Salisbury, 2005
Theriosuchus sympiestodon – Martin et al., 2010, 2014

Uberabasuchus terrificus – Carvalho et al., 2004
Uruguaysuchus aznarezi – Rusconi, 1933
Wannchampsus kirpachi – Adams, in press
Yacarerani boliviensis – Novas et al., 2009
Zaraasuchus shepardi – Pol and Norell, 2004b; IGM 100/1321
Zosuchus davidsoni – Pol and Norell, 2004a; IGM nos. 100/1304–100/1308

SUPPLEMENTARY APPENDIX 2. List of 321 phenotypic characters, 318 of which are used in the phylogenetic analysis. The character list is adapted from Turner and Sertich (2010), which in turn is based on that of Pol et al. (2009), Turner and Buckley (2008), Pol and Norell (2004a,b), Pol and Apesteguía (2005), and includes characters from Turner (2006), Brochu (1997a), Pritchard et al. (2013), and Adams (2013). Character definitions 1–101 are from Clark (1994) and have the same numeration as in the original publication. Character 5 was excluded from the analysis (due to dependence with the modified definition of character 6). Character 277 was excluded due to doubts of the homology coded for by the character. Inclusion of either of these characters, however, does not affect the outcome of the analysis (except for tree length). Character 281 was excluded because it is now interpreted as redundant with character 35. The additional characters are also listed here and their respective sources are cited along with the character number of the original publication. Characters 1, 3, 6, 10, 23, 37, 43–45, 49, 65, 67, 69, 73, 77, 79, 86, 90, 91, 96, 97, 104–106, 108, 126, 142, 143, 149, 167, 182, 197, and 226 represent potentially nested sets of homologies and/or entail presence and absence information. These characters were set as additive (also marked with a "+" in this list).

Character 1 (modified from Clark, 1994: char. 1): + External surface of dorsal cranial bones: smooth (0), slightly grooved (1) and heavily ornamented with deep pits and grooves (2).
Character 2 (modified from Clark, 1994: char. 2): Skull expansion at orbits: gradual (0), or abrupt (1).
Character 3 (modified from Clark, 1994: char. 3): + Rostrum proportions: narrow oreinirostral (0), broad oreinirostral (1), nearly tubular (2), or platyrostral (3).

Character 4 (Clark, 1994: char. 4): Premaxilla participation in internarial bar: forming at least the ventral half (0), or with little participation (1).
Character 5 (Clark, 1994: char. 5): Premaxilla anterior to nares: narrow (0), or broad (1).
Character 6 (modified from Clark, 1994: char. 6): + External nares facing anterolaterally or anteriorly (0), dorsally not separated by premaxillary bar from anterior edge of rostrum (1), or dorsally separated by premaxillary bar (2).
Character 7 (Clark, 1994: char. 7): Palatal parts of premaxillae: do not meet posterior to incisive foramen (0), or meet posteriorly along contact with maxillae (1).
Character 8 (Clark, 1994: char. 8): Premaxilla-maxilla contact: premaxilla loosely overlies maxilla (i.e. posterodorsal process of the premaxilla overlaps anterodorsal surface of the maxilla) (0), or sutured together along a butt joint (1).
Character 9 (modified from Clark, 1994: char. 9): Ventrally opened notch on ventral edge of rostrum at premaxilla-maxilla contact: absent (0), present as a notch (1), or present as a large fenestra (2).
Character 10 (modified from Clark, 1994: char. 10 by Pol et al., 2009): + Posterior palatal branches of maxillae anterior to palatines: do not meet (0), or meet extensively but posterior-most parts fail to meet (1), or meet entirely (2).
Character 11 (Clark, 1994: char. 11): Nasal contacts lacrimal (0), or do not contact (1).
Character 12 (Clark, 1994: char. 12): Lacrimal contacts nasal along medial edge only (0), or medial and anterior edges (1).
Character 13 (Clark, 1994: char. 13): Nasal contribution to narial border: yes (0), or no (1).
Character 14 (Clark, 1994: char. 14): Nasal-premaxilla contact: present (0), or absent (1).
Character 15 (modified from Clark, 1994: char. 15): Descending process of prefrontal: does not contact palate (0), or contacts palate (1).
Character 16 (Clark, 1994: char. 16): Postorbital-jugal contact: postorbital anterior to jugal (0), or postorbital medial to jugal (1), or postorbital lateral to jugal (2).
Character 17 (Clark, 1994: char. 17): Anterior part of the jugal with respect to posterior part: as broad (0), or twice as broad (1).

Character 18 (Clark, 1994: char. 18): Jugal bar beneath infratemporal fenestra: flattened (0), or rod-shaped (1).
Character 19 (Clark, 1994: char. 19): Quadratojugal dorsal process: narrow, contacting only a small part of postorbital (0), or broad, extensively contacting the postorbital (1).
Character 20 (Clark, 1994: char. 20): Frontal width between orbits: narrow, as broad as nasals (0), or broad, twice as broad as nasals (1).
Character 21 (Clark, 1994: char. 21): Frontals: paired (0), unpaired (1).
Character 22 (Clark, 1994: char. 22): Dorsal surface of frontal and parietal: flat (0), or with midline ridge (1).
Character 23 (modified from Clark, 1994: char. 23 by Buckley and Brochu, 1999: char. 81): + Parieto-postorbital suture: absent from dorsal surface of skull roof and supratemporal fossa (0), absent from dorsal surface of skull roof but broadly present within supratemporal fossa (1), or present within supratemporal fossa and on dorsal surface of skull roof (2).
Character 24 (Clark, 1994: char. 24): Supratemporal roof dorsal surface: complex (0), or dorsally flat "skull table" developed, with postorbital and squamosal with flat shelves extending laterally beyond quadrate contact (1).
Character 25 (modified from Clark, 1994: char. 25) Postorbital bar: sculpted (if skull sculpted) (0), or unsculpted (1).
Character 26 (modified from Clark, 1994: char. 26): Postorbital bar: transversely flattened (0), or cylindrical (1).
Character 27 (Clark, 1994: char. 27): Vascular opening in dorsal surface of postorbital bar: absent (0), or present (1).
Character 28 (modified from Clark, 1994: char. 28): Postorbital anterolateral process: absent or poorly developed (0), or well developed, long, and acute (1).
Character 29 (Clark, 1994: char. 29): Dorsal part of the postorbital: with anterior and lateral edges only (0), or with anterolaterally facing edge (1).
Character 30 (Clark, 1994: char. 30): Dorsal end of the postorbital bar broadens dorsally, continuous with dorsal part of postorbital (0), or dorsal part of the postorbital bar constricted, distinct from the dorsal part of the postorbital (1).

Character 31 (Clark, 1994: char. 31): Bar between orbit and supratemporal fossa broad and solid, with broadly sculpted dorsal surface if sculpture present (0), or bar narrow, sculpting restriced to anterior surface (1).
Character 32 (modified from Clark, 1994: char. 32): Parietal: with broad occipital portion (0), or without broad occipital portion (1).
Character 33 (Clark, 1994: char. 33) Parietal: with broad sculpted region separating fossae (0), or with sagittal crest between supratemporal fossae (1).
Character 34 (Clark, 1994: char. 34): Postparietal (dermosupraoccipital): a distinct element (0), or not distinct (fused with parietal?) (1).
Character 35 (Clark, 1994: char. 35): Posterodorsal corner of the squamosal: squared off, lacking extra "lobe" (0), or with unsculptured "lobe" (1).
Character 36 (modified from Clark, 1994: char. 36): Posterolateral process of squamosal: poorly developed and projected horizontally at the same level of the skull (0), elongated, thin, and posteriorly directed, not ventrally deflected (1), or elongated, posterolaterally directed, and ventrally deflected (2).
Character 37 (Clark, 1994: char. 37): + Palatines: do not meet on palate below the narial passage (0), form palatal shelves that do not meet (1), or meet ventrally to the narial passage, forming part of secondary palate (2).
Character 38 (Clark, 1994: char. 38): Pterygoid: restricted to palate and suspensorium, joints with quadrate and basisphenoid overlapping (0), or pterygoid extends dorsally to contact laterosphenoid and form ventrolateral edge of the trigeminal foramen, strongly sutured to quadrate and laterosphenoid (1).
Character 39 (modified from Clark, 1994: char. 39): Choanal opening: continuous with pterygoid ventral surface except for anterior and anterolateral borders (0), or opens into palate through a deep midline depression (choanal groove) (1).
Character 40 (Clark, 1994: char. 40): Palatal surface of pterygoids: smooth (0), or sculpted (1).
Character 41 (Clark, 1994: char. 41): Pterygoids posterior to choanae: separated (0), or fused (1).

Character 42 (modified from Clark, 1994: char. 42 by Ortega et al., 2000: char. 139): Depression on primary pterygoidean palate posterior to choana: absent or moderate in size being narrower than palatine bar (0), or wider than palatine bar (1).
Character 43 (Clark, 1994: char. 43):Primary pterygoidean palate, role in forming choanal opening: does not enclose choana (0); completely encloses choana (1).
Character 44 (modified from Pol and Norell, 2004 by Pol et al., 2009; modified from Clark, 1994: char. 44): + Anterior edge of choanae situated between the suborbital fenestra (or anteriorly) (0), situated near the posterior edge of suborbital fenestra (1), or near posterior edge of pterygoid flange (2).
Character 45 (Clark, 1994: char. 45): + Quadrate: without fenestrae (0), with single fenestrae (1), or with three or more fenestrae on dorsal and posteromedial surfaces (2).
Character 46 (Clark, 1994: char. 46): Posterior edge of quadrate: broad medial to tympanum, gently concave (0), or posterior edge narrow dorsal to otoccipital contact, strongly concave (1).
Character 47 (Clark, 1994: char. 47): Dorsal, primary head of quadrate articulates with: squamosal, otoccipital, and prootic (0), or with prootic and laterosphenoid (1).
Character 48 (Clark, 1994: char. 48): Ventrolateral contact of otoccipital with quadrate: very narrow (0), or broad (1).
Character 49 (Clark, 1994: char. 49): + Quadrate, squamosal, and otoccipital: do not meet to enclose cranioquadrate passage (0), enclose passage near lateral edge of skull (1), or meet broadly lateral to the passage (2).
Character 50 (Clark, 1994: char. 50): Pterygoid ramus of quadrate: with flat ventral edge (0), or with deep groove along ventral edge (1).
Character 51 (Clark, 1994: char. 51): Ventromedial part of quadrate: does not contact otoccipital (0), or contacts otoccipital to enclose carotid artery and form passage for cranial nerves IX--XI (1).
Character 52 (Clark, 1994: char. 52): Eustachian tubes: not enclosed between basioccipital and basisphenoid (0), or entirely enclosed (1).
Character 53 (Clark, 1994: char. 53): Basisphenoid rostrum (cultriform process): slender (0), or dorsoventrally expanded (1).

Character 54 (Clark, 1994: char. 54): Basipterygoid process: prominent, forming movable joint with pterygoid (0), or basipterygoid process small or absent, with basisphenoid joint suturally closed (1).
Character 55 (modified from Clark, 1994: char. 55 by Ortega et al., 2000: char. 68): Basisphenoid ventral surface: shorter than the basioccipital (0), or wide and similar to, or longer in length than basioccipital (1).
Character 56 (Clark, 1994: char. 56): Basisphenoid: exposed on ventral surface of braincase (0), or virtually excluded from ventral surface by pterygoid and basioccipital (1).
Character 57 (Clark, 1994: char. 57): Basioccipital: without well-developed bilateral tuberosities (0), or with large pendulous tubera (1).
Character 58 (Clark, 1994: char. 58): Otoccipital: without laterally concave descending flange ventral to subcapsular process (0), or with flange (1).
Character 59 (Clark, 1994: char. 59): Cranial nerves IX--XI: pass through common large foramen vagi in otoccipital (0), or cranial nerve IX passes medial to nerves X and XI in separate passage (1).
Character 60 (Clark, 1994: char. 60): Otoccipital: without large ventrolateral part ventral to paroccipital process (0), or with large ventrolateral part (1).
Character 61 (Clark, 1994: char. 61): Crista interfenestralis between fenestrae pseudorotunda and ovalis nearly vertical (0), or horizontal (1).
Character 62 (Clark, 1994: char. 62): Supraoccipital: forms dorsal edge of the foramen magnum (0), or otoccipitals broadly meet dorsal to the foramen magnum, separating supraoccipital from foramen (1).
Character 63 (Clark, 1994: char. 63): Mastoid antrum: does not extend into supraoccipital (0), or extends through transverse canal in supraoccipital to connect middle ear regions (1).
Character 64 (Clark, 1994: char. 64): Posterior surface of supraoccipital: nearly flat (0), or with bilateral posterior prominences (1).
Character 65 (modified from Clark, 1994: char. 65): + Palpebrals: absent (0), or one small palpebral present in orbit (1), or one large palpebral (2), or two large palpebrals (3).

Character 66 (Clark, 1994: char. 66): External nares: divided by a septum (0), or confluent (1).
Character 67 (Clark, 1994: char. 67): + Antorbital fenestra: as large as orbit (0), about half the diameter of the orbit (1), much smaller than the orbit (2), or absent (3).
Character 68 (modified from Clark, 1994: char. 68 by Ortega et al., 2000: char. 41): Supratemporal fenestrae extension: relatively large, covering most of surface of skull roof (0), or relatively short, fenestrae surrounded by a flat and extended skull roof (1).
Character 69 (modified from Clark, 1994: char. 69): + Choanal groove: undivided (0), partially septated (1), or completely septated (2).
Character 70 (Clark, 1994: char. 70): Dentary: extends posteriorly beneath mandibular fenestra (0), or does not extend beneath fenestra (1).
Character 71 (modified from Clark, 1994: char. 71): Retroarticular process: absent or extremely reduced (0), very short, broad, and robust (1), with an extensive rounded, wide, and flat (or slightly concave) surface projected posteroventrally and facing dorsomedially (2), posteriorly elongated, triangular-shaped and facing dorsally (3), or posteroventrally projecting and paddle-shaped (4).
Character 72 (Clark, 1994: char. 72): Prearticular: present (0), or absent (1).
Character 73 (modified from Clark, 1994: char. 73): + Articular without medial process (0), with short process not contacting braincase (1), or with process articulating with otoccipital and basisphenoid (2).
Character 74 (Clark, 1994: char. 74): Dorsal edge of surangular: flat (0), or arched dorsally (1).
Character 75 (Clark, 1994: char. 75): Mandibular fenestra: present (0), or absent (1).
Character 76 (Clark, 1994: char. 76): Insertion area for M. pterygoideous posterior: does not extend onto lateral surface of angular (0), or extends onto lateral surface of angular (1).
Character 77 (modified from Clark, 1994: char. 77): + Splenial involvement in symphysis in ventral view: not involved (0), involved slightly in symphysis (1), or extensively involved (2).
Character 78 (modified from Clark, 1994: char. 78 by Turner and Sertich, 2010): Posterior premaxillary teeth, size: similar in size to anterior teeth (0); longer but does not

form an enlarged caniniform tooth (1); much longer forming one large premaxillary caniniform tooth (2); much longer forming two large premaxillary caniniform teeth (3).

Character 79 (modified from Clark, 1994: char. 79): + Maxillary teeth waves: absent, no tooth size variation (0), one wave of teeth enlarged (1), or enlarged maxillary teeth curved in two waves (festooned) (2).

Character 80 (Clark, 1994: char. 80): Anterior dentary teeth opposite premaxilla-maxilla contact: no more than twice the length of other dentary teeth (0), or more than twice the length (1).

Character 81 (modified from Clark, 1994: char. 81): Dentary teeth posterior to tooth opposite premaxilla-maxilla contact: equal in size (0), or enlarged dentary teeth opposite to smaller teeth in maxillary toothrow (1).

Character 82 (modified from Clark, 1994: char. 82 by Ortega et al., 2000: char. 120): Anterior and posterior scapular edges: symmetrical in lateral view (0), anterior edge more strongly concave than posterior edge (1), or dorsally narrow with straight edges (2).

Character 83 (modified from Clark, 1994: char. 83 by Ortega et al., 2000: char. 121): Coracoid length: up to two-thirds of the scapular length (0), or subequal in length to scapula (1).

Character 84 (Clark, 1994: char. 84): Anterior process of ilium: similar in length to posterior process (0), or one-quarter or less of the length of the posterior process (1).

Character 85 (Clark, 1994: char. 85): Pubis: rodlike without expanded distal end (0), or with expanded distal end (1).

Character 86 (Clark, 1994: char. 86): + Pubis: forms anterior half of ventral edge of acetabulum (0), or pubis contacting the ilium but partially excluded from the acetabulum by the anterior process of the ischium (1), or pubis completely excluded from the acetabulum by the anterior process of the ischium (2).

Character 87 (Clark, 1994: char. 87): Distal end of femur: with large lateral facet for the fibula (0), or with very small facet (1).

Character 88 (Clark, 1994: char. 88): Fifth pedal digit: with phalanges (0), or without phalanges (1).

Character 89 (Clark, 1994: char. 89): Atlas intercentrum: broader than long (0), or as long as broad (1).

Character 90 (modified from Clark, 1994: char. 90): + Cervical neural spines: all anteroposteriorly large (0), only posterior ones rodlike (1), or all spines rodlike (2).
Character 91 (modified from Clark, 1994: char. 91 by Buscalioni and Sanz, 1988: char. 37 and by Brochu, 1997a: char. 7): + Hypapophyses in cervicodorsal vertebrae: absent (0), present only in cervical vertebrae (1), present in cervical and the first two dorsal vertebrae (2), present up to the third dorsal vertebra (3), or up to the fourth dorsal vertebrae (4).
Character 92 (Clark, 1994: char. 92): Cervical vertebrae: amphicoelous or amphyplatian (0), or procoelous (1).
Character 93 (Clark, 1994: char. 93): Trunk vertebrae: amphicoelous or amphyplatian (0), or procoelous (1).
Character 94 (Clark, 1994: char. 94): All caudal vertebrae: amphicoelous or amphyplatian (0), first caudal biconvex with other procoelous (1), or procoelous (2).
Character 95 (modified from Clark, 1994: char. 95 by Turner and Sertich, 2010): Dorsal osteoderms, shape: rounded or ovate (0); rectangular, broader than long (1); square (2); rectangular, longer than broad (3).
Character 96 (modified from Clark, 1994: char. 96, and Brochu, 1997a: char. 40): + Dorsal osteoderms: without articular anterior process (0), with a discrete convexity on anterior margin (1), or with a well-developed process located anterolaterally in dorsal parasagittal osteoderms (2).
Character 97 (modified from Clark, 1994: char. 97 by Ortega et al., 2000: chars. 107 and 108 and this paper): + Rows of dorsal primary osteoderms (sensu Frey, 1988): two parallel rows (0), more than two (1), or more than four (2).

Character 98 (Clark, 1994: char. 98): Osteoderms: some or all imbricated (0), or sutured to one another (1).
Character 99 (Clark, 1994: char. 99): Tail osteoderms: dorsal only (0), or completely surrounded by osteoderms (1).
Character 100 (Clark, 1994: char. 100): Trunk osteoderms: absent from ventral part of the trunk (0), or present (1).

Character 101 (Clark, 1994: char. 101): Osteoderms: with longitudinal keels on dorsal surfaces (0), or without longitudinal keels (1).

Character 102 (Wu and Sues, 1996: char. 14): Jugal: participating in margin of antorbital fossa (0), or separated from it (1).

Character 103 (modified from Wu and Sues, 1996: char. 17): Mandibular symphysis in lateral view: shallow and tapering anteriorly (0), deep and tapering anteriorly (1), deep and anteriorly convex (2), or shallow and anteriorly convex (3).

Character 104 (modified from Wu and Sues, 1996: char. 23): + Articular facet for quadrate condyle: equal in length to the quadrate condyles (0), slightly longer (1), or close to three times the length of the quadrate condyles (2).

Character 105 (modified from Wu and Sues, 1996: char. 24 and Wu et al., 1997: char. 124): + Jaw joint: placed at level with occipital condyle (0), below basioccipital condyle about above level of lower toothrow (1), or below level of toothrow (2).

Character 106 (modified from Wu and Sues, 1996: char. 27 and Ortega et al., 2000: char. 133): + Premaxillary tooth number: six (0); five (1); four (2); three (3); two (4); one (5).

Character 107 (modified from Wu and Sues, 1996: char. 29): Unsculptured region along alveolar margin on lateral surface of maxilla: absent (0), or present (1).

Character 108 (Wu and Sues, 1996: char. 30): + Maxilla, number of teeth: eight or more (0); seven (1); six (2); five (3); four (4).

Character 109 (Wu and Sues, 1996: char. 33): Coracoid, posteromedial or ventromedial process: absent (0); elongate posteromedial process present (1); distally expanded ventromedial process present (2).

Character 110 (Wu and Sues, 1996: char. 40): Radiale and ulnare, size: short and massive (0); elongate (1).

Character 111 (modified from Gomani, 1997: char. 4): Prefrontals anterior to orbits: elongated, oriented parallel to anteroposterior axis of the skull (0); short and broad, oriented posteromedially-anterolaterally (1).

Character 112 (modified from Gomani, 1997: char. 32): Basioccipital and ventral part of otoccipital, orientation: facing posteriorly (0); posteroventrally (1).

Character 113 (Buscalioni and Sanz, 1988: char. 35): Vertebral centra, shape: cylindrical (0); spool shaped (1).
Character 114 (modified from Buscalioni and Sanz, 1988: char. 39): Transverse process of posterior dorsal vertebrae, shape: dorsoventrally low and laminar (0); dorsoventrally high (1).
Character 115 (Buscalioni and Sanz, 1988: char. 44): Number of sacral vertebrae two (0); more than two (1).
Character 116 (Buscalioni and Sanz, 1988: char. 49): Supra-acetabular crest: present (0); absent (1).
Character 117 (Buscalioni and Sanz, 1988: char. 54): Proximal end of radiale, shape: expanded symmetrically, similarly to the distal end (0); more expanded proximolaterally than proximomedially (1).
Character 118 (Ortega et al., 1996: char. 5): Lateral surface of the anterior region of surangular and posterior region of dentary: without a longitudinal depression (0); with a longitudinal depression (1).
Character 119 (Ortega et al., 1996: char. 9): Ventral exposure of splenials: absent (0); present (1).
Character 120 (modified from Andrade and Bertini, 2008a: char. 120; Ortega et al., 1996: char. 11; Ortega et al., 2000: char. 100): Tooth margin carinae: without carinae or with smooth or crenulated carinae (0); with homogeneous denticulate carinae (denticles are small and symmetrical in form as in ziphodont teeth (1); with heterogeneous carinae possessing rounded tubercle-like denticles, developed preferentially along posterior margin (2).
Character 121 (modified from Pol, 1999a: char. 133 and Ortega et al., 2000: char. 145): Lateral surface of anterior process of jugal: flat or convex (0); with broad shelf below the orbit with triangular depression underneath it (1).
Character 122 (Pol, 1999a: char. 134): Jugal, extension below the orbit: does not exceed the anterior margin of orbit (0); exceeds margin of orbit (1).
Character 123 (Pol, 1999a: char. 135): Notch in premaxilla on lateral edge of external nares: absent (0); present on the dorsal half of the external nares lateral margin (1).

Character 124 (Pol, 1999a: char. 136): Dorsal border of external nares: formed mostly by the nasals (0); formed by both the nasals and premaxilla (1).
Character 125 (Pol, 1999a: char. 138): Posterodorsal process of premaxilla: absent (0); present extending posteriorly wedging between maxilla and nasals (1).
Character 126 (Pol, 1999a: char. 139 and Ortega et al., 2000: char. 9): + Premaxilla-maxilla suture in palatal view, medial to alveolar region, orientation of suture: anteromedially directed (0); sinusoidal, posteromedially directed on its lateral half and anteromedially directed along its medial region (1); posteromedially directed (2); straight (3); posteromedially curved (U shaped) (4).
Character 127 (Pol, 1999a: char. 140): Nasal lateral border posterior to external nares: laterally concave (0); straight (1).
Character 128 (Pol, 1999a: char. 141): Nasal lateral edges: nearly parallel (0); oblique to each other converging anteriorly (1); oblique to each other diverging anteriorly (2).
Character 129 (Pol, 1999a: char. 143): Palatine anteromedial margin: exceeding the anterior margin of the palatal fenestrae extending anteriorly between the maxillae (0); not exceeding the anterior margin of palatal fenestrae (1).
Character 130 (Pol, 1999a: char. 144): Dorsoventral height of jugal antorbital region with respect to infraorbital region equal or lower (0); antorbital region more expanded than infraorbital region of jugal (1).
Character 131 (Pol, 1999a: char. 145): Maxilla-lacrimal contact: partially included in antorbital fossa (0); completely included (1).
Character 132 (Pol, 1999a: char. 146): Lateral eustachian tube openings, location: located posteriorly to the medial opening (0); aligned anteroposteriorly and dorsoventrally (1).
Character 133 (Pol, 1999a: char. 147): Anterior process of ectopterygoid: developed (0); reduced-absent (1).
Character 134 (Pol, 1999a: char. 148): Posterior process of ectopterygoid: developed (0); reduced-absent (1).
Character 135 (Pol, 1999a: char. 149 and Ortega et al., 2000: char. 13):Small foramen located in teh premaxillo-maxillary suture in lateral surface (not for big mandibular teeth): absent (0); present (1).

Character 136 (Pol, 1999a: char. 150): Jugal posterior process, extent of process: exceeding posteriorly the infratemporal fenestrae (0); does not exceed infratemporal fenestrae (1).
Character 137 (Pol, 1999a: char. 151): Compressed crown of maxillary teeth, orientation: oriented paraleel to the longitudinal axis of skull (0); obliquely disposed (1).
Character 138 (Pol, 1999a: char. 152): Large and aligned neurovascular foramina on lateral maxillary surface: absent (0); present (1).
Character 139 (modified from Pol, 1999a: char. 153): External surface of maxilla and premaxilla, general shape: with a single plane facing laterally (0); with ventral region facing laterally and dorsal region facing dorsolaterally (1).
Character 140 (modified from Pol, 1999a: char. 154; Ortega et al., 2000: char. 104; Andrade and Bertini, 2008a: char. 135): +Maxillary teeth, lateral compression: absent (0); present, compression assymetrically occuring only along distal margin giving teeth a teardrop shape (1); present, lateral compression symmetrically developed (2).
Character 141 (Pol, 1999a: char. 155): Posteroventral corner of quadratojugal: reaches the quadrate condyles (0); does not reach the quadrate condyles (1).
Character 142 (modified from Pol, 1999a: char. 156): + Base of postorbital process of jugal, orientation: directed posterodorsally (0); directed dorsally (1); directed anterodorsally (2).
Character 143 (Pol, 1999a: char. 157): + Postorbital process of jugal, location on jugal: anteriorly placed (0); in the middle (1); posteriorly places (2).
Character 144 (Pol, 1999a: char. 158 and Ortega et al., 2000: char. 36): Postorbital-ectopterygoid contact: present (0); absent (1).
Character 145 (Pol, 1999a: char. 161): Quadratojugal, ornamentation: absent (0); ornamented on the base (1).
Character 146 (Pol, 1999a: char. 162): Prefrontal-maxillary contact in the inner anteromedial region of orbit absent (0); present (1).
Character 147 (Pol, 1999a: char. 163): Basispenoid, exposure on braincase: without lateral exposure (0); with lateral exposure (1).
Character 148 (Pol, 1999a: char. 165): Quadrate process of pterygoids: well developed (0); poorly developed (1).

Character 149 (modified from Pol, 1999a: char. 166 and Ortega et al., 2000: char. 44): + Quadrate major axis, direction of orientation: posteroventrally (0); ventrally (1); anteroventrally (2).
Character 150 (Pol, 1999a: char. 167): Quadrate distal end: with only one plane facing posteriorly (0); with two distinct faces in posterior view, a posterior one and amedial one bearing the foramen aereum (1).
Character 151 (Pol, 1999a: char. 168): Anteroposterior development of neural spine in axis: well developed covering all the neural arch length (0); poorly developed, located over the posterior half of the neural arch (1).
Character 152 (Pol, 1999a: char. 169): Prezygapophyses of axis, development relative to neural arch: not exceeding edge of neural arch (0); exceeding the anterior margin of neural arch (1).
Character 153 (Pol, 1999a: char. 170): Postzygapophyses of axis: well developed, curved laterally (0); poorly developed (1).
Character 154 (modified from Pol, 1999b: char. 212): Shape of dentary symphysis in ventral view: tapering anteriorly forming an angel (0); U-shaped, smoothly curving anteriorly (1); lateral edges longitudinally oriented, convex anterolateral corner, and extensive transversally oriented anterior edge (2).
Character 155 (Pol, 1999b: char. 213): Unsculpted region in the dentary below the tooth row: absent (0); present (1).
Character 156 (Buckley and Brochu, 1999: char. 102): Suranglur, contribution to the glenoid fossa: forms only the lateral wall of glenoid (0); forms approximately one-third of the glenoid (1).
Character 157 (modified from Buckley and Brochu, 1999: char. 102): Femur, anterior margin: linear (0); bears flange for coccygeofemoralis musculature (1).
Character 158 (modified from Buckley and Brochu, 1999: char. 105): Dentary, lateral surface: smooth lateral to seventh alveolous (0); with lateral concavity for the reception of the enlarged maxillary tooth (1).
Character 159 (modified from Ortega et al., 1996: char. 1 and Buckley and Brochu, 1999: char. 107): Dorsal edge of dentary: slightly concave or straight and subparallel to the longitudinal axis of skull (0); straight with an abrupt dorsal expansion, being straight

posteriorly (1); with a single dorsal expansion and concave posterior to this (2); sinusoidal, with two concave waves (3).

Character 160 (modified from Ortega et al., 1996: char. 2 and Buckley and Brochu, 1999: char. 108): Dentary compression and lateroventral surface anterior to mandibular fenestra: compressed and vertical (0); not compressed and convex (1).

Character 161 (Ortega et al., 1996: char. 7 and Buckley and Brochu, 1999: char. 110): Splenial posterior to symphysis: thin (0); robust dorsally (1).

Character 162 (Ortega et al., 1996: char. 13 and Buckley et al., 2000: char. 117): Cheek teeth: not constricted at base of crown (0); constricted (1).

Character 163 (Ortega et al., 2000: char. 10): Ventral edge of premaxilla, location relative to maxilla: at the same height as the ventral edge of maxilla (0); located deeper, with the dorsal contour of anterior part of dentary strongly concave (1).

Character 164 (modified from Ortega et al., 2000: char. 19): Maxillary dental implantion: teeth in isolated alveoli (0); located on a dental groove (1).

Character 165 (Ortega et al., 2000: char. 24): Caudal tip of nasals: converge at sagittal plane (0); caudally separated by anterior sagittal projection of frontals (1).

Character 166 (Ortega et al., 2000: char. 33): Relative length between squamosal and postorbital: squamosal is longer (0); postorbital is longer (1).

Character 167 (modified from Ortega et al., 2000: character 34): + Jugal portion of postorbital bar, relative to lateral surface of jugal: flush with lateral surface (0); anteriorly continuous but posteriorly inset (1); medially displaced and a ridge separate postorbital bar from lateral surface of jugal (2).

Character 168 (Ortega et al., 2000: char. 42): Outer surface of squamosal laterodorsally oriented: extensive (0); reduced and sculpted (1); reduced and unsculpted (2).

Character 169 (Ortega et al., 2000: char. 47): Quadratojugal spine at caudal margin of infratemporal fenestra: absent (0); present (1).

Character 170 (modified from Ortega et al., 2000: char. 53): Quadrate condyles: poorly developed intercondylar groove (0); medial condyle expands ventrally, being separate from the lateral condyle by a deep intercondylar groove (1).

Character 171 (Ortega et al., 2000: char. 62): Exposure of supraoccipital in skull roof: absent (0); present (1).

Character 172 (Ortega et al., 2000: char. 70): Nasal participation in antorbital fenestra: present (0); absent (1).
Character 173 (Ortega et al., 2000: char. 75): Anterior opening of temporo-orbital canal, in dorsal: exposed (0); hidden in dorsal view and overlapped by squamosal rim of supratemporal fossa (1).
Character 174 (Ortega et al., 2000: char. 90): Foramen intramandibularis oralis: small or absent (0); big and slotlike (1).
Character 175 (modified from Ortega et al., 2000: char 98): Coronoid size: short and located below the dorsal edge of the mandibular ramus (0); anteriorly extended with posterior region elevated at the dorsal margin of the mandibular ramus (1).
Character 176 (Ortega et al., 2000: char. 101): Width of root of teeth with respect to crown: narrower or equal (0); wider (1).
Character 177 (Ortega et al., 2000: char. 109): Gap in cervico-thoracic dorsal armor: absent (0); present (1).
Character 178 (Ortega et al., 2000: char. 130): Lateral contour of snout in dorsal view: straight (0); sinusoidal (1).
Character 179 (Ortega et al., 2000: char. 138): Pterygoidean flanges: laminar and expanded (0); bar-like and elongated (1); bar-like and poorly developed (2).
Character 180 (Ortega et al., 2000: char. 146): Ectopterygoid medial process, shape: single process (0); forked (1).
Character 181 (modified from Ortega et al., 2000: char. 157): Skull roof, shape in dorsal view: rectangular (0); trapezoidal (1).
Character 182 (Ortega et al., 2000: char. 30): + Prefrontal pillars when integrated in palate: pillars transversely expanded (0); transversely expanded in their dorsal part and columar ventrally (1); longitudinally expanded in their dorsal part and columnar ventrally (2).
Character 183 (Ortega et al., 2000: char. 21): Ventral edge of maxilla in lateral view: straight or convex (0); sinusoidal (1).
Character 184 (modified from Ortega et al., 2000: char. 156): Position of first enlarged maxillary teeth: second or third alveoli (0); fourth or fifth (1).

Character 185 (Pol and Apesteguía, 2005: char. 180): Splenial-dentary suture at symphysis on ventral surface: v-shaped (0); transversal (1).
Character 186 (Pol and Apesteguía, 2005: char. 181): Posterior peg at symphysis: absent (0); present (1).
Character 187 (Pol and Apesteguía, 2005: char. 182): Posterior ridge on glenoid fossa of articular: present (0); absent (1).
Character 188 (modified from Pol et al., 2009; previously from Gomani, 1997: char. 46; Buckley et al., 2000: char. 113; Andrade and Bertini, 2008a: char 149): Cusps of teeth, number and conformation: one unique cusp (0); one main cusp with smaller cusps arranged in one row (1); one main cusp with smaller cusps arranged in more than one row (2); several cusps of equal size arranged in more than one row (3); multiple small cusps along edges of occlusal surface (4).
Character 189 (Pol and Apesteguía, 2005: char. 184): Dorsal surface of mandibular symphysis: flat or slightly concave (0); strongly concave and narrow, trough shaped (1).
Character 190 (Pol and Apesteguía, 2005: char. 185): Medial surface of splenials posterior to symphysis: flat or slightly convex (0); markedly concave (1).
Character 191 (Pol and Apesteguía, 2005: char. 186): Choanal septum shape: narrow vertical bony sheet (0); T-shaped bar expanded ventrally (1); massive and blocky (2).
Character 192 (Pol and Norell, 2004a: char. 164): Cross section of distal end of quadrate: mediolaterally wide and anteroposteriorly thin (0); subquadrangular (1).
Character 193 (Pol and Apesteguía, 2005: char. 188): Lateral surface of dentaries below alveolar margin, at mid to posterior region of tooth row: vertically oriented, continuous with rest of lateral surface of the dentaries (0); flat surface exposed laterodorsally, divided by a ridge from rest of the lateral surface of the dentaries (1).
Character 194 (Pol and Norell, 2004a: char. 165): Palatine-pterygoid contact on palate: palatines overlies pterygoids (0); palatines firmly sutured to pterygoids (1).
Character 195 (Pol et al., 2004: char. 164): Ectopterygoid main axis orientation: laterally or slightly anterolaterally (0); anteriorly, subparallel to the skull longitudinal axis (1).
Character 196 (Wu et al., 1997: char. 103): Squamosal descending process: absent (0); present (1).

Character 197 (modified from Wu et al., 1997: char. 105): + Development of distal quadrate body ventral to otoccipital-quadrate contact: distinct (0); incipiently distinct (1); indistinct (2).
Character 198 (Wu et al., 1997: char. 106): Pterygoid flanges, size: thin and laminar (0); dorsoventrally thick, with pneumatic spaces (1).
Character 199 (Wu et al., 1997: char. 108): Postorbital participation in infratemporal fenestra: almost or entirely excluded (0); bordering infratemporal fenestra (1).
Character 200 (Wu et al., 1997: char. 109): Palatines, contribution to suborbital fenestra: form margin of suborbital fenestra (0); excluded from margin of suborbital fenestra (1).
Character 201 (Wu et al., 1997: char. 110): Angular posterior to mandibular fenestra, location on mandible: widely exposed on lateral surface of mandible (0); shifted to the ventral surface of mandible (1).
Character 202 (Wu et al., 1997: char. 112): Posteroventral edge of mandibular ramus, shape: straight or convex (0); markedly deflected (1).
Character 203 (modified from Wu et al., 1997: char. 119):Quadrate ramus of pterygoid, width in ventral view: narrow (0); broad (1).
Character 204 (Wu et al., 1997: char. 121): Pterygoids, contact on palate: not in contact anterior to basisphenoid on palate (0); pterygoids in contact (1).
Character 205 (modified from Wu et al., 1997: char. 122): Olecranon: well developed (0); absent (1).
Character 206 (Wu et al., 1997: char. 123): Cranial table width with respect to ventral portion of skull: as wide as ventral portion (0); narrower than ventral portion of skull (1).
Character 207 (Wu et al., 1997: char. 127): Depression on posterolateral surface of maxilla: absent (0); present (1).
Character 208 (modified from Wu et al., 1997: char. 128 by Pol et al., 2009): Paired anterior palatal fenestra: absent (0); present (1).
Character 209 (Pol and Norell, 2004a: char. 179): Paired ridges located medially on ventral surface of basisphenoid: absent (0); present (1).
Character 210 (Pol et al., 2004a: char. 179): Ventral margin of infratemporal bar of jugal: straight (0); dorsally arched (1).

Character 211 (Pol and Norell, 2004a: char. 180): Posterolateral end of quadratojugal, shape and relationship with quadrate: acute or rounded, tightly overlapping the quadrate (0); with sinusoidal ventral edge and wide and rounded posterior edge slightly overhanging the lateral surface of the quadrate (1).

Character 212 (Pol and Norell, 2004a : char. 181): Quadrate body distal to otoccipital-quadrate, orientation of contact in posterior view: ventrally oriented (0); ventrolaterally oriented (1).

Character 213 (Gasparini et al., 1993: char. 3): Wedge-like process of the maxilla in lateral surface of premaxilla-maxilla: absent (0); present (1).

Character 214 (Pol and Norell, 2004b: char. 181): Palpebrals: separated from the lateral edge of the frontal (0); extensively sutured to each other and to the lateral margin of the frontals (1).

Character 215 (Pol and Norell, 2004b: char. 182): External surface of ascending process of jugal: exposed laterally (0); exposed posterolaterally (1).

Character 216 (modified from Pol and Norell, 2004b: char. 183 by Pol et al., 2009 and Turner and Sertich, 2010): Longitudinal ridge on lateral surface of jugal below infratemporal fenestra: absent (0); present, running entire length of of posterior process of jugal (1); present, running entire length of jugal (2).

Character 217 (Pol and Norell, 2004b: char. 184): Dorsal surface of posterolateral region of squamosal: without ridges (0); with three curved ridges oriented longitudinally (1).

Character 218 (Pol and Norell, 2004b: char. 185): Ridge along dorsal section of quadrate-quadratojugal contact: absent (0); present (1).

Character 219 (modified from Pol and Norell, 2004b: char. 186 by Pol et al., 2009): Sharp ridge on the surface of the angular: absent (0); present on the ventral-most margin (1); present along the lateral surface (2).

Character 220 (Pol and Norell, 2004b: char. 187): Longitudinal ridge along the dorsolateral surface of surangular: absent (0); present (1).

Character 221 (Pol and Norell, 2004b: char. 188): Dorsal surface of osteoderms ornamented with anterolaterally and anteromedially directed ridges (fleur de lys pattern of Osmólska et al., 1997): absent (0), or present (1).

Character 222 (Pol and Norell, 2004b: char. 189): Cervical region surrounded by lateral and ventral osteoderms sutured to dorsal elements: absent (0), or present (1).
Character 223 (Pol and Norell, 2004b: char. 190): Appendicular osteoderms: absent (0), or present (1).
Character 224 (Ortega et al., 2000: character 72): Supratemporal fenestra: present (0), or absent (1).
Character 225 (modified from Pol and Apesteguía, 2005: char. 220 and Pol et al., 2009): Flat ventral surface of internal nares septum: parallel sided (0), tapering anteriorly (1), or expanding anteriorly (2).
Character 226 (modified from Pol and Apesteguía, 2005: char. 221): + Perinarial fossa: restricted extension (0); extensive, with distinctly concave surface facing anteriorly (1); large concave surface facing anteriorly, projecting anteroventrally from external nares and opening toward alveolar margin (2); or extremely large and well-developed, occupying nearly entire surface of premaxilla ventral to external naris (3).
Character 227 (modified from Sereno et al., 2001: char. 67): Premaxillary palate, circular paramedian depressions: absent (0); present, located anteriorly on the premaxilla (1); present, located at the premaxilla-maxilla suture (2).
Character 228 (Pol and Apesteguía, 2005: char. 223): Nasals, shape of posterolateral region: flat surface facing dorsally (0); lateral region deflected ventrally, forming part of the lateral surface of the snout (1).
Character 229 (Zaher et al., 2006: char. 193): Lacrimal, posterior extent and relationship with jugal: extends ventroposteriorly, widely contacting the jugal (0); tapers ventroposteriorly, does not contact or contacts the jugal only slightly (1).
Character 230 (Zaher et al., 2006: char. 194): Jugal, large foramen on the lateral surface near the anterior margin: absent (0); present (1).
Character 231 (modified from Zaher et al., 2006: char. 195): Procumbent premaxillary alveoli: absent (0); present (1).
Character 232 (modified from Martinelli, 2003: char. 36 by Zaher et al., 2006: char. 196 and Turner, 2004: char. 119): Palatines, orientation: run parasagittally along midline (0); diverge laterally becoming rodlike caudally forming palatine bars (1).

Character 233 (Zaher et al., 2006: char. 197): Ectopterygoid, participation in the palatine bar: absent (0); present (1).
Character 234 (Pol and Norell, 2004a: char. 183): Choanal opening: opened posteriorly and continuous with pterygoid surface (0); closed posteriorly by an elevated wall formed by the pterygoids (1).
Character 235 (Zaher et al., 2006: char. 198): Ectopterygoid, extent of medial projection on the ventral surface of pterygoid flanges: barely extended (0); widely extended covering approximately the lateral half of the ventral surface of the pterygoid flanges (1).
Character 236 (Gasparini et al., 2006: char. 236): Evaginated maxillary alveolar edges: absent (0); present as a continuous sheet (1); present as discrete evaginations at each alveoli (2).
Character 237 (Gasparini et al., 2006: char. 237): Premaxilla, foramen in perinarial depression: absent (0); present (1).
Character 238 (Sereno et al., 2001: char. 27): Frontal, anterior ramus with respect to the tip of the prefrontal: ending posteriorly (0); ending anteriorly (1).
Character 239 (modified from Sereno et al., 2001: char. 68): Premaxilla, anterior alveolar margin orientation: vertical (0); inturned (1).
Character 240 (modified from Sereno et al., 2001: char. 69): Premaxillary tooth row orientation: arched posteriorly from midline (0); angled posterolaterally, at 120 degree angle (1); transverse (2).
Character 241(Sereno et al., 2001: char. 70): Last premaxillary tooth position relative to tooth row: anterior (0); anterolateral (1).
Character 242 (Gasparini et al., 2006: char. 242): Posterior teeth with rings of undulated enamel: absent (0); present (1).
Character 243 (modified from Brochu, 1999: char. 108 by Gasparini et al., 2006: char. 243): Maxilla-palatine suture, shape of palatines: palatine anteriorly rounded (0); palatine anteriorly pointed (1); palatine invaginated (2).
Character 244 (Gasparini et al., 2006: char. 244): Postorbital bar, lateral surface formed by: postorbital and jugal (0); only by postorbital (1).
Character 245 (Gasparini et al., 2006: char. 245): Surangular groove, enlarged foramen at anterior end: absent (0); present (1).

Character 246 (Gasparini et al., 2006: char. 246): Shape of antorbital fossa: subcircular or subtriangular (0); elongated, low, and oriented obliquely (1).
Character 247 (Gasparini et al., 2006: char. 247): Prefrontal lateral developent: reduced (0); enlarged, extending laterally over the orbit (1).
Character 248 (Gasparini et al., 2006: char. 248): Foramen for the internal carotid artery: reduced, similar in size to the openings of cranial nerves IX-XI (0); extremely enlarged (1).
Character 249 (Gasparini et al., 2006: char. 249): Squamosal posterolateral region, lateral to paroccipital process: narrow (0); bearing a subrounded flat surface (1).
Character 250 (Gasparini et al., 2006: char. 250): Posteromedial branch of squamosal, orientation: transversely oriented (0); posterolaterally oriented (1).
Character 251 (Gasparini et al., 2006: char. 251): Squamosal, dorsal margin of occipital flange: straight (0); dorsally concave (1).
Character 252 (Gasparini et al., 2006: char. 252): Sculpture in external surface of rostrum: absent (0); present (1).
Character 253 (Gasparini et al., 2006: char. 253): Longitudinal depressisons on palatal surface of maxillae and palatines: absent (0); present (1).
Character 254 (Gasparini et al., 2006: char. 254): Angle between medial and anterior margins of supratemporal fossa: approximately 90 degrees (0); approximately 45 degrees (1).
Character 255 (Gasparini et al., 2006: char. 255): Sacral vertebrae, direction of transverse processes: laterally (0); markedly deflected ventrally (1).
Character 256 (Gasparini et al., 2006: char. 256): Prefrontal and lacrimal around orbits: forming flat rims (0); evaginated, forming elevated rims (1).
Character 257 (Gasparini et al., 2006: char. 257): Nasal bones: paired (0); partially or completely fused (1).
Character 258 (Brochu, 1997a: char. 3): Axial neural spines, width of posterior half: wide (0); narrow (1).
Character 259 (Brochu, 1997a: char. 19): Axial hyapophysis, deep fork: present (0); absent (1).

Character 260 (Brochu, 1997a: char. 27): Ulna, width of olecranon process: narrow and subangular (0); wide and rounded (1).
Character 261 (Brochu, 1997a: char. 29): M. teres major and M. dorsalis scapulae insert separately on humerus: scars can be distinguished dorsal to deltopectoral crest (0); insert with common tendon, single insertion scar (1).
Character 262 (modified from Brochu, 1997a: char. 53): Dentary, projection of anterior alveoli: anterodorsally (0); weakly procumbent (1); strongly procumbent (2).
Character 263 (Brochu, 1997a: char. 84): Squamosal, dorsal and ventral rims of squamosal groove for external ear valve musculature: parallel (0); or squamosal groove flares anteriorly (1).
Character 264 (Brochu, 1997a: char. 91): Ectopterygoid, contact with maxilla near toothrow: ectopterygoid abuts maxillary toothrow (0); maxilla broadly separates ectopterygoid from maxillary toothrow (1).
Character 265 (Brochu, 1997a: char. 92): Shallow fossa at anteromedial corner of supratemporal fenestra: present (0); absent, anteromedial corner of supratemporal fenestra smooth (1).
Character 266 (modified from Brochu, 1997a: char. 103 by Pol et al., 2009): Lateral margins of the frontal, relative to the skull surface: flush with skkull surface (0); elevated, forming ridged orbital margins (1).
Character 267 (Brochu, 1997a: char. 130): Laterosphenoid, orientation of capitate process: laterally oriented (0); anteroposteriorly oriented toward midline (1).
Character 268 (modified from Brochu, 1997a: char. 141 by Pol et al., 2009): Exoccipital, development of boss and paroccipital process: boss prominent on paroccipital process, process lateral to cranioquadrate opening short (0); boss small or absent on paroccipital process, process lateral to cranioquadrate opening long (1).
Character 269 (modified from Norell, 1988: char. 32 by Brochu, 1997a: char. 149): Ectopterygoid, extent along lateral pterygoid flange, at maturity: extends to posterior tip of lateral pterygoid flange (0); does not extend to posterior tip of lateral pterygoid (1).
Character 270 (modified from Brochu, 1997a: char. 153): Incisive foramen, location relative to premaxillary toothrow: foramen situated far from premaxillary toothrow, at the

level of the second or third alveolus (0); abuts premaxillary toothrow (1); projects between first premaxillary teeth (2).

Character 271 (modified from Turner, 2006: character 126 by Pol et al., 2009 by Pritchard et al., 2013): Ventral surface of choanal septum: smooth to slightly depressed (0); marked by a acute groove (1); vomeral septum divided by into bilateral laminae (2).

Character 272 (modified from Turner, 2006 char. 128 by Pol et al., 2009):Proximal-most portion of fibular head: straight sided to weakly developed posteriorly (0); very sharply projecting posteriorly, forming distinct extension (1).

Character 273 (modified from Turner, 2006: char. 129): Cervical rib shaft, posterior process, posterodorsally projecting spine at the junction with the tubercular process: absent (0); present (1); State 2 (2).

Character 274 (modified from Pol et al., 2009: char. 274): Longitudinal keels on dorsal surface of osteoderms: restricted to the posterior edge of osteoderm (0); not restricted to the posterior edge (1).

Character 275 (Pol et al., 2009: char. 275): Jugal, anteriorly on lateral surface below orbits: lacks a depression (0); possesses a depression (1).

Character 276 (modified from Pol et al., 2009: char. 276 by Pritchard et al., 2013): Transverse ridge crossing the frontal anteromedial to the orbits: absent (0); present as a ridge (1); present as prominent anteriorly-curved shelf (transverse interorbital crest sensu Andrade and Hornung 2011) (2); present as anteroposteriorly-oriented crest on frontal (3).

Character 277 (Pol et al., 2009: char. 277): Shallow hemispherical depression on the lacrimal and/or prefrontal anterior to the orbital margin (not articulation facet for palbebral): absent (0); present (1).

Reinterpretation of the morphology in *Shamosuchus djadochtaensis* suggests that the depression coded for in this character is very similar to the depression present for articulation of a palpebral. Additionally work is need to clarify this morphology. Until that time, we have chosen to exclude this character during tree searches.

Character 278 (Pol et al., 2009: char. 278): Anterior half of interfenestral bar between suborbital fenestrae: lateral margins are parallel to subparallel (0); flared anteriorly (1).

Character 279 (Pol et al., 2009: char. 279): Posterior half of interfenestral bar between suborbital fenestrae: lateral margins are parallel to subparallel (0); flared posteriorly (1).
Character 280 (Pol et al., 2009: char. 280): Angular, shape of posteroventral margin: straight or gently arched dorsally (0); strongly arched dorsally (1).
Character 281 (Pol et al., 2009: char. 281): Squamosal, lateral margin of dorsal surface: squared off with continuous ear valve groove (0); bears a prominent depressed area just anterior to the posterior lobe of the squamosal, groove for ear valve discontinuous (1).
Character 282 (Pol et al., 2009: char. 282): Fibula, shaft distal to iliofibularis trochanter: straight (0); bowed posteriorly (1).
Character 283 (Buckley and Brochu, 1999: char. 106): Scapular blade width: no more than twice the length of the scapulocoracoid articulation (0); scapular blade very broad and greater than twice the length of the scapulocoracoid articulation (1).
Character 284 (modified from Buckley et al., 2000: char. 115 by Pritchard et al., 2013): Vomer, exposure on palate: vomer contributes flattened plate to secondary palate (0); vomer forms no part of secondary palate (1).
Character 285 (Turner and Buckley, 2008: char. 285): Supraoccipital, when present on dorsal skull roof: with narrow exposure, parietal forms portion of occipital surface (0); with broad exposure, parietal does not form portion of occipital surface (1).
Character 286 (Turner and Buckley, 2008: char. 286): Jugal, anterior and posterior processes: inline dorsoventrally (0); anterior and posterior processes at a sharp angle to one another, both processes slope ventrally to form a strongly arched jugal (1).
Character 287 (Turner and Buckley, 2008: char. 287): Lateral expansion of posterodorsal edge of surangular anterior to glenoid fossa: absent (0); present (1).
Character 288 (Turner and Buckley, 2008: char. 288): In lateral view, anterior process of the squamosal extending to the orbital margin, overlapping the postorbital: absent (0); present (1).
Character 289 (Turner and Buckley, 2008: char. 289): In lateral view, surangular and dentary suture: simple with little or no interdigitating (0); suture complex with interlocking prongs from both surangular and dentary, three posterior prongs from the dentary and two form the surangular (1).

Character 290 (Turner and Buckley, 2008: char. 290): Prominent depression on the palate near alveolar margin at the level of the 6th or 7th alveoulus: absent (0); present (1).
Character 291(Andrade and Bertini, 2008a: char. 103): Pterygoid, ventral surface of pterygoid flanges, parachoanal fossae: absent (0); present (1).
Character 292 (Turner and Sertich, 2010: char. 292): Pterygoid, in ventral view, participation in the suborbital fenestra: pterygoid forms margin of suborbital fenestra (0); excluded from suborbital fenestra (1).
Character 293 (Turner and Sertich, 2010: char. 293): Maxilla, lateral surface along alveolar margin, conformation of the neurovascular foramina: foramina absent or form a single continuous row (0); gap in foramina between an anterior series and a posterior series (1).
Character 294 (Turner and Sertich, 2010: char. 294): Surface of tooth enamel: smooth or slightly crenulated (0); with ridges at base of crown (1).
Character 295 (Turner and Sertich, 2010: char. 295): Posterior (molariform) teeeth, wear facets: absent (0); present (1).
Character 296 (Turner and Sertich, 2010: char. 296): Tooth (with transitional morphology) present at premax-max contact absent (0); present (1).
Character 297 (Turner and Sertich, 2010: char. 297): Basiocciptal, midline crest on basioccipital plate below occipital condyle absent (0); present (1).
Character 298 (Turner and Sertich, 2010: char. 298): Dorsal osteoderms, accessory ranges of osteoderms (sensu Frey, 1988) absent (0); present (1).
Character 299 (Andrade and Bertini, 2008a: char. 131): Maxillary tooth, size relative to maxillary palatal surface in palatal view proportionally small teeth, occupying only marginal portion of ventral surface of maxilla (0); proportionally well developed teeth, occupying large area of maxillary palatal surface (1).
Character 300 (Jouve, 2009:character 75; Jouve, 2004: character 68): Ventral lamina of jugal extends far anterior to the ectopterygoid (0); ends at the level of the ectopterygoid (1).
Character 301 (Sereno and Larsson, 2009: character 199; adapted from Norell, 1988: character 42 and Brochu, 1997: character 51): Surangular extension toward posterior end of retroarticular process along entire length (0); pinched off anterior to posterior tip (1).

Character 302 (new): Muscle attachment scars on ventral surface of quadrate ramus: form modest crests (0); prominent knobs (1).
Character 303 (new): Pterygoid flange shape: mediolaterally broad, reaching laterally beyond medial margin of quadrate condyles (0); relatively narrow, does not reach laterally to medial margin of quadrate condyles (1).
Character 304 (new): In ventral view, posterior process of maxilla relative to ITF excluded from ITF (0); forms part of ITF (1).
Character 305 (new): Highly modified ectopterygoid, mediolaterally broad and flattened with greatly expanded: absent (0); present, robust anterior process larger than posterior process (1); present, anterior and posterior process roughly equal in size (2).
Character 306 (new): In ventral view, palate medial to toothrow: forms a single continuous surface (0); ridge running on the palate medial to toothrow formed by maxilla and ectopterygoid (1).
Character 307 (new): Maxillary tooth row, penultimate and ultimate maxillary teeth enlarged and highly modified crushing tooth: absent (0); present (1).
Character 308 (new): Prefrontals: do not meet at midline (0); meet at midline (1).
Character 309 (new): Pear shaped external naris: absent (0); present (1).
Character 310 (new): Skull, dorsal surface at parietal-squamosal contact surface: continuous across suture (0); suture marked by groove or sulcus (1).
Character 311 (new): Maxilla, lateral surface, continuous groove or sulcus extending from orbital margin to near orbital margin towards narial opening: absent (0); present (1).
Character 312 (new): Maxilla, posteromedial process curving posteriorly onto palatine-formed nasopharyngeal passage: absent (0); present (1).
Character 313 (new): Squamosal, posterior half, dorsal and ventral rims of groove for external ear valve musculature: thin or parallel sided (0); flared posteriorly (1).
Character 314 (new): Lacrimal, in dorsal view, anterior extent on rostrum relative to prefrontal: prefrontal extends farther anteriorly (0); lacrimal extends farther anteriorly (1); lacrimal and prefrontal subequal in anterior extent (2).
Character 315 (new): Lacrimal, in dorsal view, mediolateral width relative to prefrontal: equal to or less than width of prefrontal (0); wider than prefrontal (1).

Character 316 (new): Premaxillae, degree of contact posterior to the incisive foramen: extensive contact (0); narrow contact (1).
Character 317 (new): Posterior margin of the palatines where they form the floor of the nasopharyngeal passage, shape: V- or U-shaped (0); straight (1).
Character 318 (new): Posterior margin of the choanal groove, location: anteriorly on the pterygoids (0); posteriorly on the pterygoids near the posterior margin of pterygoids (1).
Character 319 (new): Pterygoid-palatine contact, ventral aspect of palate, shape of the suture: transverse, or nearly so (0); prong of pterygoid projects anteriorly (1).
Character 320 (new): On palate, foramen located on premaxilla/maxilla suture near the alveolar border: absent (0); present (1).
Character 321 (new): Ectopterygoid/pterygoid contact in ventral view: complex, anterior part of ectopterygoid forming suture whereas posterior part of ectopterygoid overlaps the pterygoid (0); sutured along entire contact, no part of ectopterygoid overlapping pterygoid (1).

SUPPLEMENTARY APPENDIX 3. Analysis details for the constrained parsimony analysis and the Bayesian inference analysis.

A single constrained search was conducted. Five nodes were constrained such that the following relationships would be present in the optimal trees: ((Crocodylia + Hylaeochampsidae) + (*Theriosuchus* + Paralligatoridae)). Thus, a traditional Eusuchia was constrained to be monophyletic with Paralligatoridae + *Theriosuchus* as its sister taxon. Maximum parsimony analysis recovered 212 optimal trees with a length of 1664. The strict consensus is depicted in figure S1. The file to replicate the constraint analysis is available on Morphobank under “Documents” .

Bayesian inference trees were estimated using MrBayes v3.2 (Ronquist et al., 2012). The Standard model (Markov k-state variable model [Mkv] with a gamma-distributed rate variation) was specified for the phenotypic dataset. During the analysis, MCMC chain convergence was assessed using the average standard deviation of split frequencies and examing trace files in Tracer (Rambaut and Drummond, 2007). Convergence to stationary was assumed for split frequencies below 0.01 and ESS values > 200 (citation). The MCMC was run for 5 million generations sampling parameters

every 1000 generation. The first 25% of samples were discarded as burn-in. A maximum clade credibilty tree is shown in figure 2S. The file to replicate the BI analysis is available on Morphobank under "Documents".

Ronquist F., M. Teslenko, P. Van Der Mark, D.L. Ayres, A. Darling, et al. 2012. MrBayes 3.2: Efficient Bayesian phylogenetic inference and model choice across a large model space. Systematic Biology 61: 539–542.

Rambaut A., and A.J. Drummond. 2007. Tracer v1.4, Available from http://beast. bio.ed.ac.uk/Tracer.

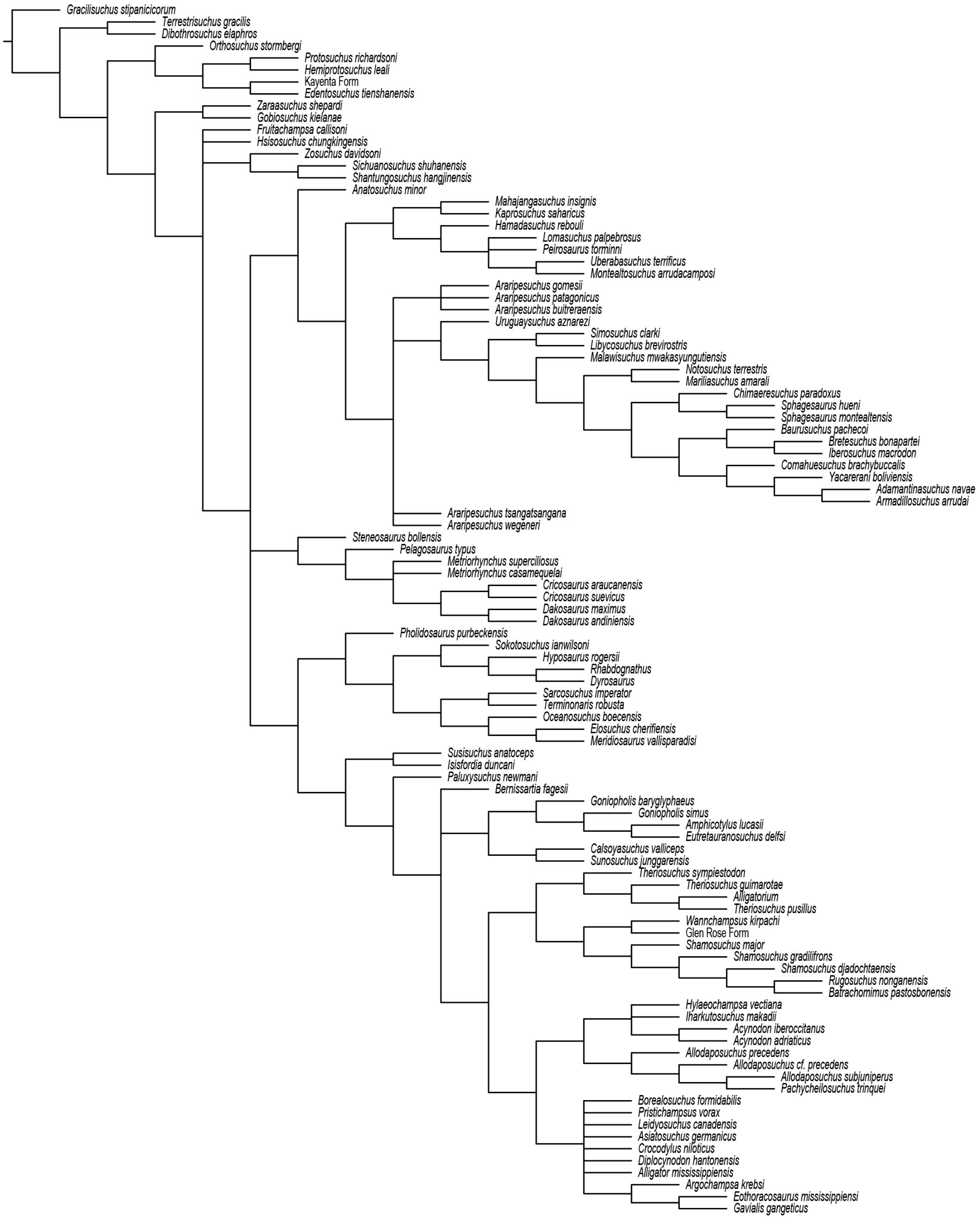

Figure 1S. Strict consensus of 212 most parsimonious trees (TL=1664) resulting from the constrained search detailed in supplementary appendix 3.

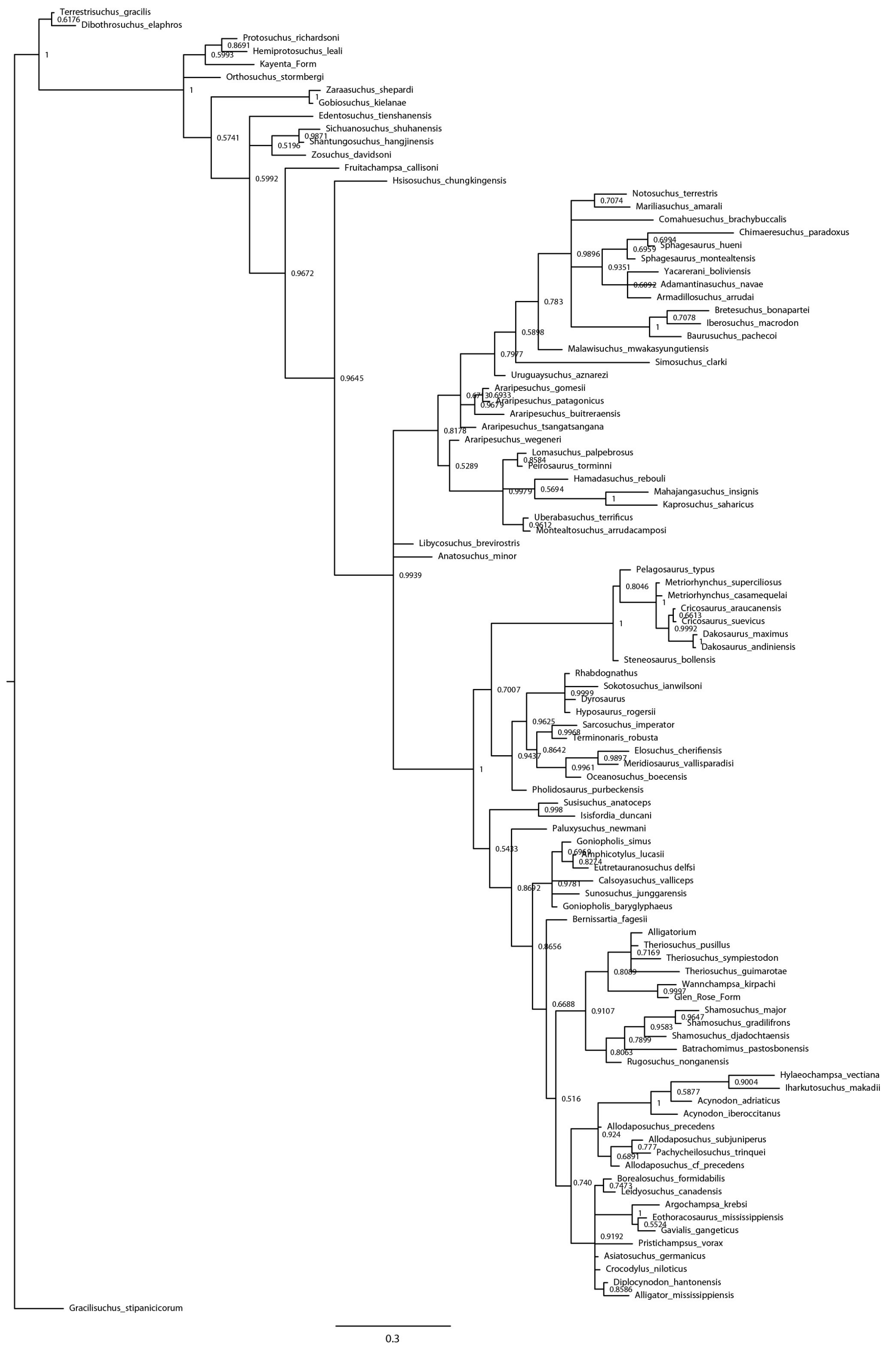

Figure 2S. Maximum clade credibility tree resulting from the Bayesian inference analysis detailed in supplementary appendix 3. Numbers by nodes are posterior probabilities. Scale bar equals branch length as amount of character change.
